# Tracking development assistance for health, 1990–2030: historical trends, recent cuts, and outlook

**DOI:** 10.1016/S0140-6736(25)01240-1

**Published:** 2025-07-15

**Authors:** Angela E Apeagyei, Catherine Bisignano, Hans Elliott, Simon I Hay, Brendan Lidral-Porter, Seong Nam, Carolyn Shyong, Golsum Tsakalos, Bianca S Zlavog, Enis Barış, Christopher J L Murray, Joseph L Dieleman

**Affiliations:** 1 Institute for Health Metrics and Evaluation (IHME); 2 Department of Health Metrics Sciences, School of Medicine, University of Washington

## Abstract

**Summary:**

Development assistance for health (DAH) rose to its highest levels during the COVID-19 pandemic but has since reduced amid rising global economic uncertainty and competing fiscal demands. In early 2025, major donors including the USA and the UK announced substantial reductions in aid, prompting concerns about the future of health financing in middle- and low-income countries. To date, no comprehensive assessment has quantified the effects of these announced cuts on overall DAH levels or its future trajectories.

**Methods:**

We estimated DAH from 1990 to 2030, drawing from a wide range of data sources including the Organisation for Economic Co-operation and Development Creditor Reporting System database, online databases of agencies such as The Global Fund to Fight AIDS, Tuberculosis, and Malaria and the Global Alliance for Vaccines and Immunization (Gavi), and financial reports from private philanthropies and non-governmental organisations. Disbursements were categorised by source, disbursing agency, health focus area, and recipient country using standardised keyword tagging methods developed over the 15 years of Institute for Health Metrics and Evaluation Financing Global Health reports. For 2025, we incorporated budget cuts announced by major donors to develop preliminary estimates. Forecasts to 2030 used donor-specific funding targets and linear regression models. Additional refinements for this iteration of our DAH tracking included expanded donor coverage and disaggregation of health areas for additional disbursing entities.

**Findings:**

DAH peaked at US$80.3 billion in 2021 and fell to $49.6 billion in 2024. In 2025, announced budget cuts—particularly reductions in US bilateral aid—are expected to contribute to further declines in global DAH to $38.4 billion, levels last seen in 2009. Key global health institutions (e.g., Foreign, Commonwealth & Development Office and US Agency for International Development and Agence Française) providing DAH fighting key infectious diseases and providing childhood vaccines will contract their own disbursements. Because the key multilateral development banks have been protected from the major funding cuts, the World Bank has increased its relative share of total DAH disbursements. Forecasts indicate continued stagnation in DAH through 2030 under current policies, reaching $36.2 billion in 2030.

Our sensitivity analyses suggest that our estimate for 2025 could range from $36.8 billion in a pessimistic scenario to $40.0 billion in an optimistic scenario based on changes in US cuts. Similarly, in the next five years, total DAH is expected to reach $37.8 billion in 2030 under a positive scenario for US contribution and $34.5 billion under a negative scenario for US contribution.

**Interpretation:**

The global health financing landscape is entering a period of sustained cuts. Major reductions in DAH, particularly from historically leading donors, threaten to widen health disparities unless mitigated by increased domestic resource mobilisation or alternative financing mechanisms. This study highlights the urgent need for greater efficiency, strategic reprioritisation, and strengthened fiscal resilience in recipient countries to safeguard the substantial global health gains of the previous three decades.

## Introduction

Although development assistance for health (DAH) rose to historic levels during the COVID-19 pandemic years, it has since been reduced to pre-pandemic levels.^1,2^ Issues such as the Russia-Ukraine war and growing concerns about the effect of climate change have been at the forefront of global financing news, contributing to increased uncertainty in global health spending.^3,4^ Furthermore, 2025 has heralded a dramatic season of austerity for DAH, with the announcement of substantial cuts in aid from most major providers.^5–7^

Since the cuts were announced, various estimates of lives to be lost, services to be cut, and jobs to be lost have been published and widely circulated.^8–10^ However, there are no comprehensive assessments of how cuts from individual nations will impact the total amount of DAH provided.^11,12^ It is important to comprehensively characterise the immediate and long-term changes these cuts will have on the global health financing landscape. Such characterisations will help to improve understanding of the impact of the recent changes and to help identify possible opportunities for synergy, efficiency, or collaboration to cover the gap in funding that may result from these changes.

This study fills this knowledge gap by generating comprehensive estimates of DAH up to 2024, with preliminary estimates for 2025, and forecasts to 2030. We leveraged and updated existing databases, developed and annually updated since 2009, and incorporated up-to-date information about government budgets and expected reductions in DAH.^13^ These disaggregated estimates of health spending enable characterisation of the trends and patterns now and in the future for global health financing.

## Methods

### Overview

We estimated DAH from 1990 to 2030. Data were obtained from the Organisation for Economic Co-operation and Development’s (OECD) Creditor Reporting System (CRS), multilateral and bilateral agencies, non-governmental organisations (NGOs), foundations, and national budgets. Disbursements were classified using keyword algorithms and adjusted to avoid double counting as previously described.^2,14,15^ Preliminary estimates for 2025 incorporated publicly announced donor spending plans; forecasts to 2030 used donor-specific details and, for sources of DAH that were not countries, linear regression models to make short term projections. Methodological enhancements included expanded donor coverage and greater granularity in health focus area tracking. (full details are in the appendix pp 27–29).

### Estimating development assistance for health, 1990–2024

We tracked DAH resources from 1990 to 2024. We used project descriptions and disbursement data from online databases of international development agencies such as OECD CRS; The Global Fund to fight AIDS, Tuberculosis and Malaria; Gavi, the Vaccine Alliance; UN Population Fund; multilateral organizations such as the World Bank, regional development banks, private philanthropies; and NGOs. We also leveraged income and expenditure information from financial statements, annual reports, and curated data from correspondents.

We tracked resources from their originating source through the disbursing entity to the disease area of focus or receiving entity. The estimates were disaggregated into ten health focus areas and 61 programme areas. This disaggregation was completed by leveraging a keyword search that tagged words relevant for health focus and programme areas. These were then categorised according to the respective areas. The list of keywords and their assigned health focus and programme areas used are provided in the appendix (pp 37–44). Furthermore, to ensure that each resource flow was accounted for only once and to avoid double counting flows, we used available financial documents on revenue sources to remove resources transferred through multiple disbursing entities. We also included administrative costs of managing the loans and grants. While the OECD Development Assistance Committee (DAC) and CRS databases remain the most widely used, earlier years of the CRS data have some missing data; as such, we used adjusted commitment data from the DAC tables to estimate disbursements as needed. Furthermore, for some disbursing entities, recent years of disbursement data were not complete; in these instances, we relied on publicly available budget data to generate estimates. We provide a detailed description of the process for estimating disbursements for these agencies in the appendix (pp 49–79), which has also been previously published.^2,16^

For this update to the dataset, we made the following improvements. First, we further disaggregated the sources of funding for the multilateral development banks. Second, we harnessed additional granular data from the International Aid Transparency Initiative and AidData to calculate health focus and programme area allocations.^17–19^ Lastly, we incorporated eight new countries (Czechia, Estonia, Hungary, Iceland, Lithuania, Poland, Slovakia, and Slovenia) that have joined the OECD DAC since 2013.^20^

### Generating preliminary estimates of development assistance for health for 2025

To generate a preliminary estimate of DAH for 2025, we utilised two data sources: our estimate of development assistance for 2024; and information from budgets, press-releases, statements from government officials, and news articles about future development assistance allocations from the major donors (appendix pp 98–101). We extracted the specific details of intended official development assistance cuts and directly applied the proportional percentage cut across all entities for which the donor source provided DAH.

For example, the USA has historically provided 20–25% of all DAH and has proposed the most immediate and extreme reductions in development assistance. Initial reports indicated plans to cut 90% of United States Agency for International Development (USAID) projects, while the preliminary budget proposal from the White House provided evidence of less severe cuts.^2,21–23^ To estimate the preliminary US DAH contribution for 2025, we cut funding provided from the USA to all entities inline with the proposed budget. Exceptions include instances where there was explicit information of the total committed amount or specific information about the magnitude of cuts. We used a similar method for all donors for whom aid cuts have been announced.

### Forecasting development assistance for health from 2026 through 2030

DAH was forecasted for 2026–2030 for each major source, including 24 national governments, private philanthropy (other than the Gates Foundation), the Gates Foundation, debt repayments, other OECD DAC countries, and other non-DAC countries. National governments were broken into two groups. Countries that have made public statements indicating that their development assistance contributions would increase with gross national income (GNI) were assumed to have their DAH contributions grow at the same rate as GNI is projected to grow (appendix p 108). Countries for whom we could not find public statements aligning their development assistance contributions with GNI were assumed to have constant DAH disbursements (in real inflation-adjusted terms) for 2025 through 2030. Similarly, the Gates Foundation, was held constant at $9 billion in real terms from 2025 to 2030 which aligns with the recent announcement of providing $200 billion in support over the next twenty years.^24,25^ DAH from private foundations (other than the Gates Foundation) were assumed to be responsive to government funding of DAH, and thus estimates through 2030 were made by regressing private foundation DAH on the sum of all national government’s DAH disbursements. All other sources of DAH, including debt repayments, other OECD DAC countries, and non-DAC countries,were projected using linear regression models.

### Sensitivity analysis for 2025 estimates and forecasts

Given the uncertainty surrounding announced cuts by the USA in particular, we explored alternative estimated scenarios in which we varied the magnitude of reductions aid disbursements for the USA. We refer to the estimates we produced based on the cuts that were initially announced by the USA as our reference scenario. In this reference scenario, DAH from the US government to most channels is cut by 62% relative to 2024, with some channels receiving smaller or larger cuts based on available information. We developed an alternative US positive scenario in which we applied 9% cuts instead of 62%, on the assumption that some of the proposed cuts could be pushed back during the legislative process and at the level of cuts proposed for rescission. We also developed an alternative US negative scenario, wherein we applied 80% cuts instead of 62%, based on initial reports that indicated that over 80% of USAID programmes would be cancelled.

### Reporting

All values are reported in inflation-adjusted 2023 US dollars unless otherwise specified. To report DAH estimates in 2023 inflation-adjusted dollars, we took disbursements in nominal US dollars in the year of disbursement and utilised USA gross domestic product (GDP) deflators from the International Monetary Fund (IMF) World Economic Outlook (WEO) database to convert the series to constant 2023 US dollars.^26^ To adjust for inflation for the historical and future DAH estimates, we used country-specific exchange rate data and deflator series from IMF WEO to convert the series to constant 2023 US dollars. To quantify the impact of DAH reductions on health sector financing within each recipient country, we estimated the reduction in DAH relative to total health spending in 2022, which is the last year total health spending was observed.

### Role of the funding source

The funder of this study had no role in study design, data collection, data analysis, data interpretation, or writing of the report. AEA and JLD, as corresponding and senior authors, had final responsibility for the decision to submit for publication.

## Results

**Figure 1:**
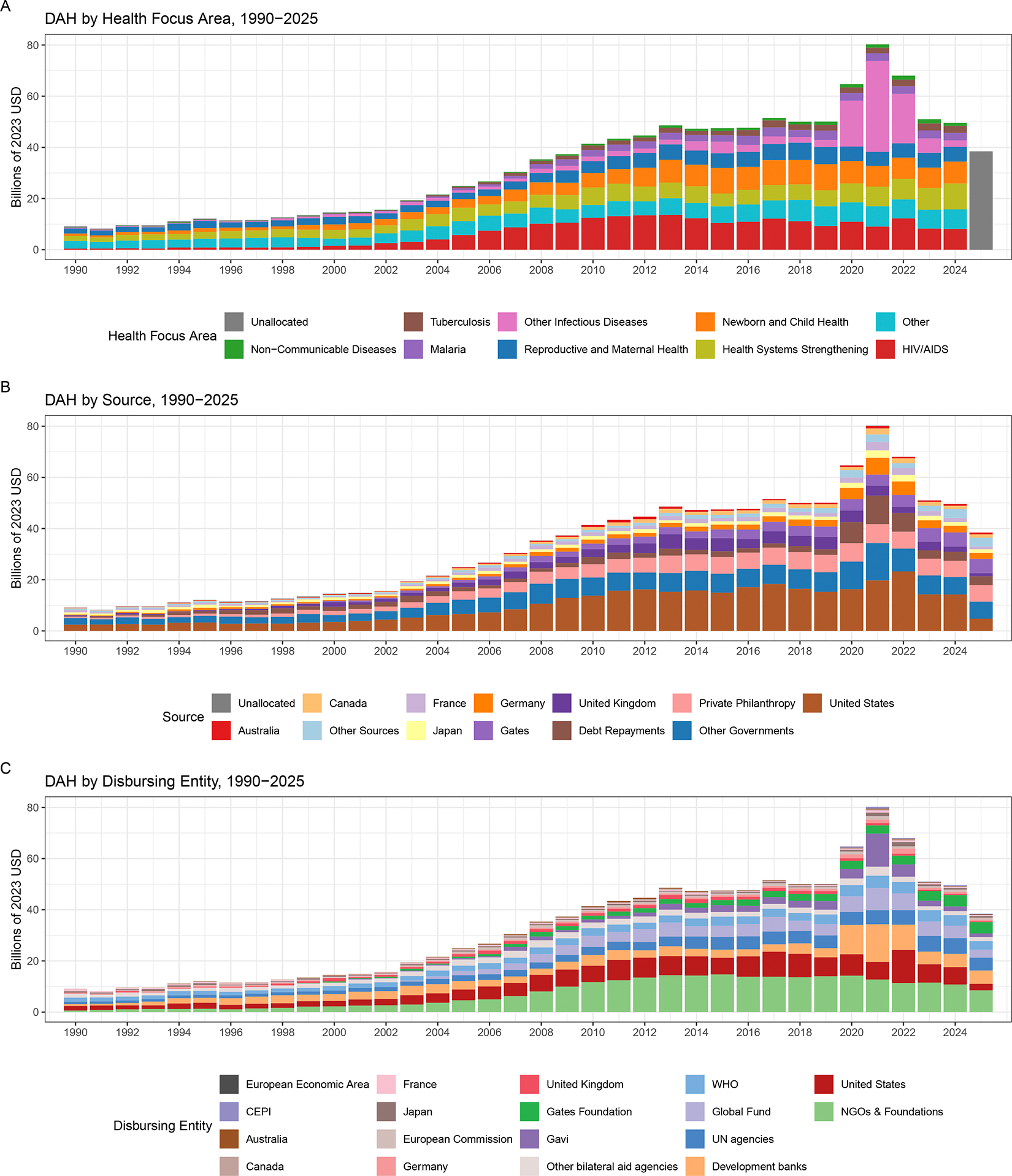
Total development assistance for health by health focus area (A), source (B), and disbursing entity (C), 1990–2025 Development assistance for health is measured in 2023 real US dollars, with 2025 being preliminary estimates. CEPI=Coalition for Epidemic Preparedness Innovations, UN = United Nations, WHO = World Health Organization, NGOs=non-governmental organisations, USD = US dollars.

Total DAH reached an estimated $38.4 billion in 2025, a 52% decline from the peak of $80.3 billion in 2021 and a 22% decline from $49.6 billion in 2024, to a level comparable to the amount provided in 2009 (figure 1, table 1). When disaggregated by health focus, we observed that in the 2000s, which coincided with a greater portion of the era of the Millenium Development Goals (MDGs), HIV/AIDS received $68.0 billion (24%), newborn and child health received $39.2 billion (14%), and health system strengthening (activities targeting broader health sector strengthening such as human resource management, monitoring, and evaluation activities) received $50.7 billion (18%) of the cumulative total development assistance for health ($282.1 billion), in accordance with the areas that had been prioritised in the MDGs (figure 1). Although the total DAH remained relatively unchanged between 2011 and 2019, it increased dramatically in 2020–2022 (to a peak of $80.3 billion in 2021) in response to the COVID-19 pandemic. This is particularly evident in the spike in other infectious disease support in these years, for which substantial new and repurposed funds were mobilised (figure 1). While the decrease in DAH from 2024 to 2025 was notable, DAH was already declining towards pre-pandemic levels by 2023.

**Figure 2.**
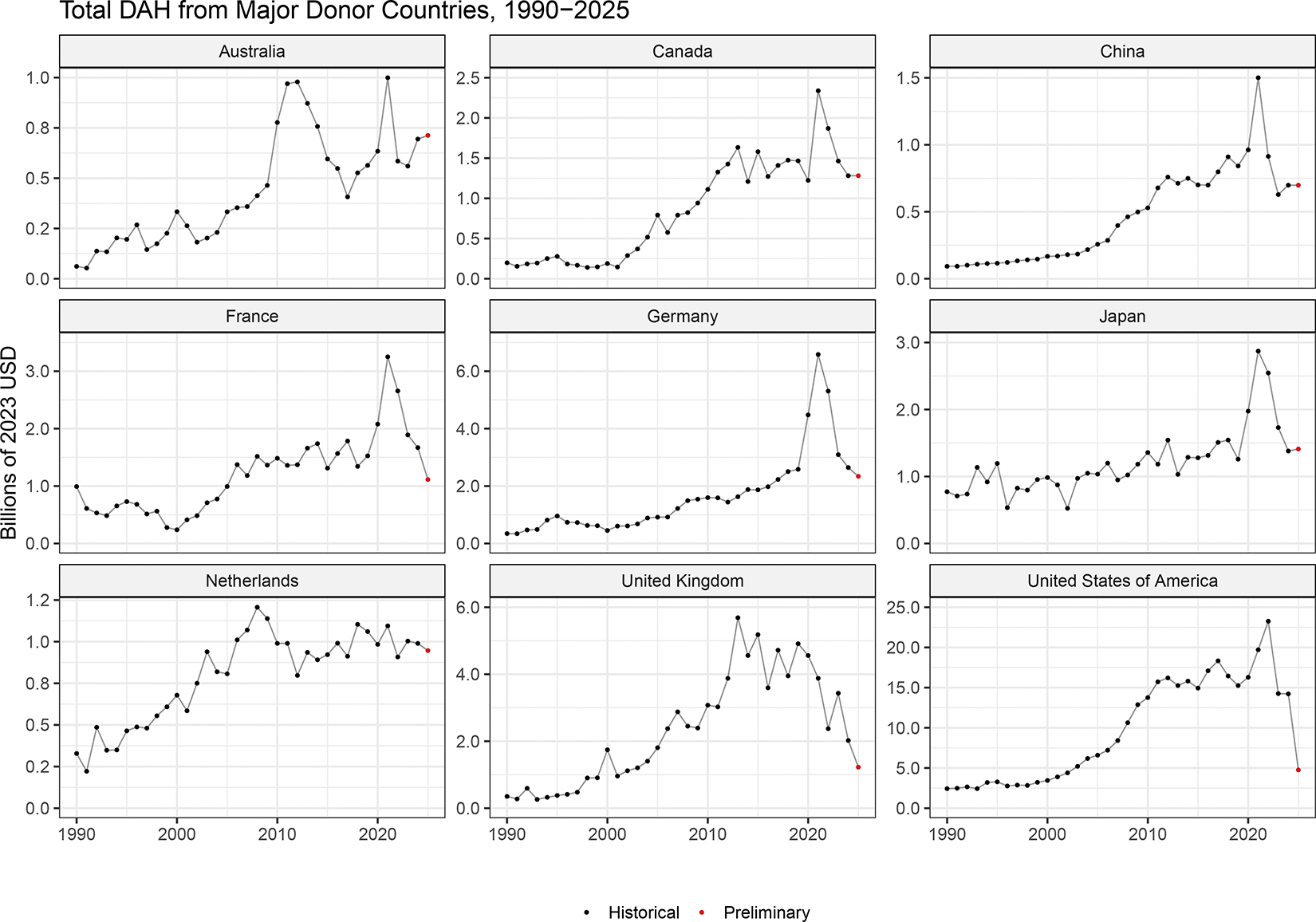
Total development assistance for health provided by major country donors, 1990–2025 Development assistance for health is measured in 2023 real US dollars, with 2025 being preliminary estimates.

When disaggregated by source, the USA historically lead in total DAH provided (average of 30% [$12.6 billion] of total DAH each year between 2000 and 2024). Between 2010 and 2024, the USA provided an average of $16.4 billion per year. 2025 shows a significantly lower level of disbursements, at $4.76 billion (figure 1). While 2025 cuts in US DAH are by far the largest, in both absolute and relative terms, multiple governments that provide DAH have also announced cuts in development assistance and are expected to decrease in 2025 (figure 2). Our preliminary estimates suggest that, as a source, inclusive of transfers to multilateral aid agencies and administrative costs, the UK government will cut disbursements from $2.0 billion (2024) to $1.2 billion (2025), the French government will cut disbursements from $1.7 billion to $1.1 billion, the German government will cut disbursements from $2.6 billion to $2.3 billion, and the Finnish government will cut disbursements from $136.8 million to $122.0 million. Disbursement from other non-DAC donors is estimated to remain relatively stable. For example, China is estimated to provide $698.4 million in 2025, comparable to levels in 2024. Similarly, the United Arab Emirates, included as part of other donors, is estimated to provided $49.1 million, comparable to 2024 levels. The aggregate total of the eight countries (Czechia, Estonia, Hungary, Iceland, Lithuania, Poland, Slovakia, and Slovenia) that were recently added to our database is $164.3 million in 2025.

Bilateral aid agencies (e.g., Foreign, Commonwealth & Development Office and USAID and Agence Française) operated by DAC member governments are expected to reduce spending at or above the rate that donor countries are expected to cut their total DAH (figure 1; table 1). How multilateral aid agencies and private-public partnerships dip into any reserves to maintain funding in the short run is difficult to predict but cuts from the largest public donors are likely to have substantive reductions in disbursements. Our preliminary estimates are that The Global Fund will contract disbursements from $4.9 billion (2024) to $3.1 billion (2025) and Gavi will reduce spending from $1.8 billion to $1.4 billion. The World Bank’s International Development Agency and International Bank of Reconstruction and Development are expected to have smaller reductions, as much of their funding is from debt repayments or long-standing commitments (table 1). Preliminary estimates suggest that DAH from the World Bank will remain largely constant ($3.79 billion in 2024 to $3.76 billion in 2025) while spending at the regional development banks is expected to remain relatively constant. Many international NGOs providing funds and services in low-income and middle-income countries receive financial support from government partners, especially from the USA. As such, our preliminary estimates suggest that DAH disbursements from NGOs will shrink from $10.4 billion in 2024 to $8.1 billion in 2025.

In 2025, the preliminary estimates suggest that the leading source–disbursing entity flow will be DAH funds sourced from private philanthropy and disbursed through NGOs and foundations (excluding the Gates Foundation), contributing $4.86 billion in total DAH (table 1). Funds sourced from and disbursed by the Gates Foundation are second, contributing $4.42 billion to total DAH, followed by other sources (mainly debt repayments) disbursed through development banks ($3.92 billion). Among the seven leading donor countries, the largest flow is expected to occur from the USA through its bilateral agencies ($2.56 billion), followed by the US disbursement through NGOs & Foundations ($1.22 billion) and the Global Fund ($800 million), and then Japan ($657 million) as both the source and disbursing entity. The appendix (pp 98–101) provides additional detail highlighting the relative changes between 2024 and 2025 by source-disbursing entity.

**Figure 3:**
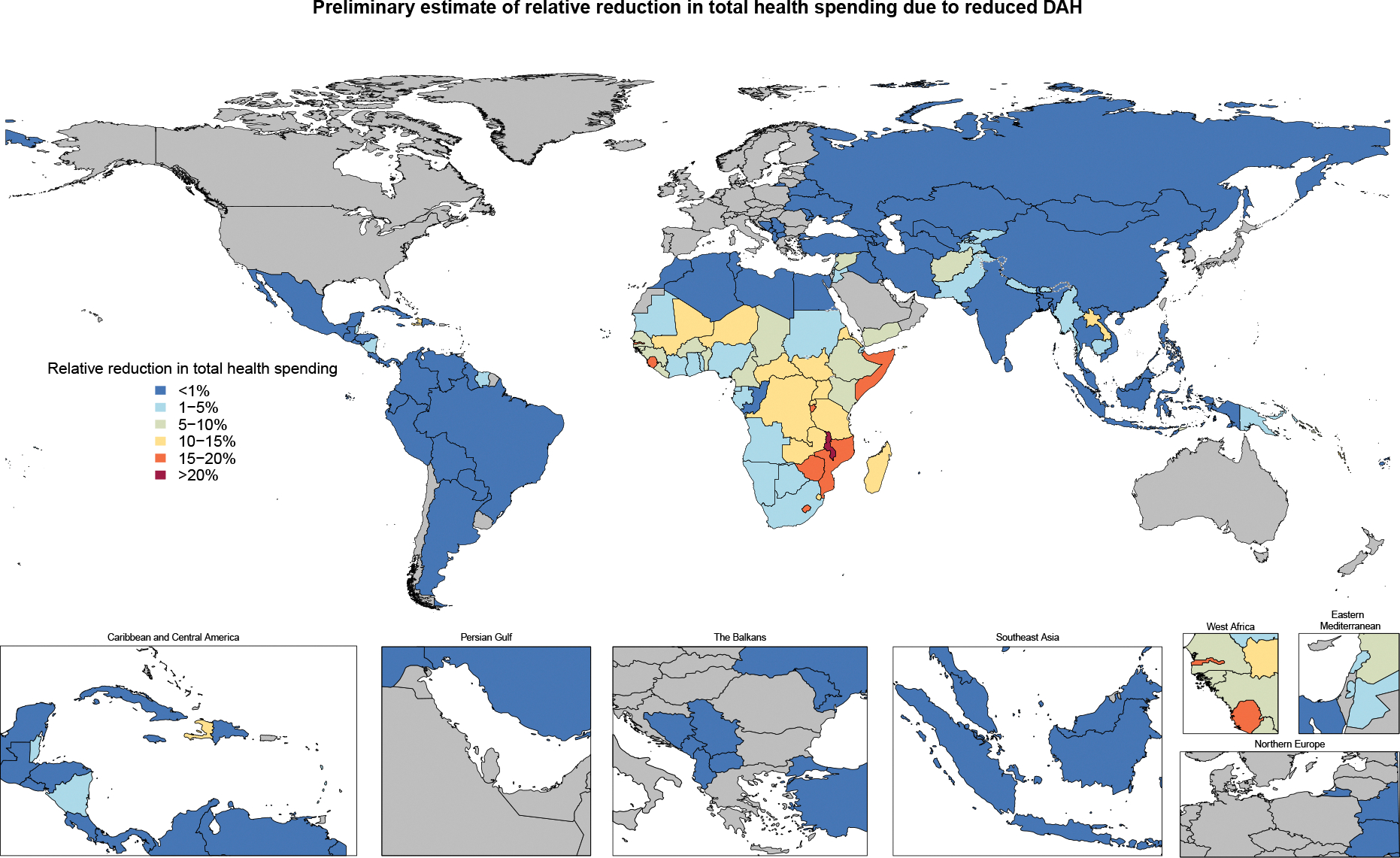
Relative reduction in total health spending, 2022 to 2025 Relative reduction in DAH relative to total health spending in 2022.

**Figure 4:**
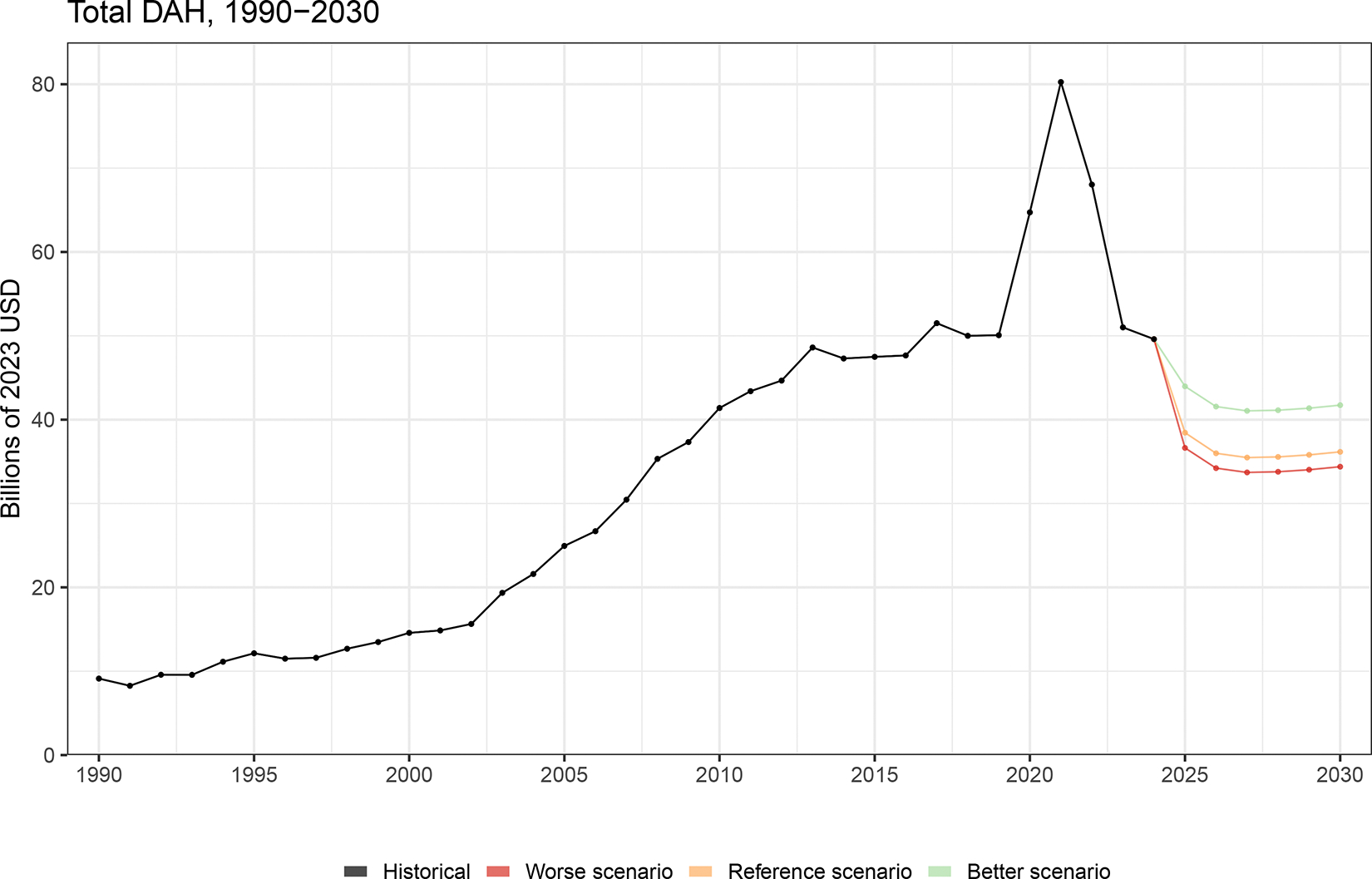
Historical and forecasted total development assistance for health (DAH), 1990–2030 Development assistance for health is measured in 2023 real US dollars, with 2025 being preliminary estimates and 2026 through 2030 being forecasted based on different scenarios.

On average, the effect of DAH cuts in the health sector in 2025 versus 2022, as reflected in the relative percent change in total health spending between the 2 years, will be relatively small (a 0.3% or $71.5million average reduction), but for low-income countries, a 6% ($84.9 million) average reduction in total health spending is expected (figure 3). 2022 is used a comparator as it is the most recent year with complete and comparable estimates of total health spending data. Some of the countries who are expected to see the greatest relative declines in their national health budget are DR Congo, South Sudan, Zambia, Mozambique, and Malawi. These countries have high reliance on DAH from donors that are cutting DAH the most. Within sub-Saharan Africa, the region where the majority of country-specific DAH has been targeted, there are some countries (e.g., South Africa, Gabon, Kenya, and Ghana) that are expected to be more resilient to the cuts because they have invested their own domestic resources into their health system. Despite this resilience, these countries remain at high risk as they are also some of the countries that receive the most DAH in per person terms. Our DAH forecasts suggest that total DAH will likely decrease further in the immediate future (figure 4). The expected DAH is forecasted to decline to $36.2 billion in 2030.

Our sensitivity analyses suggest that our estimate for 2025 could range from $36.6 billion in a pessimistic scenario to $44.0 billion in an optimistic scenario based on changes in US cuts. Similarly, in the next five years, total DAH is expected to reach $41.7 billion in 2030 under a positive scenario for US spending contribution and $34.4 billion if under a negative scenario for US spending contribution. Although these forecasts are made with great uncertainty, first initial projections are valuable for policy, planning, and advocacy. The expected and future availability of DAH estimates suggest that low-income and middle-income countries will increasingly have to rely on alternative sources of funding for their health sectors.

## Discussion

This study generated historical estimates of DAH from 1990 to 2024, preliminary estimates of DAH in 2025, and forecasted DAH estimates from 2026 to 2030. To our knowledge, this is the first extensive study to report DAH estimates that comprehensively reflect recently announced funding cuts by major donors. We found that global DAH is set to decrease by 22% ($11.2 billion) between 2024 and 2025, followed by an additional decrease of 6% ($2.2 billion) between 2025 and 2030. Our preliminary estimate of total DAH in 2025 suggests that global DAH will decline to levels ($38.4 billion) last seen since the middle of the MDG era, more than 15 years ago.

Much of this previously unanticipated decline is driven by funding cuts announced by the USA, which is expected to decrease its DAH disbursement by 67% in 2025 ($9.5 billion) compared to 2024 ($14.2 billion; appendix p 30). Although the US government was the largest single source of development assistance in 2024 and averaged $16.4 billion in DAH disbursement between 2010 and 2024, our preliminary estimate of total DAH from the US government (inclusive of transfers to multilateral aid agencies and administrative costs) will be $4.8 billion in 2025. This assessment is based on the proposed US administration budget and other statements. However, political negotiation between the administration and Congress on the budget may change the outcome substantially, as the US government budgeting process is exceedingly complex, with the final outcome dependent on several evolving factors that are hard to predict beforehand. For instance, budget recissions amounting to a $9.4 billion reduction in already appropriated funds for previous years (2025 and earlier) were proposed on May 28, 2025, but the outcome of those proposed cuts needs to be affirmed within 45 days.^27^ The US fiscal year 2026 budget (proposed on May 2, 2025) includes much larger cuts, but it has a long process of being affirmed by both US houses of congress.^23^ More importantly, the reorganization of US global health programs (e.g., the President’s Emergency Plan for AIDS Relief and the President’s Malaria Initiative) have predated and outpaced the proposed budget recissions; the outcome of that reorganisation and other impound proposals are yet unknown. Therefore, to the best of our ability, the estimates reported in this Article reflect both the proposed budget cuts and our understanding of the closing of US global health programs and offices, as well as changes in US contributions to global programs (e.g., The Global Fund and Gavi) and UN agencies (e.g., WHO and UNAIDS). These estimates have been generated based on all available information as of June 25, 2025.

Declines in DAH disbursement are also expected from other major donor countries including the UK, France, and Germany. For instance, the UK government has announced that starting in 2026 and 2027, they will cut Official Development Assistance funding from 0.5% to 0.3% of GNI.^28^ Similarly, Germany, Switzerland, and Belgium have announced plans to reallocate their aid budgets.^29^ Some donor governments have cited a need to reallocate some of these Official Development Assistance funds towards defense to prioritise domestic security, given geopolitical tensions raised by the Russia-Ukraine war. Other donor governments have cited a need to use some of these funds internally to resettle migrants who continue to flow into their countries.^30,31^

The effect of funding cuts on health outcomes is expected to be substantial. In countries such as Somalia, DR Congo, and Malawi, most funding for their health systems comes from development assistance.^32^ The countries are expected to have cuts to DAH that reflect 15% ($512.8 million decline in the Democratic Republic of the Congo) to 22% ($224.9 million decline in Somalia) of their total health spending. Furthermore, DAH from the USA has historically predominantly targeted specific Global Burden of Diseases, Injuries, and Risk Factors Study super-regions: sub-Saharan Africa ($20.1 billion in 2022), south Asia ($5.1 billion in 2022), and southeast Asia, east Asia, and Oceania ($4.6 billion in 2022) along with specific health areas: HIV/AIDS, systems-wide approaches and health sector strengthening, and neonatal and child health. The health areas make up to 20% of total health spending in the listed regions. Some estimates suggest that there will be tens of millions of lives lost between now and 2040 if the funding cuts persist, whereas other estimates suggest that there have already been tens of thousands of lives lost due to the cuts.^33,34^ Although quantifying the immediate and long-term effect of these cuts is challenging and leads to varied estimates depending on assumptions and model choice, all estimates suggest that the loss of life to preventable deaths will be substantial. Future research should focus on estimating health care spending possibilities relative to adjusted health outcomes. Controlling for key non-health system factors, some of which have the largest impacts on health outcomes (e.g., income and water and sanitation), is crucial. These numbers are important to signify the magnitude of the likely impacts and highlight where funds and support are needed most urgently.

Given the expected cuts in DAH, the extent to which domestic resources might be used to fill the gap is one of the most pressing questions in global public health. An initial response from some low-income and middle-income countries, primarily in Africa, has been to rally together to commit to increasing domestic government funding for the health sector across the continent.^35–38^ To lead this charge, the Africa Centres for Disease Control and Prevention launched a policy document titled “Africa’s Health Financing in a New Era” in April, 2025.^11^ The document guides African governments towards a new paradigm in health financing on the continent, urging governments to not only increase the share of the national budgets allocated to health, but also to explore alternative ways of mobilising resources for the sector – for example, through solidarity levies on mobile services and alcohol, blending financing that leverages both public and private funding, and exploring opportunities to leverage remittances to the continent. Separately, Nigeria has already responded to announced cuts by approving a $200 million increase in domestic health spending, although our estimates suggest the total cut in DAH received by Nigeria is likely to be near $597 million.^39^ The ministries of health of Botswana, Cameroon, and Kenya have likewise pledged to repurpose domestic resources to fund domestic HIV care.^40^ If and how these commitments will be realised (and what other countries will do in response to these cuts) remains unknown but of utmost importance. Past research showed that increases in DAH have been met with reductions in government spending on health, although this research has not found that governments increase government health spending when DAH is cut.^41,42^ Specifically, previous research found that a $1 year-over-year increase in DAH disbursed through the government results in a $0.62 decrease in a domestic government’s own revenue-backed spending on health, whereas a $1 year-over-year decrease in DAH disbursed through government did not have a statistically significant effect on a government’s own spending on health. Still, the cuts assessed in this previous research pale in comparison to the cuts expected in 2025, so government responses might differ as well. Recent economic analyses suggest that countries are still grappling with debt stemming from rapid increases in debt servicing costs due to large increases in interest rates in the recent past^43–46^. Furthermore, government tax revenues in many low-income countries are less than 15% tax to GDP ratio, an estimated level necessary for governments to have enough fiscal space to allocate resources for health and other social sectors.^43,47,48^ In addition, countries also vary widely in health system and health financing context as well as human resource capacity to adapt and respond to the current aid cuts. For instance, South Africa is reported to provide up to 70% of its spending on HIV/AIDS and is known to have a relatively stable health system, while countries such as Central Africa Republic and Somalia have reportedly very limited capacities both in financial and human resource terms.^49–51^ All these factors make the domestic response complex to predict.

Beyond the domestic government response, there are also concerns about responses by other state-level donors. To date, donor government DAH cuts have been made independently, with some countries announcing cuts prior to the US announcement. Historically, the USA has incentivised contributions to agencies such as The Global Fund by offering a matching pledge. There are now concerns that in the absence of US leadership, other governments will renege on their commitments to these agencies, or will pull bilateral funding to countries in accordance with the USA’s cuts to such agencies, which will have a multiplicative impact on the cuts announced by the USA.^52,53^ Recent forecasting work likewise found that a 50% decrease in US DAH might result in a 19.8% larger effect on total DAH levels, since major donors have historically coordinated their magnitude of aid.^52^ Another possible response to the cuts from other donors is to increase their own DAH provision. Although this type of response has not been as pervasive, we note that China has recently committed to providing $500 million and Germany EUR 2 million of annual support in additional funding to WHO in response to the budget shortfalls resulting from the US cuts.^54,55^

Another ramification of the changed landscape of DAH could potentially be seen in the changing landscape of global health leaders and workers. Based upon the provision of substantial amounts of aid, the global health workforce and leadership have historically been dominated by American citizens. Given the refocusing of aid in the USA, it is likely that the face of global health leadership will also change in line with the ongoing change in the locus of power in global health.^53,56–59^ Although the COVID-19 pandemic heralded a dramatic increase in year-on-year DAH provided, trends in DAH provided up to 2024 suggested a recalibration of levels of aid comparable to the pre-pandemic years. This pattern in DAH trend suggests that despite calls to maintain the increases in funding for preparedness, the health-related response to the COVID-19 pandemic followed the so-called panic and neglect approach that has historically characterised DAH funding during emergencies. The aid cuts announced recently serve as the latest shake-up in the global aid architecture. The existing aid system has been criticised for being fragmented and inefficient, with an increasing number of donor agencies but smaller amounts of funding.^57–59^ As others have suggested, in entering a period of austerity for DAH, stakeholders might now be more compelled to address the inefficiencies that have limited the effectiveness of aid as countries aim to do more with less.^53,56^ Furthermore, this may present the opportunity other non-DAC donors such as the BRICs and middle-eastern donors seek to boost their influence in DAH. Such changes in the major sources will have implications for the types of health activities that are funded and the approach for conducting aid business. This is because these non-DAC donors, particularly China, tend to invest more in infrastructure and prevention activities and generally follow a mutual benefit, south-south cooperation and non-interfering in recipient country affairs approach in aid management. One factor that could shape the aid architecture in the coming years is the growing influence and use of artificial intelligence (AI) in all areas of life. While the full benefits and threats presented by the use of AI in DAH may be currently underexplored, there are many potential use cases through digital health that suggest that if harnessed properly it could be used to make significant inroads on the path to ensuring that aid is efficiently used and has transformative impact.

### Limitations

This study has several important limitations. First, to produce 2025 estimates, we assumed that money already committed, and as yet unpaid, will still be disbursed. While using commitment data is the best that can be done based on the recency of the cuts, we note that there may be gaps between what is committed and what is disbursed. Importantly, many of the proposed cuts are also still going through various legislative processes before they can be appropriated. So, while we have used the most up-to-date information available as of this writing, we acknowledge that there is a possibility that final cuts may deviate from our estimates. Additionally, we made some assumptions in order to generate our near-term forecasts of the trajectory of DAH, including that DAH will grow at the same rate as GNI for some countries. While we believe these assumptions are reasonable, we acknowledge that the future is uncertain, and those estimates too may deviate from the final amounts provided in DAH. Data gaps resulting from the lack of official reporting of DAH provision by donors such as China, India, and South Africa that support south and south cooperation also increase the uncertainty around the estimate. We have included estimates of DAH from China that were generated internally to address some of this data gap but acknowledge that we still have some donor countries whose contributions are not fully accounted for in this analysis. Furthermore, our DAH estimates rest on the assumption that reductions in DAH are proportionate to the cuts in ODA, and policy choices announced in 2024 and 2025 are maintained through 2030 in real terms. Lastly, we acknowledge that this is an evolving situation with cuts and responses being announced each day. We have thus included a sensitivity analysis that varies the expected level of cut from the USA to provide a range of the expected DAH. We focused on varying the US cuts because they are the most drastic and immediate but acknowledge that the cuts announced by other donors may also change as well although those are not varied in our sensitivity analysis.

## Conclusion

The year 2025 has ushered in a significant shock in the development assistance community and global aid architecture as the USA and other donor countries refocus their foreign aid priorities. This has led to large declines in DAH disbursed, and the amount of DAH provided has retreated to levels not seen in the last 15 years. Urgently, more resources and more efficient delivery systems are needed to maintain essential services to the world’s poorest populations. Lingering questions of how many domestic resources can be raised to fill gaps, if any donors can contribute more, and to what degree health systems can do more with less are critical. How these questions are answered will have stark ramifications for global health.

## Supplementary Material

Supplementary appendix

## Figures and Tables

**Table 1.** Flows of Development Assistance for Health by Source and Disbursing Entity, 2025

*Source/Disbursing Entity*	Australia	CEPI	Canada	Development banks	European Commission	France	Gates Foundation	Gavi	Germany	Global Fund	Japan	NGOs & Foundations	Other bilateral aid agencies	UN agencies	United Kingdom	United States	WHO	*Total*
**Australia**	216	17		12				65		112		140		97			54	**713**
**Canada**		11	191	101				54		307		380		168			71	**1,283**
**France**		2		169	89	263		71		278		133		66			44	**1,115**
**Gates Foundation**		21		0			4,417	238		315		3		47			493	**5,534**
**Germany**		3		105	189			107	640	397		217		393			287	**2,338**
**Japan**		43		202				11		262	657	28		134			73	**1,410**
**Other governments**		45		421	435			291		534		1,248	1,601	1,272			863	**6,710**
**Other sources**		0		3,924				371		0		0		1,785			898	**6,978**
**Private philanthropy**		49		0				6		91		4,857		957			425	**6,385**
**United Kingdom**		29		145	19			194		2		249		108	322		157	**1,225**
**United States**		27		127				0		800		1,224		15		2,564	0	**4,757**
***Total***	**216**	**247**	**191**	**5,206**	**732**	**263**	**4,417**	**1,408**	**640**	**3,098**	**657**	**8,479**	**1,601**	**5,042**	**322**	**2,564**	**3,365**	**38,448**

* Values represent total DAHfor the source-channel flow in 2025 (millions, 2023 USD)

Values represent total DAH for the source-disbursing entity combination and highlight the flow of resources from the source to the final disbursing entity in 2025. Reported in millions 22 of 2023 real USD. CEPI=Coalition for Epidemic Preparedness Innovations. NGOs=non-governmental organisations.

## Data Availability

Data used for this study were extracted from publicly available sources that are listed in the appendix (sections 3 & 4). Further details are available on the Global Health Data Exchange website (https://ghdx.healthdata.org/series/financing-global-health-fgh).

## Research in context

### Evidence before this study

There are four organisations that track development assistance for health (DAH). The Organisation of Economic Cooperation and Development’s (OECD) Development Assistance Committee (DAC) provides the Creditor Reporting System (CRS) database in which members of the OECD DAC report official development assistance disbursements. Only more recent years of the CRS database have comprehensive data that report all disbursements by donors and the CRS does not include NGOs, a range of private foundations and development banks, or some disbursements from UN agencies. The International Aid Transparency Initiative (IATI) is a voluntary repository for development-focused institutions to report their aid disbursements and activities to help improve aid transparency. IATI reports are typically current or very recent, although not exhaustive. As part of its Global Health Expenditure Database, WHO reports DAH received in-country as reported by recipient countries but excludes administrative costs and some off-budget DAH, which is provided to NGOs rather than the government. The DAH database produced by IHME is the most comprehensive, providing estimates for 1990 by source, disbursing entity, and health area, and includes grants, loans, and administrative costs. IHME is also the only entity providing estimates of expected DAH in the future (through 2050). For all four databases, the latest DAH estimates were all generated prior to substantial DAH cuts announced by the USA (January 2025) and the UK (25 February 2025).

### Added value of this study

To our knowledge, this was the first study to report comprehensive estimates of development assistance for health since major reductions were announced by major donors in late 2024 and early 2025. We provided retrospective estimates of disbursed DAH from 1990 to 2024, preliminary DAH estimates for 2025 based on changes in countries’ development assistance budgets or public statements from government officials declaring cuts. We used additional information about expected future disbursements to generate one plausible scenario of future estimates of DAH through 2030. Retrospective DAH estimates were disaggregated by the source of DAH, disbursing entity, health focus area, and recipient country, while future disbursements were estimated by source and recipient. Additionally, we made improvements to the estimates such as expanding the donors covered in the database and leveraging additional project-level data to improve our disaggregation of health areas.

### Implications of all the available evidence

All available evidence suggests that the future availability of DAH will be contracted. For low-income countries, for which DAH is a major source of health sector funding, these cuts require an urgent response. Immediate strategising about how the health sector will be funded is critical, as all else being equal, recipient governments are very likely to be unable to cover most of the gap left by global donors. As we enter an era of global health austerity, it is more imperative than ever that we seek to augment DAH contributions and increase efficiency in their use.
