# Supplementary material for: Tracking development assistance for health, 1990–2030: historical trends, recent cuts, and outlook
Source: Lancet. Author manuscript; Available in PMC 2025 Sep 17. (PMC12439094; doi:10.1016/S0140-6736(25)01240-1)
Supplement: Supplementary appendix [file NIHMS2107934-supplement-Supplementary_appendix.pdf]

STANDALONE METHODS ANNEX

Financing Global Health 2024: Tracking development assistance for health, 1990-2030: historical trends, recent cuts, and outlook

Version: June 6, 2025

This appendix provides further methodological detail for Financing Global Health 2024: Tracking development assistance for health, 1990-2030: historical trends, recent cuts, and outlook

Portions of this appendix have been reproduced or adapted from previous Global Burden of Disease Health Financing Collaborator Network publications.[1,2] References are provided for reproduced sections.

---

1) Global Burden of Disease 2020 Health Financing Collaborator Network. **Tracking development assistance for health and for COVID-19: a review of development assistance, government, out-of-pocket, and other private spending on health for 204 countries and territories, 1990–2050.** *The Lancet.* 22 September 2021. doi: 10.1016/S0140-6736(21)01258-7.

2) Global Burden of Disease 2021 Health Financing Collaborator Network. **Global investments in pandemic preparedness and COVID-19: tracking development assistance and domestic spending on health between 1990 and 2026.** *The Lancet Global Health.* 24 January 2023. doi: 10.1016/S2214-109X(23)00007-4.

Table of Contents

SECTION 1. METHODS OVERVIEW

S1.1 Overview....................Page 7

S1.2 Estimating development assistance for health: 1990–2023....................Page 7

SECTION 2. TRACKING TOTAL HEALTH SPENDING AND ITS COMPONENTS

S2.1 Overview....................Page 13

S2.2 Statistical model to fill missingness in health expenditure variables....................Page 23

S2.3 Currency exchange and deflation....................Page 25

SECTION 3. TRACKING DEVELOPMENT ASSISTANCE FOR HEALTH

S3.1 Overview....................Page 28

S3.2 Tracking development assistance for health from bilateral aid agencies and the European Commission....................Page 49

S3.3 Tracking development assistance for health from the development banks....................Page 60

S3.4 Tracking contributions from the Global Fund and Gavi....................Page 66

S3.5 Tracking expenditure by United Nations Agencies active in the health domain....Page 69

S3.6 Tracking development assistance for health from private foundations....................Page 71

S3.7 Tracking non-governmental organizations....................Page 73

S3.8 Tracking development assistance for health from China....................Page 77

S3.9 Calculating the technical assistance and program support component of development assistance for health from loan-and grant-making channels of assistance....................Page 77

S3.10 Regular development assistance for health references....................Page 88

S3.11 Estimating development assistance for health in 2025....................Page 98

SECTION 4. FORECASTING HEALTH SPENDING FROM 2023 THROUGH 2030

S4.0 Projecting Development Assistance from 2026 to 2030....................Page 108

S4.1 Data....................Page 109

S4.1.1 Summary of data sources

S4.1.2 World Health Organization's Global Health Expenditure Database

S4.1.3 WB WDI, IMF WEO, PWT, and Maddison

S4.1.4 Institute for Health Metrics and Evaluation's Development Assistance for Health Database

S4.1.5 Institute for Health Metrics and Evaluation's Global Burden of Disease Study 2021

S4.2 Covariates..Page 112

S4.2.1 List of forecasted variables

S4.2.2 Covariates used for forecasting

S4.3 Ensemble modeling...Page 114

S4.3.1 Sub-model setup

S4.3.2 Covariates

S4.3.3 Convergence to global growth rates

S4.3.4 Specifications

S4.4 Inclusion and exclusion criteria...Page 117

S4.5 Creating the forecasts...Page 118

S4.5.1 Ranking sub-models

S4.5.2 Uncertainty estimation

S4.6 Ad-hoc draws correlation...Page 120

S4.6.1 Motivation

S4.6.2 Bivariate correlated distributions

S4.7 Future health scenarios..Page 120

S4.7.1 Long-term growth regressions

S4.7.2 Forecasting greater and lesser scenarios

S4.7.3 Uncertainty estimation

S4.8 Package and architecture...Page 123

S4.8.1 Architecture

S4.8.2 Template model builder

S4.9 Tables and figures..Page 125

Abbreviations

ADB	Asian Development Bank
AfDB	African Development Bank
BMGF	Bill & Melinda Gates Foundation
CRS	Creditor Reporting System
DAC	Development Assistance Committee
DAH	Development assistance for health
EC	European Commission
GDP	Gross domestic product
Global Fund	Global Fund to fight AIDS, Tuberculosis and Malaria
GGHE	General Government health expenditure
GHED	Global Health Expenditure Database
GHES	Government health spending (Aggregate of "Social insurance contributions" and "Transfers from government domestic revenue (allocated to health purposes)," as defined in GHED)
HSS	Health system strengthening
IBRD	International Bank for Reconstruction and Development
IDA	International Development Association
IDB	Inter-American Development Bank
LCU	Local currency units
NASA	National AIDS Spending Assessments
NGO	Non-governmental organization
NHA	National Health Account
NPISH	Non-profit institutions serving households
ODA	Official development assistance
OECD	Organisation for Economic Co-operation and Development
OOP	Out-of-pocket health spending (defined in GHED: "Other revenues from households n.e.c")
PAHO	Pan American Regional Office for WHO
PI	Private insurance
PPP	Prepaid private health spending (Aggregate of "Voluntary prepayment," "Other revenues from corporations n.e.c.," "Other revenues from NPISH n.e.c.," and "Compulsory prepayment (Other, and unspecified, than FS.3)," as defined in GHED)

SHA 2011	System of Health Accounts 2011
SWAps	Sector-wide approaches
TB	Tuberculosis
THE	Total health spending (defined in GHED: “Current health expenditure by revenues of health care financing schemes”)
UNICEF	United Nations Children’s Fund
UNAIDS	Joint United Nations Programme on HIV/AIDS
UNFPA	United Nations Population Fund
USD	US dollars
VolAg	Report of Voluntary Agencies
WHO	World Health Organization

SECTION 1. METHODS OVERVIEW

S1.1 Overview

The methods presented here summarize the various components of the estimation process. "Health spending" is defined as money spent on services, supplies, and basic infrastructure to deliver health care, using the same definition employed by the System of Health Accounts 2011 and the WHO Global Health Expenditure Database (GHED).[1,2] Health "financing" and "funding" are used interchangeably to refer to the source, as opposed to the utilization, of financial resources. "Economic development" refers to GDP per capita.

We estimated health spending from four main sources – government, out-of-pocket, prepaid private, and development assistance for health (DAH) – for 204 countries and territories shown in TS1.1. For brevity, "countries and territories" are referred to only as "countries," all of which are categorized into four World Bank income groups (high-, upper-middle-, lower-middle-, and low-income) and seven Global Burden of Disease (GBD) super-regions (Central Europe, Eastern Europe, and Central Asia; GBD high income; Latin America and Caribbean; North Africa and Middle East; South Asia; Southeast Asia, East Asia, and Oceania; and sub-Saharan Africa). Data tracking government, out-of-pocket, and prepaid private health spending, which together constitute total domestic health spending, were available from 1995 through 2022. Government health spending includes social health insurance and mandated private health insurance, as well as government public health programs. Out-of-pocket health spending includes health care spending paid by the patient or his or her household, excluding insurance premiums paid in advance of care. Prepaid private health spending includes voluntary private insurance and non-governmental agency spending on health.

Estimates of DAH, defined as the financial and in-kind contributions from major development agencies to low- and middle-income countries for the purpose of maintaining or improving population health, were generated from 1990 through 2025, although transfers to recipient countries can only be estimated until 2023. In other words, the total amount of DAH, by source, is estimated through 2025, but is not allocated by recipient country for 2024 or 2025. The sum of domestic health spending and DAH, net of the administrative costs needed to run the development agencies, form the envelope of total health spending for each country and year.

Domestic health spending from each of the three sources was projected for each country from 2023 to 2050, and DAH by recipient was projected from 2024 to 2050, by modeling rates of change across time. These models incorporate country-specific time trends that attenuate across time and converge to the global average, consider a broad set of covariates and time-series modeling techniques, and propagate four types of uncertainty – model, data, parameter, and fundamental uncertainty.

S1.2 Estimating development assistance for health: 1990–2025

To track DAH, we relied on revenue data in financial statements, annual reports, budget documents, and project disbursement records reported by international development agencies such as the Organisation for Economic Co-operation and Development (OECD) Creditor Reporting System, the World Bank, the Global Fund, major philanthropic entities like the Gates Foundation and the David and Lucile Packard Foundation.[11-20, 33-34] We used budget and commitment data to generate estimates for recent years for which

disbursement data were not yet available; for most channels, data extend to 2023 or 2024 so that at most two years of estimation were needed to complete the estimates through 2024. This year, we additionally estimated DAH through 2025 by relying on publicly announced statements from donors about their future official development assistance and global health spending, as discussed in S3.11

Our DAH estimates tracked disbursements from the originating donor (called the source); to the development agency responsible for disbursing the funds to the recipient country (called the channel); and to the recipient country. We used revenue and disbursement data to count the transfers between development agencies only once to limit double counting. In addition to reporting the source, channel, and recipient of DAH, we disaggregated spending into nine major health focus areas (as well as more detailed program areas) by searching project titles and descriptions for keywords, as well as using other indicators in the data. Health focus areas included HIV/AIDS; malaria; tuberculosis; reproductive and maternal health; newborn and child health; non-communicable diseases; other infectious diseases; sector-wide approaches and health system strengthening; and other. The “Other” category captured projects such as general support for a conference on the Sustainable Development Goals that were not allocated to any of the eight specific health focus areas; remaining funds for which no project descriptions were available were classified as “unallocable.” The list of keywords used to isolate relevant health projects for each of the health focus areas are included in the supplemental appendix Table S3.4.

While the majority of the methods used for tracking DAH have been described previously, the current estimates of DAH incorporated changes in methodology such as leveraging 990 form scraping to enable more direct calculation of DAH from NGOs rather than our previous sampling based method, and a similar move to acquiring foundations data from 990 forms and foundation websites.[21-25, 32-33] Additionally, we have utilized data collected from the International Aid Transparency Initiative (IATI) and AidData to obtain more detailed project-level information for some of the channels we track.[35,36] For all DAH tracking, we include funds that were transferred through major development agencies included in our research, as well as private foundation and non-governmental agencies for which we have data. This definition of DAH excludes spending on basic bench science that may have downstream effects on the development of new drugs.

Spending type	Main sources of data	Data type
Development assistance	• Organisation for Economic Co-operation and Development (OECD) Creditor Reporting System<br>• The World Bank<br>• The Global Fund<br>• Gates Foundation,<br>• Propublica<br>• International Aid Transparency Initiative	• Financial statements<br>• Annual reports<br>• Budget documents<br>• Project disbursement records<br>• Tax forms

Total domestic health spending (government spending, out-of-pocket spending, prepaid private spending)	• World Bank<br>• International Monetary Fund (IMF)<br>• United Nations<br>• Penn World Tables<br>• The Maddison Project<br>• WHO's Global Health Expenditure Database (GHED)	• Gross domestic product (GDP) per capita<br>• Government domestic revenue transfers allocated for:<br>○ Health<br>○ Compulsory prepayment<br>○ Voluntary prepayment<br>○ Social insurance contributions<br>○ Other domestic revenue from households, corporations, and nonprofit institutions serving households

References

1 WHO | Global Health Expenditure Database. WHO. http://www.who.int/health-accounts/ghed/en/ (accessed January 11, 2025).

2 OECD, Eurostat and World Health Organization. A System of Health Accounts 2011: Revised edition. Paris: OECD Publishing, 2017 https://read.oecd-ilibrary.org/social-issues-migration-health/a-system-of-health-accounts-2011_9789264270985-en (accessed March 7, 2019).

3 The Database | Penn World Table version 10.01. Groningen Growth and Development Centre: Faculty of Economics and Business. https://www.rug.nl/ggdc/productivity/pwt/ (accessed Feb 7, 2025).

4 DataBank | The World Bank. The World Bank. http://databank.worldbank.org/data/home.aspx (accessed Feb 7, 2025).

5 International Monetary Fund. World Economic Outlook, April 2025: A Critical Juncture and Policy Shifts, 2025. https://www.imf.org/en/Publications/WEO/Issues/2025/04/22/world-economic-outlook-april-2025 (accessed April 25, 2025).

6 Maddison Project Database 2020. Groningen Growth and Development Centre: Faculty of Economics and Business. https://www.rug.nl/ggdc/historicaldevelopment/maddison/releases/maddison-project-database-20 (accessed Feb 7, 2025).

7 National Accounts Main Aggregates Database. United Nations Statistics Division - National Health Accounts. https://unstats.un.org/unsd/snaama/ (accessed Feb 7, 2025).

8 James SL, Gubbins P, Murray CJ, Gakidou E. Developing a comprehensive time series of GDP per capita for 210 countries from 1950 to 2015. *Population Health Metrics* 2012; **10**: 12.

9 Global Health Expenditure Database. World Health Organization. http://apps.who.int/nha/database (accessed Jan 11, 2025).

10 GBD 2015 Risk Factors Collaborators. Global, regional, and national comparative risk assessment of 79 behavioural, environmental and occupational, and metabolic risks or clusters of risks, 1990–2015: a systematic analysis for the Global Burden of Disease Study 2015. The Lancet 2016; 388: 1659–724.

11 Grants. https://data.theglobalfund.org/grants (accessed May 23, 2025).

12 OECD Data Explorer. https://data-explorer.oecd.org/ (accessed May 23, 2025).

13 Malaria Operational Plans (MOPs) | PMI. https://www.pmi.gov/resources/malaria-operational-plans-mops/ (accessed Jan 20, 2024).

14 Key figures: donor contributions & pledges. https://www.gavi.org/investing-gavi/funding/donor-profiles (accessed May 23, 2025).

15 Trustees reports and financial statements - International Finance Facility for Immunisation. https://iffim.org/investor-centre/trustee-reports-financial-statements (accessed Jan 20, 2024).

16 Financial reports. https://www.gavi.org/news-resources/document-library/financial-reports (accessed May 23, 2025).

17 Gavi transparency portal and IATI. https://www.gavi.org/our-alliance/operating-model/gavi-transparency-portal-and-iati (accessed May 23, 2025).

18 UNAIDS. PCB Archive. http://www.unaids.org/en/aboutunaids/unaidsprogrammecoordinatingboard/pcbmeetingarchive (accessed May 23, 2025).

19 Gates Foundation Financial statements https://www.gatesfoundation.org/about/financials/annual-reports (accessed May 23, 2025).

20 GB | Governing Body Documentation. http://apps.who.int/gb/ (accessed May 23, 2025)

21 Ravishankar N, Gubbins P, Cooley RJ, et al. Financing of global health: tracking development assistance for health from 1990 to 2007. The Lancet 2009; 373: 2113–24.

22 Leach-Kemon K, Chou DP, Schneider MT, et al. The Global Financial Crisis Has Led To A Slowdown In Growth Of Funding To Improve Health In Many Developing Countries. Health Affairs 2012; 31: 228–35.

23 Dieleman JL, Graves C, Johnson E, et al. Sources and Focus of Health Development Assistance, 1990–2014. JAMA 2015; 313: 2359–68.

24 Dieleman JL, Graves CM, Templin T, et al. Global health development assistance remained steady in 2013 but did not align with recipients' disease burden. Health Aff (Millwood) 2014; 33: 878–86.

25 Dieleman JL, Templin T, Sadat N, et al. National spending on health by source for 184 countries between 2013 and 2040. The Lancet 2016; 387: 2521–35.

26 Micah AE, Zhao Y, Chen CS, et al. Tracking development assistance for health from China, 2000–2018. BMJ Global Health under review.

27 Das Gupta P. Standardization and Decompostion of Rates: A User's Manual. U.S. Bureau of the Census, 1993 https://www.census.gov/content/dam/Census/library/publications/1993/demo/p23-186.pdf (accessed Dec 21, 2018).

28 World Bank Country and Lending Groups – World Bank Data Help Desk. The World Bank. https://datahelpdesk.worldbank.org/knowledgebase/articles/906519-world-bank-country-and-lending-groups (accessed Oct 15, 2024).

29 GBD 2019 Demographics Collaborators. Global age-sex-specific fertility, mortality, healthy life expectancy (HALE), and population estimates in 204 countries and territories, 1950–2019: a comprehensive demographic analysis for the Global Burden of Disease Study 2019. The Lancet. 17 October 2020. doi:10.1016/S0140-6736(20)30977-6.

30 Global Economic Prospects, January 2025. The World Bank. The World Bank. https://www.worldbank.org/en/publication/global-economic-prospects (accessed Feb 7, 2025).

31 World Economic Situation and Prospects Report 2025. United Nations Department of Economic and Social Affairs. https://www.un.org/development/desa/dpad/publication/world-economic-situation-and-prospects-2025/ (accessed Feb 7, 2025).

32 International Monetary Fund. World Economic Outlook Update, January 2025: Global Growth: Divergent and Uncertain. International Monetary Fund, 2025. https://www.imf.org/en/Publications/WEO/Issues/2025/01/17/world-economic-outlook-update-january-2025 (accessed Feb 7, 2025).

33 Propublica Nonprofit Explorer. https://projects.propublica.org/nonprofits/ (accessed March 2024).

34 David and Lucile Packard Foundation. https://www.packard.org/grantees/search-our-grants/ (Accessed March 2024).

35 International Aid Transparency Initiative. https://iatistandard.org/en/ (accessed May 9, 2025).

36 AidData. 2023. Global Chinese Development Finance Dataset, Version 3.0. Retrieved from https://www.aiddata.org/data/aiddatas-global-chinese-development-finance-dataset-version-3-0 (accessed Nov 19, 2024).

SECTION 2. TRACKING TOTAL HEALTH SPENDING AND ITS COMPONENTS

List of tables and figures

Table S2.1 Rules for assigning weight values to metadata

Table S2.2 Countries and income groups, number of assigned weights

Figure S2.1 Comparison of WHO GHED extracted and IHME ST-GPR THE in per capita space, 2000–2022

Figure S2.2 Comparison of WHO GHED extracted and IHME ST-GPR THE in per GDP space, 2000–2022

Figure S2.3 Currency conversion process diagram

S 2.1 Overview of GHED data cleaning process

We used Global Health Expenditure Database (GHED) data from the World Health Organization (WHO) to generate our estimates.1 From the GHED, we extracted "Current health expenditure by revenues of health care financing schemes" for total health expenditure (THE), "Other revenues from households n.e.c." for out-of-pocket (OOP), "Gross Domestic Product" for GDP. We summed "Social insurance contributions" and "Transfers from government domestic revenue (allocated to health purposes)" for government health spending (GHE). We summed "Voluntary prepayment," "Other revenues from corporations n.e.c.," "Compulsory prepayment (other, and unspecified, than FS.3)," and "Other revenues from NPISH n.e.c." for pre-paid private (PPP).

To ensure we used the best possible data from the GHED, we evaluated the metadata also provided by GHED to establish the reliability of the data. To do so, we downloaded the metadata from the GHED website for each data point for the five indicators. We used the metadata to decide how each given data point should be weighted, from 0 to 5, with 0 meaning drop, and 1 through 5 meaning keep, and treated these weights as inverse variance weights.

To assign the weights, we established guidelines for the metadata that informed how the underlying data points should be weighted. We gave priority to factors such as complete, documented source information and penalized factors such as having been derived or estimated. Table S2.1 below describes the guidelines we created; any metadata that did not meet any of the disqualifying factors were given a value of 5 to reflect highest reliability.

To assign weights to the latest GHED estimates, we trained a linear SVM (support vector machine) model supported by an NLP (natural language processing) pipeline that learned from the weights that were manually assigned (based on the aforementioned guidelines) to metadata downloaded in the year 2017. A linear SVM model using the Sklearn library was chosen since its strengths aligned well with our task and the model performed well overall. The NLP pipeline consisted of a count vectorizer to transform the text metadata matrix into a matrix of token counts that can easily be interpreted by any machine learning algorithm. The count vectorizer also helped us bring in the possibility of simple words as well as combinations of simple words (i.e., word n-grams) to help with predicting the optimum reliability weight for each data point. The final model was tuned to achieve close resemblance for predicted weights when juxtaposed against the manually assigned weights for data points downloaded in the year 2017; and then used to predict weights for data points downloaded in 2025.

We used the four primary metadata variables from the GHED database: data type, method of estimation, comments, and sources. We applied the guidelines to each unique set of metadata across these four variables. For a subset of data points, the metadata indicated that the data point was the sum of other data points. In these cases, if the indicator was a sub-indicator of GHES, PPP, or OOP, we assigned the data point a value of 2 to reflect that even though we could not determine if the sub-components existed, as they are not reported in GHED, we did not feel that being a sum warranted dropping the data. We assigned the summed GHES, PPP, and OOP indicators the lowest value of its sub-indicators. If the summed data point was THE, however, we assigned the data point the lowest value of its sub-components, the summed GHES, PPP, and OOP indicators.

After designating each of the 5,079 unique sets of metadata a value weight, we applied these weights to the underlying data points. In total, we had 38,641 data points, as multiple data points shared the same unique set of metadata. Once the weights were applied to the data, we reassigned all high-income country data points that were a 0 based on the metadata to 3. We made this change to reflect that high-income countries have higher-quality data and thus should not be dropped, but should also not be given the highest weight value. The high-income classification comes from the World Bank.[2] For all countries across all years of data, their relative weighting scores as well as the percentage completeness of underlying cited data can be seen in Table S2.2.

Each component of health expenditure was aggregated for all component-year-country combinations by taking component values (downloaded in current national unit); dividing by GHED reported GDP for the corresponding year-country (also in current national unit) to convert each component into its fraction of GDP; and finally multiplying those fractions by IHME GDP series measured in USD 2023. (Note: though we harvested and cleaned metadata for THE and GDP, we do not model THE [we model GHE, PPP, OOP and then aggregate] or use WHO's GDP data since we have our own methods with multiple sources for determination of our value).

Each component of health expenditure was then converted into three spending metrics for the time period 2000–2022, measured in purchasing power parity (PPP): health spending per capita ($), annualized rate of change in health spending per capita, 2000–2022 (%), and annualized rate of change in health spending, 2000–2022 (%). Then, each component of health expenditure was aggregated and converted into six spending metrics for the time period 2023–2050, measured in PPP: health spending per capita ($), annualized rate of change in health spending per capita, 2023–2050 (%), annualized rate of change in health spending, 2023–2050 (%), Government health spending per capita ($), difference between GHEpc (government health expenditure per capita) reference scenario and greater scenario 1, 2050 ($), and difference between GHEpc reference scenario and greater scenario 2, 2050 ($).

Formula for calculating AROC (Annualized rate of change):

If you apply this growth rate n times (where n is the number of steps from starting to ending year) to the value in the starting year, you will get the value in the ending year. For example:

$$\underset{1990\to 2019}{\text{AROC}} = \left(\frac{value\ in\ 2019}{value\ in\ 1990}\right)^{\frac{1}{2019-1990}} - 1$$

Table S2.1 Rules for assigning weight values to metadata

Data type	Methods of estimation	Sources and/or comments	Weight
Blank			0
Estimated			0
	Derived by applying the sum of the components		Lowest weight of the components
	Interpolated but with additional information		2
	Method description is unclear or provides very little information		0
	Time trend interpolation		1
	Uses data from other countries		0
		Abstract that's not from something documented	1 or method (whichever is bigger)
		Adjusted	0 or method (whichever is bigger)
		Adjusted using a described method	2 or method (whichever is bigger)
		Any suggestion that WHO is unclear or unsure about some aspect of the data point's metadata	0 or method (whichever is bigger)
		Approximation	0 or method (whichever is bigger)
		Assumption	0 or method (whichever is bigger)
		Both blank	0 or method (whichever is bigger)
		Both with no intelligible information	0 or method (whichever is bigger)
		Budget address	1 or method (whichever is smaller)
		Calculation was used to generate the estimate	1 or method (whichever is bigger)
		Consultation/contact (without an additional documented source)	1 or method (whichever is smaller)
		Consultations with additional source, but no specifics and just consult is documented	1 or method (whichever is bigger)
		Currency conversion	1 or method (whichever is bigger)

Data type	Methods of estimation	Sources and/or comments	Weight
		Data delivered/provided/reported by (a non-documented source)	1 or method (whichever is smaller)
		Data provided but not clear by whom, with an additional source if additional source is not documented	1 or method (whichever is smaller)
		Derived	0 or method (whichever is bigger)
		Estimated based on	1 or method (whichever is bigger)
		Estimation	0 or method (whichever is bigger)
		Excludes (if it excludes what we do want)	0 (supersedes method)
		Extrapolated	0 or method (whichever is bigger)
		Forecasted	0 or method (whichever is bigger)
		Government department, no explicit documented source	1 or method (whichever is bigger)
		Government ministry, but no explicit documented source	1 or method (whichever is bigger)
		Includes (if it includes what we don't want)	0 (supersedes method)
		Inferred	0 or method (whichever is bigger)
		Interpolation	0 or method (whichever is bigger)
		Missing (if missing something that should be included)	0 (supersedes method)
		Modified	0 or method (whichever is bigger)
		Modified from something/modified using something	2 or method (whichever is bigger)
		Needs assessment	1 or method (whichever is smaller)
		Needs discussion	1 or method (whichever is smaller)
		Needs validation	1 or method (whichever is smaller)
		Needs verification	1 or method (whichever is smaller)
		Only provides hint of a source	1 or method (whichever is smaller)
		Projected	0 or method (whichever is bigger)
		Provides only a vague term that does not provide adequate information to infer or determine what the source is	0 or method (whichever is bigger)
		Reply	1 or method (whichever is smaller)
		Response	1 or method (whichever is smaller)

Data type	Methods of estimation	Sources and/or comments	Weight
		Speech	1 or method (whichever is smaller)
		Sum of	2 (except for THE, which is lowest weight of components)
		Total of	3 (except for THE, which is lowest weight of components)
		Underestimated	0 (supersedes method)
		Unpublished	1 or method (whichever is smaller)
		Validated figures, but without specifics	2 or method (whichever is bigger)
		Weights	0 or method (whichever is bigger)

Table S2.2 Countries and income groups, number of assigned weights and data completeness

Country	0	1	2	3	4	5	Completeness (%)
Afghanistan	83	74	0	0	0	50	59
Albania	144	0	0	0	0	63	30
Algeria	167	16	0	0	0	24	19
Andorra	0	0	0	138	0	0	66
Angola	163	22	0	0	0	22	21
Antigua and Barbuda	50	0	3	144	0	10	75
Argentina	109	0	59	19	0	20	47
Armenia	87	21	36	1	0	62	57
Australia	0	0	0	207	0	0	100
Austria	0	0	0	207	0	0	100
Azerbaijan	177	7	1	0	0	22	14
Bahamas	0	0	0	207	0	0	100
Bahrain	4	1	1	198	0	3	98
Bangladesh	59	0	0	0	0	148	71
Barbados	25	3	4	171	0	4	87
Belarus	110	11	1	0	4	81	46
Belgium	0	0	0	207	0	0	100
Belize	122	17	41	0	0	27	41
Benin	169	0	0	0	3	35	18
Bhutan	115	0	0	0	0	92	44
Bolivia	165	3	4	0	0	35	20
Bosnia and Herzegovina	109	13	0	0	0	85	47
Botswana	137	0	0	0	0	70	33
Brazil	167	0	4	0	0	36	19
Brunei	0	0	0	207	0	0	100
Bulgaria	118	10	9	0	9	61	42
Burkina Faso	108	0	0	0	0	99	47
Burundi	129	3	0	0	0	75	37
Cabo Verde	101	12	0	0	3	91	51
Cambodia	117	19	4	0	0	67	43
Cameroon	111	3	1	0	0	92	46
Canada	0	0	0	207	0	0	100
Central African Republic	48	0	0	0	0	159	76
Chad	118	40	1	0	1	47	42
Chile	80	0	0	99	0	28	61
China	12	2	6	0	0	187	94
Colombia	88	89	0	0	0	30	57
Comoros	45	41	32	0	1	88	78
Congo	163	6	0	0	0	38	21

Cook Islands	105	18	1	0	0	83	49
Costa Rica	184	0	1	0	0	22	11
Croatia	35	8	16	126	13	9	83
Cuba	113	0	22	0	0	72	45
Cyprus	0	0	0	207	0	0	100
Czechia	42	0	0	153	0	12	79
Côte d’Ivoire	80	0	10	0	1	116	61
Democratic Republic of the Congo	111	0	0	0	0	96	46
Denmark	0	0	0	207	0	0	100
Djibouti	133	0	12	0	4	58	35
Dominica	149	0	27	0	0	31	28
Dominican Republic	158	2	4	1	0	42	23
Ecuador	45	121	18	0	0	23	78
Egypt	72	80	2	0	3	50	65
El Salvador	160	0	0	0	0	47	22
Equatorial Guinea	91	14	1	64	0	14	44
Eritrea	140	1	0	0	0	66	32
Estonia	6	0	3	153	0	45	97
Eswatini	168	8	0	0	0	31	18
Ethiopia	134	30	0	0	0	43	35
Fiji	83	4	2	0	0	118	59
Finland	0	0	0	207	0	0	100
France	0	0	0	207	0	0	100
Gabon	124	0	0	0	0	83	40
Gambia	128	22	2	0	0	55	38
Georgia	58	29	33	0	0	87	71
Germany	0	0	0	207	0	0	100
Ghana	154	0	12	0	0	41	25
Greece	0	0	0	207	0	0	100
Grenada	138	5	21	0	0	43	33
Guatemala	143	0	33	0	0	31	30
Guinea	107	7	0	0	0	93	48
Guinea-Bissau	159	1	0	0	3	44	23
Guyana	121	0	31	9	17	29	41
Haiti	170	2	12	0	0	23	17
Honduras	101	0	33	0	2	71	51
Hungary	15	0	0	126	0	66	92
Iceland	0	0	0	207	0	0	100
India	137	1	0	0	0	69	33
Indonesia	95	28	0	0	10	74	54
Iran	102	64	15	0	0	26	50

Iraq	115	47	12	0	2	31	44
Ireland	0	0	0	207	0	0	100
Israel	0	0	0	207	0	0	100
Italy	0	0	0	207	0	0	100
Jamaica	123	5	29	0	0	50	40
Japan	0	0	0	207	0	0	100
Jordan	71	83	0	0	8	45	65
Kazakhstan	102	25	0	0	0	80	50
Kenya	137	1	0	0	0	69	33
Kiribati	182	0	0	0	0	25	12
Kuwait	0	0	0	207	0	0	100
Kyrgyzstan	125	33	0	0	0	49	39
Laos	120	13	0	0	0	74	42
Latvia	88	0	0	108	0	11	57
Lebanon	123	61	2	0	0	21	40
Lesotho	161	0	0	0	0	23	11
Liberia	139	22	2	0	0	44	32
Libya	182	11	1	0	1	12	12
Lithuania	54	0	0	99	0	54	73
Luxembourg	0	0	0	207	0	0	100
Madagascar	159	5	0	0	0	43	23
Malawi	57	18	0	0	0	132	72
Malaysia	53	125	0	0	0	29	74
Maldives	108	27	15	0	0	57	47
Mali	139	3	0	0	0	65	32
Malta	7	1	0	198	0	1	96
Marshall Islands	163	0	1	0	1	42	21
Mauritania	91	82	0	0	0	34	56
Mauritius	141	25	0	9	0	32	31
Mexico	55	0	0	0	0	152	73
Micronesia (Federated States of)	170	0	0	0	0	37	17
Monaco	0	0	0	138	0	0	66
Mongolia	126	5	26	2	3	45	39
Montenegro	148	0	0	0	0	59	28
Morocco	155	3	24	0	0	25	25
Mozambique	165	0	0	0	0	42	20
Myanmar	125	42	1	0	0	39	39
Namibia	132	4	0	0	0	71	36
Nauru	141	3	2	45	0	16	31
Nepal	32	53	24	0	3	95	84
Netherlands	0	0	0	207	0	0	100

New Zealand	0	0	0	207	0	0	100
Nicaragua	128	34	23	0	0	22	38
Niger	116	2	2	0	0	87	43
Nigeria	90	0	0	0	0	117	56
Niue	165	1	0	0	1	40	20
North Macedonia	75	70	0	0	11	51	63
Norway	0	0	0	207	0	0	100
Oman	56	0	0	144	0	7	72
Pakistan	166	2	0	0	6	33	19
Palau	113	0	16	45	0	33	45
Panama	113	0	25	45	0	24	45
Papua New Guinea	172	1	7	0	0	27	16
Paraguay	76	28	22	0	0	81	63
Peru	148	13	19	0	0	27	28
Philippines	130	18	0	0	0	59	37
Poland	72	0	0	126	0	9	65
Portugal	0	0	0	207	0	0	100
Qatar	0	0	0	207	0	0	100
Republic of Korea	0	0	0	198	0	9	100
Republic of Moldova	119	23	0	0	1	64	42
Romania	95	9	4	27	16	56	54
Russian Federation	70	62	0	27	0	48	66
Rwanda	147	3	0	0	0	57	28
Saint Kitts and Nevis	75	0	11	108	0	13	63
Saint Lucia	141	21	22	0	0	23	31
Saint Vincent and the Grenadines	165	0	10	1	0	31	20
Samoa	178	0	0	0	0	29	14
San Marino	0	0	0	161	0	0	77
São Tomé and Príncipe	78	5	8	0	0	116	62
Saudi Arabia	32	0	0	171	0	4	84
Senegal	130	22	8	0	0	47	37
Serbia	124	10	0	0	0	73	40
Seychelles	93	15	0	81	0	18	55
Sierra Leone	116	22	0	0	1	68	43
Singapore	0	0	0	207	0	0	100
Slovakia	35	14	0	144	0	14	83
Slovenia	0	0	0	207	0	0	100
Solomon Islands	179	1	0	0	0	27	13
South Africa	117	22	28	0	0	40	43
South Sudan	190	4	0	0	0	13	8
Spain	0	0	0	207	0	0	100

Sri Lanka	31	48	29	0	0	99	85
Sudan	68	77	3	0	14	45	67
Suriname	161	0	5	0	0	41	22
Sweden	0	0	0	207	0	0	100
Switzerland	0	0	0	207	0	0	100
Syria	180	3	2	0	7	15	13
Tajikistan	107	27	0	0	0	73	48
Thailand	82	5	0	0	0	120	60
Timor-Leste	56	1	0	0	0	150	72
Togo	130	19	10	0	0	48	37
Tonga	108	22	2	0	0	75	47
Trinidad and Tobago	38	0	6	153	0	10	81
Tunisia	152	0	22	0	0	33	26
Turkmenistan	147	6	0	1	2	51	28
Tuvalu	185	0	0	0	0	22	10
Türkiye	175	0	2	0	0	30	15
Uganda	94	16	1	0	0	96	54
Ukraine	124	5	18	0	2	58	40
United Arab Emirates	0	0	0	207	0	0	100
United Kingdom	0	0	0	207	0	0	100
United Republic of Tanzania	120	22	0	0	0	65	42
United States of America	0	0	0	207	0	0	100
Uruguay	60	0	21	99	0	27	71
Uzbekistan	149	4	1	0	0	53	28
Vanuatu	182	0	0	0	0	25	12
Venezuela	193	0	1	9	0	4	6
Viet Nam	104	21	22	1	7	52	49
Yemen	143	7	0	0	38	19	30
Zambia	91	16	0	1	0	99	56
Zimbabwe	150	15	4	0	0	38	27
World Bank high-income	0	0	0	10158	0	0	81
World Bank low-income	4934	682	139	1	45	2426	58
World Bank lower-middle-income	6336	768	431	4	101	2970	42
World Bank upper-middle-income	5827	796	439	4	51	2529	34
GBD high-income	256	1	80	6639	0	0	92

Note: Values represent the number of raw datapoints classified as belonging to the respective data quality score of 0-5 in each column.

S2.2 Statistical model to fill missingness in health expenditure variables

After the cleaning of the data previously described above, we used spatiotemporal Gaussian process regression (ST-GPR) to predict and fill out the missingness that existed in the resulting health expenditure dataset. ST-GPR is a stochastic modeling technique designed to detect signals amid noisy data. It also serves as a powerful tool for interpolating non-linear trends. Unlike classical linear models that assume that the trend underlying data follows a definitive functional form, GPR assumes that the specific trend of interest follows a Gaussian process, where each point can be estimated with mean and covariance.

The first step to implementing ST-GPR is to identify relevant covariates that would be helpful in predicting each health expenditure variable of interest. Using the following set of covariates, we estimated the first stage of the process (space-time) to predict and fill up the dependent variables. The covariates used are:

a) All-sector government expenditure per capita, logged
b) Lag-distributed income, logged
c) Healthcare Access and Quality Index, logged
d) Proportion of total population above the age of 64, logit transformed.

The dependent variables were logs of GHE per capita, PPP per capita, and OOP per capita. We also run these models sequentially so that the following model can include the previous component as a covariate. GHE per capita uses only the above covariates, while PPP per capita additionally included GHE per capita as a covariate, and OOP per capita additionally uses GHE per capita and OOP per capita as covariates.

Given the weight of data, we were able to adjust the weight of each data point that contributed to the likelihood function of the Gaussian process, by inflating the pointwise variance for data points with lower weights. For missing data points, the resulting uncertainty was determined by region-specific estimates. The final resulting dataset was a complete set of GHE, PPP, and OOP per capita estimates for 204 countries from 1995 through 2022, where the uncertainty around each point was constructed by simulating from a normal distribution. Goodness-of-fit was determined using out-of-sample validation and choosing the models with the best-performing set of hyperparameters. Overall, ST-GPR is a stochastic modeling technique that performs well at detecting signals among datasets that are noisy. It deviates from classic linear models that assume the trend of its underlying data must follow a definitive functional form. Instead, GPR assumes the trend must follow a Gaussian process form, having a covariance function and a pointwise mean. For the distribution of the Gaussian process, we used a Matérn covariance matrix. GPRs were run repeatedly to ensure that every country-year estimate per metric had 500 draws. This ST-GPR process follows the same form of that found in the Global Burden of Disease Study 2017 appendix.[3]

Figure S2.1 below shows the comparison of WHO GHED extracted THE data and IHME ST-GPR THE in per capita space from 2000–2022. Figure S2.2 shows the comparison of WHO GHED extracted THE data and IHME ST-GPR THE in per GDP space from 2000–2022.

Figure S2.1 Comparison of WHO GHED extracted and IHME ST-GPR THE in per capita space, 2000–2022

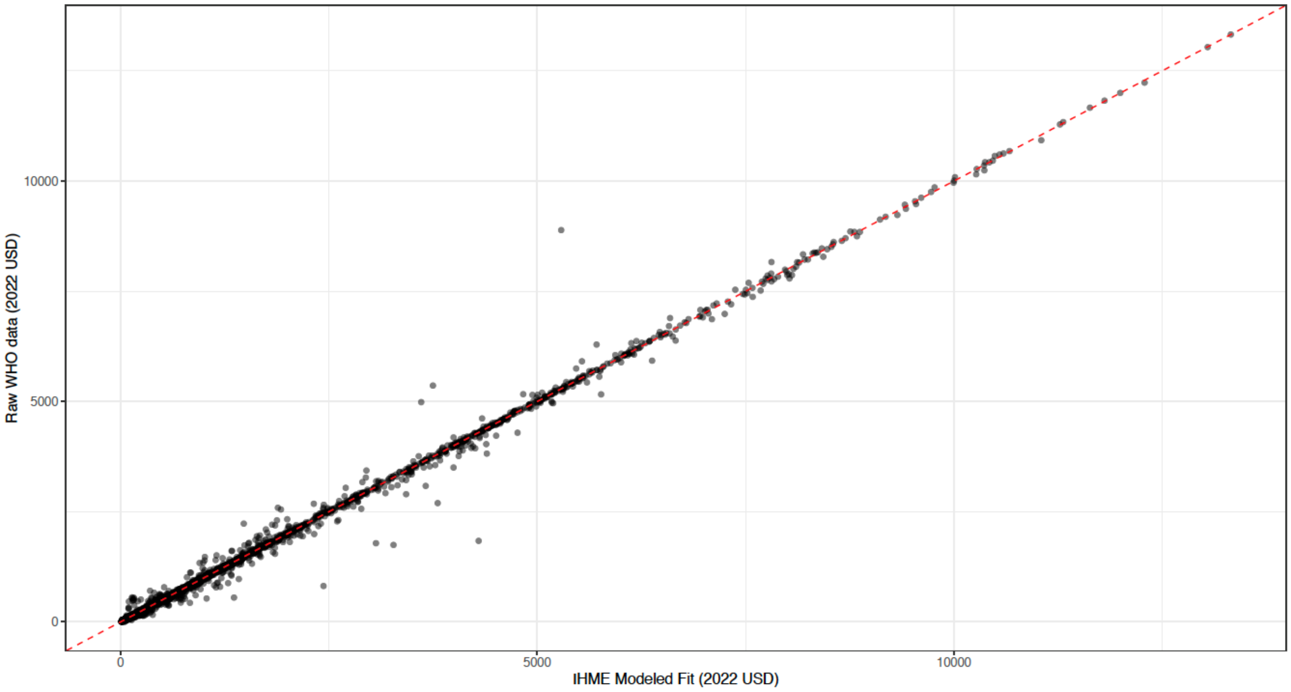

Figure S2.2 Comparison of WHO GHED extracted and IHME ST-GPR THE in per GDP space, 2000–2022

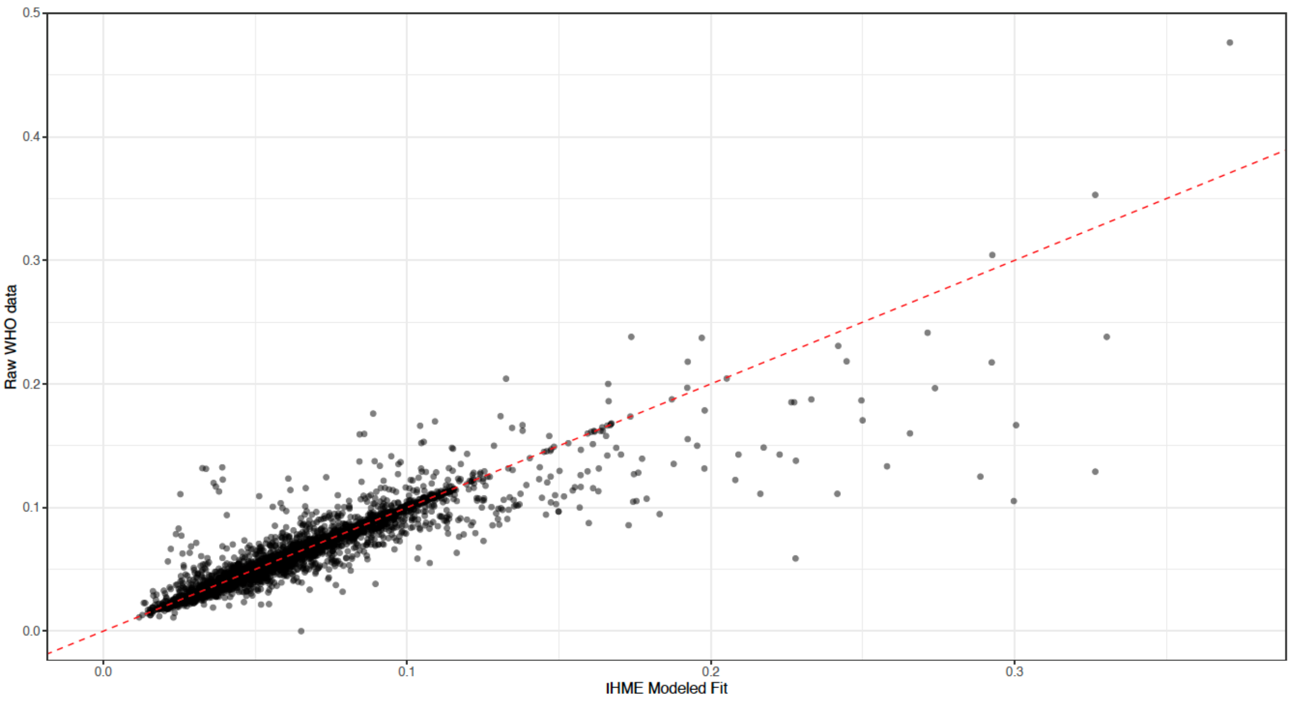

S2.3 Currency exchange and deflation

To convert a metric from real 2023 USD to real 2023 PPP, the following steps were taken. Real USD was exchanged to real LCU (local currency units) by multiplying with country-year-specific USD to LCU exchange rates. Then, values were converted from real LCU to real PPP (purchasing-power parity-adjusted dollars) using the country-year-specific LCU to PPP conversion series. Any currency deflation from nominal to real currency units or between real currency units in different base years were performed after converting either USD or PPP to LCU and used country-specific deflator series. All deflators and exchange rates were extracted from the World Bank,[4] International Monetary Fund,[5] Penn World Tables,[6] the United Nations National Accounts,[7] and the World Health Organization,[1] and were imputed to provide a complete series for each of the variables between 1950 and 2023. We then used several models including ordinary least-squares regression and mixed effects models, to complete each source series from 1950 to 2023, holding constant the last country-specific observed data point backwards in time.

Figure S2.3 shows the currency conversion process used in this work.

Figure S2.3 Currency conversion process diagram

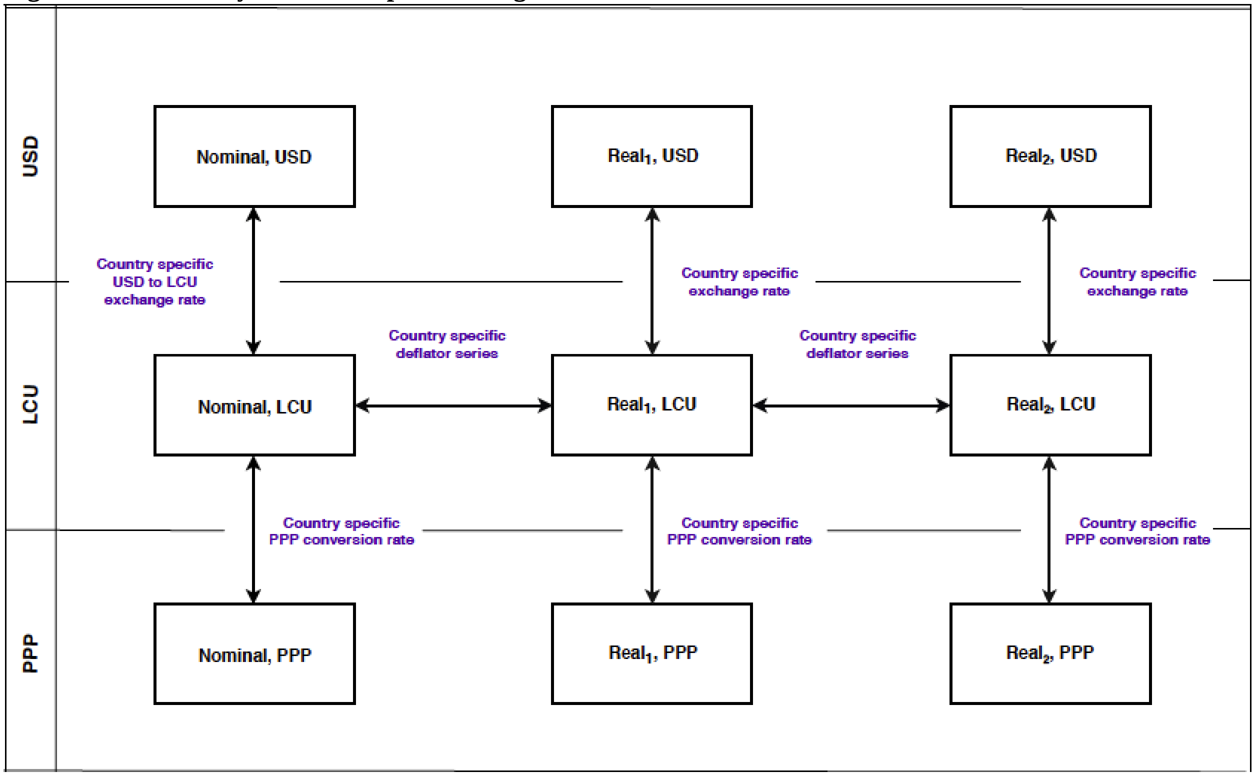

References

1 Global Health Expenditure Database. World Health Organization. http://apps.who.int/nha/database (accessed Feb 7, 2025).

2 World Bank Country and Lending Groups – World Bank Data Help Desk. World Bank. https://datahelpdesk.worldbank.org/knowledgebase/articles/906519-world-bank-country-and-lending-groups (accessed Feb 7, 2024).

3 Stanaway JD, Afshin A, Gakidou E, et al. Global, regional, and national comparative risk assessment of 84 behavioural, environmental and occupational, and metabolic risks or clusters of risks for 195 countries and territories, 1990–2017: a systematic analysis for the Global Burden of Disease Study 2017. The Lancet 2018; 392: 1923–94.

4 World Development Indicators (WDI) | Data Catalog. https://datacatalog.worldbank.org/dataset/world-development-indicators (accessed Feb 7, 2025).

5 World Economic Outlook Database April 2025. https://www.imf.org/en/Publications/WEO/weo-database/2025/April (accessed Feb 7, 2025).

6 Feenstra, Robert C., Robert Inklaar and Marcel P. Timmer (2015), "The Next Generation of the Penn World Table" American Economic Review, 105(10), 3150-3182, available for download at www.ggdc.net/pwt DOI:10.15141/S5Q94M.

7 National Accounts - Analysis of Main Aggregates (AMA). https://unstats.un.org/unsd/snaama/Basic (accessed Feb 7, 2025).

SECTION 3. TRACKING DEVELOPMENT ASSISTANCE FOR HEALTH FROM TRADITIONAL DONORS

List of figures

Figure S3.1 Comparing CRS commitments, CRS disbursements, and DAC commitments

Figure S3.2 CRS disbursement to commitment ratio and cutoff points by donor agency

Figure S3.3 One- to six-year disbursement schedules for bilateral channels

Figure S3.4 DAH as a percentage of corresponding budget data by bilateral agency

Figure S3.5 Malaria DAH to program areas as assigned by keyword search and PMI reports

Figure S3.6 Comparison of world Bank IDA Replenishment and DAC

Figure S3.7 Disbursements by the African Development Bank

Figure S3.8 Disbursements by Asian Development Bank

Figure S3.9 Disbursements by Inter-American Development Bank

Figure S3.10 The Global Fund's disbursements including Covid-19 Response Mechanism

Figure S3.11 The Global Fund's commitments and disbursements

Figure S3.12 Gavi's income and disbursements

Figure S3.13 Total revenue received by non-governmental organizations

Figure S3.14 Expenditure by non-governmental organizations

Figure S3.15 In-kind contributions by loan- and grant-making DAH channels of assistance

List of tables

Table S3.1. Summary of primary data sources and databases

Table S3.2. Data sources received via personal correspondence

Table S3.3. Additional data sources, databases, and model choices used for preliminary estimates of DAH

Table S3.4. Terms for keyword searches

Table S3.5. Additional health focus area categorizations

Table S3.6. World Bank's health sector and theme codes

Table S3.7 Summary of data sources for the regional development banks

Table S3.8 Summary of US non-governmental organizations in the study

Table S3.9 Summary of data sources for calculating in-kind contributions

Table S3.11 Summary of COVID data sources by channel

Table S3.12 Donor country cuts to development assistance for health in 2025

Box 3.1. EXAMPLE. Australia's primary and additional data sources

Box 3.2. EXAMPLE. Post-keyword search weighting

Box 3.3. EXAMPLE. Australia's CRS disbursement to commitment ratio and cutoff year

Box 3.4. EXAMPLE. Australia's one- to six-year disbursement schedules

Box 3.5. EXAMPLE. Australia's DAH as a percentage of corresponding budget data

S3.1 Overview

We summarize the original methodology as well as updates for this year's estimates below. A more detailed description of the original methodology used to obtain the estimates can be found in Dieleman et al.[1] All known, systematically reported, available data on health-related disbursements and expenditures were extracted, as well as income and revenue from existing project databases, annual reports, and audited financial statements. The channels included and the corresponding data sources are summarized in Table S3.1. Data sources obtained via personal correspondence are summarized in Table S3.2.

DAH for bilateral agencies included all health-related disbursements from bilateral donor agencies, excluding funds that they transferred to any of the other channels we tracked, to avoid double-counting. This information was extracted from the Creditor Reporting System (CRS) and Development Assistance Committee (DAC) databases of the Development Assistance Committee of the Organisation for Economic Co-operation and Development (OECD-DAC). In some cases, donor agencies did not report disbursement data to the CRS. A method for predicting disbursements from commitment data was implemented to address this challenge. For a detailed description of this method, see Tracking Development Assistance for Health from Bilateral Aid Agencies and the European Commission section below, as well as in Dieleman et al.[1]

For other grant- and loan-making institutions, annual disbursements on health grants and loans were similarly included, excluding transfers to any other channels and ignoring any repayments on outstanding debts. For a more detailed description of this process, see Dieleman et al.[1] The annual disbursements for grant- and loan-making institutions only reflect the financial transfers made by these agencies. Therefore, in-kind transfers from these institutions in the form of staff time for providing technical assistance and the costs of managing programs were estimated separately.[1]

Estimates of DAH for the United Nations (UN) agencies included annual expenditures on health both from their core budgets and from voluntary contributions. Calculating DAH for the United Nations Children's Fund (UNICEF) involved estimating the fraction of its total expenditure spent on health prior to 2001.[1]

Non-governmental organizations' (NGOs) DAH estimates utilized data from US government sources and GuideStar Research Fundamental Plus dataset. In previous years a survey of health expenditure for a sample of NGOs to estimate DAH was used, while in the 2019 and 2020 data update this year health expenditure was directly calculated using keyword search of project descriptions found on 990 forms.. Our dataset includes US based and internationally based NGOs receiving support from the US government; we were unable to include other NGOs due to the lack of audited and comparable data.

The database also included an analysis of the composition of health funding by recipient country, health focus area and program area. In this round of updates, methodological changes to the Foundations and NGOs estimation methods were made which are detailed in relevant channel sub-sections below.

For many channels, reporting-time lags prevent primary disbursement data for the most recent year(s). For those years, the values of DAH were predicted using channel-specific time trends. The methods employed to obtain these predictions are summarized in Table S3.3. In general, these methods depend on data availability. The estimates are based on channel-specific budget, commitment, and appropriations data, and in many cases assume the most recent

disbursement patterns persist. Due to the lack of more detailed disaggregated data, estimates for the most recent year are not provided for recipient countries.

In addition, for SWAP/HSS funding, we allocated SWAP/HSS projects with multiple health focus areas identified by a proportional allocation based on the relative proportions of the project going to the various health focus areas.

For projects that list a recipient as a region, we split DAH among the low- and middle-income countries that are eligible to receive DAH within that region based on known proportion of DAH. We do not split projects targeted at global initiatives and label the recipient as "global." For example, a project for vaccine development that has no specified recipient country would be classified as global.

For countries that only began existing in certain years, we backcasted DAH in years before their existence as follows. For countries that split off from parent countries, we calculated a three-year average ratio of child country DAH received to parent country DAH received. In years before the child country split off, DAH received by the parent country would have included DAH received in the region that would split off to become the child country. Therefore, we reallocated funding from the parent country, in all years before the child country split off, adding this proportion of the parent country's DAH to the child country's DAH and subtracting out this value from the parent country's DAH. By this method, total annual DAH between the parent and child country do not change, but the allocation of funding between the parent country and child country change. For any country that ceased to exist (such as former Yugoslavia and former USSR) and that had observed DAH received in certain years, we split the funding equally among its new constituent countries.

S3.1.1 Currency exchange and deflation

All results are presented in real 2023 US dollars. All disbursement sequences are converted into real 2023 US dollars by taking disbursements in nominal US dollars in the year of disbursement and adjusting these sequences into real 2023 US dollars using US gross domestic product (GDP) deflators.[99] All disbursement sequences in nominal currencies other than US dollars are converted into real 2023 US dollars by adjustment using country-year-specific rates from the OECD's exchange rate series from the OECD data explorer. If the current year is not yet available, the previous year's deflator value is utilized. OECD does not include an exchange series for the United Arab Emirates. IHME deflator estimates for the United Arab Emirates are leveraged for deflation. Analyses are conducted in Stata (versions 13.1 or 15.1) or R (version 4.4).

Table S3.1. Summary of primary data sources and databases

Channel	*Source*
Bilateral agencies	OECD-DAC and CRS databases, budget documents, and IATI[2,111]
European Commission	OECD-DAC and CRS databases[3]
Joint United Nations Programme on HIV/AIDS (UNAIDS)	Financial reports, audited financial statements, and budget[4]
United Nations Children's Fund (UNICEF)	Financial reports and audited financial statements[5–7]
United Nations Population Fund (UNFPA)	Financial reports and audited financial statements[8]
UNITAID	Project-level data from IATI database shared through correspondence, financial reports and audited financial statements[9]
Pan American Health Organization (PAHO)	Financial reports and audited financial statements[10]
World Health Organization (WHO)	Financial reports and audited financial statements, and IATI data.[11,111]
World Bank	Online project database, correspondence, and IATI data[12,13,111]
African Development Bank (AfDB)	Correspondence, replenishment reports, and compendium of statistics, and IATI data.[14,15,111]

Asian Development Bank (ADB)	Online project database, correspondence, and IATI data[81,98]
Inter-American Development Bank (IDB)	Online project database and correspondence, and IATI data.[16,17,111]
Gavi, the Vaccine Alliance	Online project database, cash received database, International Finance Facility for Immunisation (IFFIm) annual reports, Advance Market Commitment for Pneumococcal Vaccines (AMC) annual reports, correspondence, annual reports, and IATI data[18–21]
The Global Fund to Fight AIDS, Tuberculosis and Malaria (The Global Fund)	Online grant database, contributions report, and annual reports[22–24]
Coalition for Epidemic Preparedness and Innovation (CEPI)	Project-level data through correspondence
NGOs registered in the US	Candid Research Fundamentals PLUS dataset, United States Agency for International Development (USAID) Report of Voluntary Agencies (VolAg), tax filings, annual reports, financial statements, RED BOOK Expanded Database, and WHO's Model List of Essential Medicines, Guidestar Research Fundamentals Plus dataset[25–29]
Gates Foundation	Online grant database, IRS 990 tax forms, and correspondence[30,31]
Other private US foundations	VOLAG historical data, Foundation Center's grants database, and CRS
European Economic Area	Online project database and correspondence
China	Financial reports and yearbooks; AidData[33,34.113]

Table S3.2. Data sources received via personal correspondence

Channel	*Data received*
World Bank	Health project-level disbursement data, 1990–2021[13]
Gates	Health disbursement data, 2023 [31]
CEPI	Health disbursement data, 2022, 2023
IDB	Health project-level loan disbursement data, 2015–2024[17]
AfDB	Health project-level disbursement data, 1990–December 2024[35]
ADB	Health project-level disbursement data, 1990–2021[98]
UNITAID	Health project-level disbursement data, 2007–2024[36]
UAE bilateral	UAE Foreign Assistance in Health 1990–2008[37]
European Economic Area	Health project-level disbursement data, 2007–2021

Table S3.3. Additional data sources, databases, and model choices used for preliminary estimates of DAH

Channel	*Data source*	*Variables used*	*Years of budget data used for modeling**	*Years underlying DAH data not available; thus modeled**	*Model used*
National agencies					
Australia	Australia's International Development Assistance (2008–2024); Australia's Overseas Aid Program (1998–2008)[38,39]	Health official development assistance (ODA): International development assistance budget	1998–2024	2024	Percentage change in budgeted DAH applied to 2023 DAH estimate.
Austria	Austria Federal Ministry of Finance budget[40]	General ODA: Federal ODA budget	2007–2024	2024	Percentage change in budgeted ODA applied to 2023 DAH estimate.
Belgium	Project Budget General – general expenses[41]	General ODA: Foreign affairs, foreign trade development and cooperation	2000–2024	2024	Percentage change in budgeted ODA.
Canada	Canadian International Development Agency – Report on Plans and Priorities[42]	General ODA: Financial summary – planned spending	2006–2024	2024	Percentage change in budgeted ODA.
Czechia	Czech Development Agency activity plan[116]	General ODA: Budget of the CDA	2023-2024	2024	Percentage change in budgeted ODA
Denmark	Danish Ministry of Foreign Affairs Budget[43]	General ODA: Budgeted expenditures on overseas development assistance	2000–2024	2024	Percentage change in budgeted ODA.
Estonia	Estonia Ministry of Foreign Affairs[117]	General ODA: Budgeted expenditure on development cooperation and humanitarian aid.	2023-2024	2024	Percentage change in budgeted ODA
European Commission	General budget[44]	Data not used as they were	–	2024	Based on weighted

Channel	Data source	Variables used	Years of budget data used for modeling*	Years underlying DAH data not available; thus modeled*	Model used
		inconsistent with disbursements			average of trends in member countries
Finland	Document Assembly in budget years 1998–2023[45]	General ODA: Ministry of Foreign Affairs' administrative appropriations, international development	1998–2024	2024	Percentage change in budgeted ODA.
France	Budget and Financial documents[46,47]	General ODA: aggregated project data; Total ODA	2009–2024	2024	Percentage change in budgeted ODA.
Germany	Plan of the Federal Budget[48]	General ODA: Development expenditure	2003–2024	2024	Percentage change in budgeted ODA.
Greece	Ministry of Finance Budget (2013–2018); Hellenic Aid Annual Reports (2019-2023) [49,50]	General ODA; ODA commitments	1996–2023	2024	2024 ODA estimated using three year weighted average. Percentage change in estimated ODA used to model DAH.
Hungary	Hungary Ministry of Foreign Economy and Foreign Affairs[118]	General ODA: Budgeted operational expenditure of the ministry	2023-2024	2024	Percentage change in budgeted ODA.
Ireland	Department of Finance – budget[51]	International cooperation	2000–2024	2024	Percentage change in budgeted ODA.
Iceland	Iceland Financial Plan[119]	General ODA: Budgeted financing for international development cooperation	2023-2024	2024	Percentage change in budgeted ODA.
Italy	The Italian Agency for Development Cooperation[52]	General ODA: Net development corporation	2007–2024	2024	Percentage change in budgeted ODA.

Channel	Data source	Variables used	Years of budget data used for modeling*	Years underlying DAH data not available; thus modeled*	Model used
Japan	Highlights of the Budget for the Fiscal Year[53]	General ODA: Major budget expenditures	2003–2024	2024	Percentage change in budgeted ODA.
Korea, South	ODA Korea comprehensive implementation plan[54]	General ODA: Plan for international development cooperation	2008–2024	2024	Percentage change in budgeted ODA.
Lithuania	Ministry of Finance of Lithuania[120]	General ODA: budget for development cooperation	2023-2024	2024	Percentage change in budgeted ODA.
Luxembourg	State Budget[55]	General ODA: Ministry of Foreign Affairs – budgeted international development cooperation and humanitarian aid	2001–2024	2024	Percentage change in budgeted ODA.
Netherlands	Netherlands International Cooperation Budget	General ODA: Total annual official development assistance expenditure	2001–2024	2024	Percentage change in budgeted ODA.
New Zealand	Vote Foreign Affairs and Trade (1998–2001); VOTE Official Development Assistance (2002–2024)[56]	General ODA: Total annual official development assistance expenditure	1998–2024	2024	Percentage change in budgeted ODA.
Norway	Norwegian Ministry of Finance National Budget (2014–2024); Correspondence (2000–2013)[57,58]	General ODA: ODA budget	2000–2024	2024	Percentage change in budgeted ODA.
Poland	Poland Development Cooperation Plan[121]	General ODA: Budgeted development cooperaton	2023-2024	2024	Percentage change in budgeted ODA.
Portugal	Ministry of Finance and Public	General ODA: Integrated service	2003–2024	2024	Percentage change in budgeted ODA.

Channel	*Data source*	*Variables used*	*Years of budget data used for modeling**	*Years underlying DAH data not available; thus modeled**	*Model used*
	Administration State Budget[59]	expenditure – external cooperation budget			
Slovak Republic	State budget of the Slovak Republic[122]	General ODA: budgeted development cooperation expenditure across ministries	2023-2024	2024	Percentage change in budgeted ODA.
Slovenia	Adopted budget of the Republic of Slovenia[123]	General ODA: International development cooperation and humanitarian aid	2023-2024	2024	Percentage change in budgeted ODA.
Spain	Annual Plans of Spanish International Cooperation[60]	General ODA: Spanish total development cooperation	2003–2024	2024	Percentage change in budgeted ODA.
Sweden	Correspondence (2000–2010); Ministry of Foreign Affairs Budget (2010–2024)[61]	General ODA: Ministry for Foreign Affairs budgets for expenditure – international development cooperation	2000–2024	2024	Percentage change in budgeted ODA.
Switzerland	Foreign Affairs (2000–2006); Budget – Further Explanations and Statistics (2007–2024)[124]	General ODA: Direction of development and cooperation (2000–2006); foreign affairs – international cooperation, development aid (in the South and East) (2007–2016)	2000–2024	2024	Percentage change in budgeted ODA.
United Kingdom	FCDO Annual Report [62,63]	General ODA: assistance for international	2022–2024	2024	Percentage change in budgeted ODA.

Channel	*Data source*	*Variables used*	*Years of budget data used for modeling**	*Years underlying DAH data not available; thus modeled**	*Model used*
		development; Sum (revised) – aggregated project data			
United States	Budget of the US Government (2005–2024), CDC Congressional Justification 2012–2024[64,65]	Global health ODA: Planned foreign assistance for health; Department of Health and Human Services global health budget	2005–2024	2024	Percentage change in budgeted ODA.
UN agencies					
WHO	Programme budget[66]	DAH budget: Programme budget	2002–2024	2024	Weighted average of DAH/budget
UNAIDS	Unified Budget and Workplan, bienniums 2002–2017, 2018–2019, 2020–2021, 2022–2023[67,68]	DAH budget: Unified Budget and Workplan	2002–2024	2024	Weighted average of DAH/Core Budget
UNICEF	Financial report and audited financial statements[7,69,70]	Total expenditure; Total health expenditure	1990–2024	2024	Weighted average of DAH/budget
UNFPA	Audited Financial report, statistical and financial review, contributions report, annual report, and Structured Funding dialogue[71,72]	Total health expenditure	2002–2024	2024	Weighted average of DAH/budget
PAHO	Proposed program budget[10]	Total regular budget, estimated voluntary contributions	2000–2024	2024	Weighted average of DAH/budget
NGOs	VolAg (1990–2014), GuideStar (2015–2018), sample of top NGOs[25,26]	Revenue breakdowns for: US public, non-US public, private,	1990–2018	2019–2024	Regression on DAH, US GDP, and USAID and private voluntary

Channel	Data source	Variables used	Years of budget data used for modeling*	Years underlying DAH data not available; thus modeled*	Model used
		in-kind, BMGF; total overseas expenditures			organization (PVO) revenue

* Years of budget data used for modeling versus years underlying DAH data unavailable thus modeled

The data used to estimate DAH by channel vary across channels. Table S3.1 reports our primary data used for each channel. Due to reporting lags, there are some years we need to estimate disbursement using additional data sources. These additional data sources, the years in which the primary data are modeled, the years the additional data are available, and the methods for this estimating these modeled years are reported in Table S3.3. Years of budget data used for modeling are the years of additional data available to us. We rely on historical trends to inform our estimates, so we rely on many years of additional data despite only modeling a few years of primary data. Years underlying DAH data unavailable thus modeled are the years the primary data are incomplete and thus estimated using additional data. See example below for more details for Australia.

Box 3.1. EXAMPLE. Australia's primary and additional data sources

Project-level data for health-related projects funded by Australia's bilateral aid agencies are available from the OECD's CRS database through 2023. This is the primary data source used to estimate DAH channeled by Australian aid agencies, as described in Table S3.1. 2024 is incomplete because of lags in reporting. To estimate DAH disbursed for 2024, additional data are available from Australia's International Development Assistance budget (2008–2024) and Australia's Overseas Aid Program budget (1998–2008), as described in Table S3.3. These sources provide health-specific official development assistance (ODA) budgeted by Australia, 1998–2023. We convert countries' budgeted ODA, as given in nominal local currency units, to nominal US dollars using the OECD's currency exchange rate series based on USD monthly averages. To estimate DAH disbursed in 2024 for Australia, we calculate the percent change in budgeted DAH between 2023 and 2024 and apply this to our final estimate of 2023 DAH. For other agencies, we may calculate the ratio of disbursed DAH relative to budgeted DAH or ODA, and then combine the most recent three ratios into a single estimate by taking a weighted average, weighting substantially higher the most recent year. We would then multiply this ratio – the estimated disbursed DAH to budgeted DAH or ODA – by the 2024 budgeted DAH to estimate disbursed DAH in that year. These methods are described more fully in Dieleman et al.[1]

S3.1.2 DISAGGREGATING BY HEALTH FOCUS AREA

Similar to our previous work, the analysis of health focus areas included assessments of development assistance for HIV/AIDS, tuberculosis (TB), malaria, reproductive and maternal health, newborn and child health, other infectious diseases (including COVID-19), non-communicable diseases, and SWAps and health system strengthening by using keyword searches within descriptive fields. These were chosen as the areas of focus because of their relevance to current policy debates about global health financing and data availability.

In effect, DAH was disaggregated into nine health focus areas: HIV/AIDS, tuberculosis, malaria, reproductive and maternal health, newborn and child health, non-communicable diseases, SWAps/health sector support, other infectious diseases, and other. For most data sources, project-level data were available only through 2020. Methods to estimate health focus area allocations for 2023 are described in more detail below. Keyword searches were

performed for a subset of global health channels that provide project-level data with project titles or descriptions. These sources include the bilateral development assistance agencies from 31 DAC member countries, the EC, one DAC participant country, the Global Fund, the World Bank, Asian Development Bank, African Development Bank, Inter-American Development Bank, Bill & Melinda Gates Foundation, non-governmental organizations, US foundations, Wellcome Trust, and the European Economic Area. The keywords used are outlined in Table S3.4 below. Descriptive fields were adjusted so that they were all in capitalized letters, special characters were modified, and search terms with multiple words were put between quotation marks. All keywords were translated into nine major languages (English, Spanish, French, Portuguese, Italian, Dutch, German, Norwegian, and Swedish) used in the OECD CRS, checked for double meanings across all languages, and adjusted accordingly.

Total DAH was split across the health focus areas using weighted averages based on the number of keywords present in each project's descriptive variables. If, for example, three keywords suggested the project focused on HIV/AIDS and two keywords related to tuberculosis, three-fifths of the project's total DAH was allocated to HIV/AIDS and two-fifths was allocated to tuberculosis. To account for the sensitivity of this method, several checks were implemented after the keyword searches to ensure the project was accurately categorized. First, projects that were tagged as child and newborn vaccines and other infectious diseases were categorized as child and newborn vaccines only. Second, projects that were tagged as one of the three major infectious diseases (HIV/AIDS, tuberculosis, or malaria) and other infectious diseases were categorized under only HIV/AIDS, tuberculosis, or malaria.

Box 3.2. EXAMPLE. Post-keyword search weighting

A project in the CRS database had a value of $1,000 of DAH. A keyword search conducted on this project's title and description tagged five keywords: 3 keywords related to HIV/AIDS and 2 keywords related to tuberculosis. Therefore, $600, or 3/5 of total DAH, was allocated to HIV/AIDS, while $400, or 2/5 of total DAH, was allocated to tuberculosis.

In addition to keyword searches, funds were allocated to health focus areas based on characteristics of the channel or additional channel variables. For the bilateral agencies and the EC, purpose codes from the CRS were used to supplement keyword searches. For the World Bank-IDA and -IBRD, health focus areas were also determined by the project sector codes and theme codes, which included percentages of health funds that targeted each theme. All funds from Gavi were allocated to child and newborn vaccines, health system strengthening, and non-communicable diseases, and all funds from UNICEF to HIV/AIDS prevention, newborn and child health, Ebola, and reproductive and maternal health – other (plus a very small amount being unallocable). Funds from the Global Fund were distributed to malaria, HIV/AIDS, TB, and health sector support based on disease components. Within each disease component, keyword searches on programmatic budget data and project descriptions were conducted to distribute among program areas. Funds from UNAIDS were allocated to HIV/AIDS and TB, with specific program areas determined by budget information. Unitaid funds were allocated based on assignments made by correspondence. UNFPA funds were allocated to specific health focus areas based on project expenditure data from its financial review report. PAHO and WHO funds were allocated to specific health focus areas based on project expenditure data from correspondence budget data and their proposed program budgets. For all channels, projects listed as HIV/TB were distributed evenly among the two health focus categories. See Table S3.5 below for more details on these categorizations.

Table S3.4. Terms for keyword searches

Health focus area level	*Program area*	*Keywords*
HIV/AIDS	HIV envelope/other	" HUMANIMMUNODEFVIRUS " " SIDA " " OVC " " H I V " " HIV " " AIDS " " HUMAN IMMUNODEFICIENCY " " REVERSE TRANSCRIPTASE INHIBITOR " " ACQUIRED IMMUNE DEFICIENCY SYNDROME " " ACQUIRED IMMUNNODEFICIENCY " "RETROVIRAL " " VCT " " MALE CIRCUMCISION" " ART " " ARV " " CD4 COUNT " " HAART " " PMTCT " " MOTHER TO CHILD TRANSMISSION" " MOTHER TO CHILD AIDS TRANSMISSION" " PARENT TO CHILD TRANSMISSION" " PRESIDENT S EMERGENCY PLAN FOR AIDS RELIEF " " PEPFAR " " THREE DISEASES FUND " " 3 DISEASES FUND " " EMTCT " " 90 90 90 " " MOTHERTOCHILD"
	Care and support	" CAREANDSUPPORT " " CARE ACTIVIT" " PAIN RELIEF " " SYMPTOM RELIEF " " PSYCHO SOCIAL SUPPORT " " CHRONICALLY ILL " " CLINICAL MONITORING " " CARE AND SUPPORT " " PSYCHOLOGICAL SERVICE" " PSYCHOLOGICAL SUPPORT " " PSYCHOSOCIAL SUPPORT " " PSYCHOSOCIAL SERVICE" " MATERIAL SUPPORT " " HOME BASED CARE " " PALLIATIVE CARE "
	Counseling and testing	" COUNSELING " " TESTING " " VCT " " COUNSELLING " " COUNSELINGANDTESTING " " DIAGNOS"
	Orphans and vulnerable children	" VULNERABLECHILD" " OVC " " ORPHAN" " VULNERABLE CHILD" " INFECTED CHILD"
	Prevention of mother-to-child transmission (PMTCT)	" MOTHERTOCHILD" " MOTHER TO CHILD" " PARENT TO CHILD" " PMTCT " " EMTCT " " OPTION B "
	Prevention	" CONDOM" " PREVENT" " HIV EDUCATION " " AIDS EDUCATION " " REDUCING THE TRANSMISSION OF HIV " " REDUCE THE TRANSMISSION OF HIV " " MALE CIRCUMCISION" " SAFE BLOOD SUPPL" " SAFE INJECTION" " ABSTINENCE " " BLOOD SAFETY " " MICROBICIDE" " HARM REDUCTION " " PREP " " PEP " " PROPHYLAXIS " " ABCD " " BE FAITHFUL " " EARLY DETECTION "
	Treatment	"RETROVIRAL " " TREAT" " ART " " ARV " " CD4 COUNT " " HAART " " VIRAL LOAD " " VIRAL BURDEN " " VIRAL TITER " " DRUG REGIMEN" " FIRST LINE " " REVERSE TRANSCRIPTASE INHIBITOR "
	Antimicrobial resistance	" ANTIMICROBIAL RESISTAN" " ANTI MICROBIAL RESISTAN" " ANTIBIOTIC RESISTAN" " AMR " "DRUG RESISTAN" " MDR " " XDR " " RESISTANCE TESTING " " DRUG SUSCEPTIBILITY TESTING " " DST " " SECOND LINE "
Tuberculosis	Tuberculosis envelope/other	" TUBERCULOSIS " " TB " " TBC " " TUBERCULAR" " DOTS " " DIRECTLY OBSERVED TREATMENT " " RIFAMPICIN " " ISONIAZID " " THREE DISEASES FUND " " 3 DISEASES FUND " " RIFAMPIN "
	Treatment	" TREAT" " DOTS " " FIRST LINE " " DRUGS " " RIFAMPICIN " " RIFAMPIN " " ISONIAZID " " INH " " PYRAZINAMIDE " " PZA " " ETHAMBUTOL " " EMB " " STREPTOMYCIN " " SM " " STM " " PATIENT KIT " " INJECTABLE AGENT" " FLUOROQUINOLONES " " REGIMEN" " CASE MANAGEMENT " " ANTIMICROBIAL THERAPY " " DRUG SUSCEPTIBLE " " DRUG SENSITIVE "
	Diagnosis	" MICROSCOPY " " SPUTUM " " SMEAR " " CULTURE " " BACTERIOLOGICALLY CONFIRMED " " LABORATORY

Health focus area level	*Program area*	*Keywords*
		CONFIRMED " " GENEXPERT " " XPERT MTB RIF " " CHEST X RAY" " DIAGNOS" " LINE PROBE ASSAY "
	Antimicrobial resistance	" ANTIMICROBIAL RESISTAN" " ANTI MICROBIAL RESISTAN" " ANTIBIOTIC RESISTAN" " AMR " "DRUG RESISTAN" " MDR " " XDR " " RESISTANCE TESTING " " DRUG SUSCEPTIBILITY TESTING " " DST " " SECOND LINE "
Malaria	Malaria envelope/other	" MALARIA " " FALCIPARUM " " ANOPHELES " " ARTEMISININ " " PRIMAQUINE " " INDOOR RESIDUAL SPRAY" " INDOORRESIDUALSPRAY" " IRS " "VIVAX " " BEDNET" " BED NET" " SMITN " " ITN " " LLIN " " INSECTICIDAL NET" " INSECTICIDE TREATED NET" " THREE DISEASES FUND " " 3 DISEASES FUND " " CHLOROQUINE " " ANTI MALARIAL " " ANTIMALARIAL "
	Diagnosis	" DIAGNOS" " CASE DETECTION " " MICROSCOPY " " BLOOD SURVEY" " BIOLOGICAL TESTING " " EDT " " LAMP " " RDT "
	Community outreach	" COMMUNITYOUTREACH " " OUTREACH " " COMMUNITY MOBILIZATION" " AWARE" " COMMUNICATION STRATEGY " " SOCIAL COMMUNICATION " " PARTNERSHIP" " ACTIVITIES NEAR COMMUNITIES " " BCC " " BEHAVIORAL CHANGE COMMUNICATION " " BEHAVIOURAL CHANGE COMMUNICATION " " BEHAVIOR CHANGE COMMUNICATION " " BEHAVIOUR CHANGE COMMUNICATION " " SOCIAL MOBILIZATION "
	Vector control: bednets	" BEDNET" " BED NET" " SMITN " " ITN " " LLIN " " INSECTICIDAL NET" " INSECTICIDE TREAT"
	Vector control: irs	" INDOORRESIDUALSPRAY" " IRS " " REDUCE THE PARASITE RESERVOIR " " FOGGING " " COILS " " LARVICID" " LARVACID" " VECTOR CONTROL" "RESIDUAL SPRAY" " RESIDUALSSPRAY " "INDOOR SPRAY" " INDOORSPRAY "
	Vector control: other than bednets and irs	" PREVENT" " IPT " " SMC " " SEASONAL MALARIA CHEMOPREVENTION "
	Treatment	" ARTEMISININ " " PRIMAQUINE " " ACT " " DRUG" " TREAT" " CASE MANAGEMENT " " COMBINATION THERAPY " " ANTI MALARIAL " " ANTIMALARIAL " " CHLOROQUINE "
	Antimicrobial resistance	" ANTIMICROBIAL RESISTAN" " ANTI MICROBIAL RESISTAN" " ANTIBIOTIC RESISTAN" " AMR " "DRUG RESISTAN" " MDR " " XDR " " RESISTANCE TESTING " " DRUG SUSCEPTIBILITY TESTING " " DST " " SECOND LINE "
Reproductive and maternal health	envelope/other	" FERTILITY " " FAMILY PLANNING " " FP " " BIRTH" " WOMEN HEALTH " " WOMEN S HEALTH " " WOMENS HEALTH " " CONTRACEP" " IPPF " " PLANNED PARENTHOOD " " ABORTION" " UNFPA " " POSTPARTUM " " POST PARTUM " " MATERNAL " " MATERNITY " " MOTHER" " SBA " " ANTENATAL " " PRENATAL " " PERINATAL " " POSTNATAL " " FETUS" " FETAL" " IPTP " " REPRODUCTIVE HEALTH " " OBSTETRIC" " PREGNANC" " RH " " REPROD " " RHCS " " SEXUAL HEALTH " " SYPHILIS " " FISTULA " " ANEMI" " ANAEMI" " FOETUS" " FOETAL " " FGM " " FEMALE GENITAL MUTILATION " " FEMALE GENITAL CUTTING " " FEMALE CIRCUMCISION " " SBAS " " OBSTRUCTED LABOR " " OBSTRUCTED LABOUR " " MNCH" " RNCH " " RCH " " RNH " " MNH " " MCH " " EMAS " " MCNH " " PMNCH " "ECLAMPSIA " " PRETERM " " ANC " " WCAH " " TBA " " TBAS " " BIRTH ATTENDANT" " CESAREAN" " CAESAREAN" " C SECTION" "

Health focus area level	*Program area*	*Keywords*
		STI " " STD " " SEXUALLY TRANSMITTED " " CHILD MARRIAGE " " EARLY MARRIAGE " " FORCED MARRIAGE " " INFORMAL MARRIAGE "
	Family planning	" FERTILITY " " FAMILY PLANNING " " FP " " BIRTH SPACING " " CONTRACEPT" " FAMILY SIZE" " IPPF " " PLANNED PARENTHOOD " " ABORTION" " BIRTH CONTROL " " CONDOM" " IUD " " VASECTOMY " " TUBULAR LIGATION"
	Maternal health	" POSTPARTUM " " POST PARTUM " " MATERNAL HEALTH " " MATERNAL MORTALITY " " MATERNAL DEATH" " SAFE MOTHERHOOD " " BIRTH ATTENDANT" " SBA " " ANTENATAL " " PRENATAL " " PERINATAL " " POSTNATAL " " FETUS" " FETAL" " IPTP " " MATERNITY " " OBSTETRIC" " PREGNANC" " FISTULA " " SEPSIS " " SEPTICEMIA " " ANEMI" " ANAEMI" " FOETUS" " FOETAL " " SBAS " " OBSTRUCTED LABOR " " OBSTRUCTURED LABOUR " " DELIVERY ROOM" " CHILD DELIVERY " " MIDWIV" " MIDWIFE" "ECLAMPSIA " " PRETERM " " ANC " " TBA " " TBAS " " CESAREAN" " CAESAREAN" " C SECTION" " MNH " " MCH " " MNCH " " MCNH "
Newborn and child health	envelope/other	" NEONATAL " " PERINATAL " " POSTNATAL " " MALNUTRITION " " VITAMIN A " " BREAST FE" " BREASTFE" " MICRONUTRIENT" " FORTIFICATION " " STUNT" " WASTING " " BABY FRIENDLY HOSPITAL INITIATIVE " " BREASTMILK " " BREAST MILK " " IODINE " " IODIZED " " IODIZATION " " VAD " " LACTAT" " FOLIC ACID " " FOLAT" " VACCIN" " IMMUNIZ" " POLIO " " DIPHTHERIA " " TETANUS " " PERTUSSIS " " DTP " " HIB " " ROTAVIRUS " " MEASLES " " IMMUNIS" " HEPB " " INJECTION SAFETY " " RUBELLA " " MENINGITIS " " PENTA " " PENTAVALENT " " PNEUMONIA " " PNEUMOCOCC" " HAEMOPHILUS INFLUENZAE " " TETRA " " GAVI " " CHILDHEALTH " " CHILD HEALTH " " CHILDREN " " INFANT" " NEWBORN" " CHILD MORTALITY " " UNDER FIVE MORTALITY " " CHILD SURVIVAL " " CHILDHOOD ILLNESS" " LRI " " RESPIRATORY INFECTION" " DIARRHEA" " DIARRHOEA" " ORAL REHYDRATION " " ORT " " ORS " " UNICEF " " MNCH" " RNCH " " RCH " " RNH " " MNH " " MCH " " EMAS " " MCNH " " POLIOVIRUS " " LRTI " " GPEI " " GLOBAL POLIO ERADICATION INITIATIVE "
	Child/newborn nutrition	" NUTRITION" " MALNUTRITION " " BIRTH WEIGHT " " BIRTHWEIGHT " " VITAMIN A " " BREAST FE" " BREASTFE" " FEEDING " " MICRONUTRIENT" " ZINC " " FORTIFICATION " " STUNT" " WASTING " " UNDERWEIGHT " " BABY FRIENDLY HOSPITAL INITIATIVE " " BREASTMILK " " BREAST MILK " " IODINE " " IODIZED " " IODIZATION " " VAD " " LACTAT" " FOLIC ACID " " FOLAT" " IRON " " DEWORMING "
	Child/newborn vaccines	" POLIO " " VACCIN" " IMMUNIZ" " DIPHTHERIA " " TETANUS " " PERTUSSIS " " DTP " " HIB " " ROTAVIRUS " " MEASLES " " IMMUNIS" " HEPB " " INJECTION SAFETY " " RUBELLA " " MENINGITIS " " PENTA " " PENTAVALENT " " PNEUMONIA " " PNEUMOCOCC" " HAEMOPHILUS INFLUENZAE " " TETRA " " GAVI " " POLIOVIRUS " " DPT " " PCV " " GVAP " " VACCINE ACTION PLAN " " IA2030 " " GPEI " " GLOBAL POLIO ERADICATION INITIATIVE "

Health focus area level	*Program area*	*Keywords*
Non-communicable diseases	envelope/other	" TOBACCO" " SMOK" "CIGAR" " FCTC " " TFI " " SCHIZOPHRENIA " " MENTAL HEALTH " " NEUROTIC " " NEUROSIS " " NEUROSES " " NEUROLOGICAL" " PSYCHOLOG" " PSYCHIATR" " EMOTIONAL DISORDER" " OBSESSIVE COMPULSIVE " " OCD " " PTSD " " POST TRAUMATIC " " POSTTRAUMATIC " " ALCOHOL DEPENDEN" " ALCOHOL ABUSE " " ADDICTION " " BEHAVIORAL DISORDER" " DRUG ABUSE " " SUBSTANCE ABUSE " " OPIOID " " COCAINE " " AMPHETAMIN" " DEPRESSIVE DISORDER" " DEPRESSION " " DYSTHYMIA " " BIPOLAR " " ANXIETY " " EATING DISORDER " " PHOBIA " " DRUG DEPENDEN" " ATTENTION DEFICIT HYPERACTIVITY DISORDER " " ADHD " " PANIC DISORDER" " SELF HARM " " STRESS DISORDER" " SUBSTANCE USE DISORDER" " DRUG USE DISORDER" " MENTAL ILLNESS" " MENTAL DISORDER" " PSYCHOSOCIAL " " PSYCHO SOCIAL " " HEROIN " " OXYCODONE " " ATTENTION DEFICIT DISORDER " " SUICIDE PREVENTION" " HEADACHE " " ANOREXIA " " BULIMIA " " HYPERKINETIC DISORDER" " PERSONALITY DISORDER" " FETAL ALCOHOL SYNDROME" " PSYCHOSIS " " DOWN SYNDROME " " DOWN S SYNDROME " " DOWNS SYNDROME " " AUTISM " " ASPERGER " " DEVELOPMENTAL DISORDER" " CONDUCT DISORDER" " INTELLECTUAL DISABILIT" " MENTAL DISAB" " MENTAL RETARDATION " " ALZHEIMER" " DEMENTIA" " EPILEPSY " " MIGRAINE " " PARKINSON" " MENTALLY DISAB" " NERVOUS SYSTEM" " SYNAPSE" " NON COMMUNICABLE " " NONCOMMUNICABLE " " CANCER" " CHEMOTHERAPY " " RADIATION " " NEOPLAS" " TUMOR " " LEUKEMIA " " LYMPHOMA " " MYELOMA " " HPV " " HUMAN PAPILLOMA VIRUS " " HEP C " " HEPATITIS C " " DIABET" " INSULIN " " ENDOCRINE " " RHEUMAT" " ISCHAEMIC " " ISCHEMIC " " CIRCULATORY " " CIRRHOSIS " " DIGESTIVE DISEASE" " OTHER DIGESTIVE " " PEPTIC " " APPENDICITIS " " GASTRITIS " " GENITOURINARY " " UROGENITAL " " MUSCULOSKELETAL " " GOUT " " BACK PAIN " " MACULAR " " HEARING " " AUDIOLOG" " PERIODONTAL " " CARIES " " CONGENITAL " " OBESITY " " OVERWEIGHT " " GLAUCOMA " " HYPERTENSI" " HERNIA " "ARTHRITIS " " CLEFT LIP" " CLEFT PALATE" " PHENYLKETONURIA " " SICKLE CELL" " DREPANOCYTOSIS " " HEMOPHILIA " " HAEMOPHILIA " " THALASSEMIA " " GENETIC DISORDER" " HEART DISEASE" " CHRONIC RESPIRATORY " " COPD " " STROKE " " CATARACT" " CHRONIC OBSTRUCTIVE PULMONARY DISEASE" " ASTHMA " " SKIN DISEASE" " DERMATITIS " " PSORIASIS " " SCABIES " " PHYSICAL DISAB" " DENTAL " " ORAL HEALTH " " CVD " " IHD " " CKD " " KIDNEY DISEASE" " MSK " " EYE " " CEREBROVASCULAR " " VASCULAR " " BLOOD PRESSURE " " ACUTE GLOMERULONEPHRITIS " " ALOPECIA AREATA " " ANEURYSM " " ANGINA " " ARTERY " " ATHEROSCLEROSIS " " ATRIAL FIBRILLATION" " ATRIAL FLUTTER " " BENIGN PROSTATIC HYPERPLASIA " "BLASTOMA" " BLIND " " PREVENTABLE BLINDNESS " " AVOIDABLE BLINDNESS " " BLOOD DISORDER" "

Health focus area level	Program area	Keywords
		BRONCHITI" " CARCINOMA " " CARDIAC " " CARDIO" " CELLULITIS " " CEREBRAL " " CORONARY " " DEAF" " DECUBITUS ULCER " "DIALYSIS" " DUODENITIS " " ECZEMA " " EKZEMA " " EDENTULISM " " ENDOCARDITIS " " FIBROSIS " " G6PD DEFICIENCY " " GALL BLADDER " " BILE DUCT " " GLYCEMI" " GLYCAEMI" " HEMOGLOBINOPATH" " HEMOLYTIC ANEMIA " " HODGKIN" " INSOMNIA " " INTERSTITIAL LUNG DISEASE" " INTESTINAL OBSTRUCTION" " LEUKAEMIA " " MELANOMA" " MULTIPLE SCLEROSIS " " MYOCARD" " NCD " " NECK PAIN " " NEPHRITIS " " NEPHROSIS " " NEURAL TUBE DEFECT" " NEURODEGENERATIVE " " INFLAMMATORY BOWEL" " ONCOLOG" " OPTICAL " " OSTEOMYELITIS " " OTITIS MEDIA " " PANCREATITIS " " PARALYTIC ILEUS " " PERITONEAL " " PNEUMOCONIOSIS " " PROSTATE " " PRURITUS " " SARCOIDOSIS " " PYELONEPHRITIS " " REFRACTIVE ERROR" " RENAL " " RETINA " " SARCOMA " " SUBCUTANEOUS DISEASE" " URINARY DISEASE" " URINARY TRACT INFECTION" " UROLITHIASIS " " URTICARIA " " VENTRICULAR " " VISION LOSS " " ACCOMODATION DISORDER" " SENSE ORGAN " " GUILLAIN BARRE SYNDROME" " IMPETIGO " " LOSE WEIGHT " " BIRTH DEFECT" "PAPILLOMAVIRUS" " GENE DEFECT" " PHYSICALLY DISAB" " TUMOUR" " BRAIN INJUR" " MAMMOGRA" " ANTITUMOR " " ANTITUMOUR " " BARIATRIC" " FATTY LIVER" " IMMUNOTHERAPY " " CHROMOSOMAL ABERRATION" " PERIODONTITIS " " OSTEOPOROSIS " "MALIGNANC" " NEURON"
	Mental health	" SCHIZOPHRENIA " " MENTAL HEALTH " " NEUROTIC " " NEUROSIS " " NEUROSES " " NEUROLOGICAL" " PSYCHOLOG" " PSYCHIATR" " EMOTIONAL DISORDER" " OBSESSIVE COMPULSIVE " " OCD " " PTSD " " POST TRAUMATIC " " POSTTRAUMATIC " " ALCOHOL DEPENDEN" " ALCOHOL ABUSE " " ADDICTION " " BEHAVIORAL DISORDER" " DRUG ABUSE " " SUBSTANCE ABUSE " " OPIOID " " COCAINE " " AMPHETAMIN" " DEPRESSIVE DISORDER" " DEPRESSION " " DYSTHYMIA " " BIPOLAR " " ANXIETY " " EATING DISORDER " " PHOBIA " " DRUG DEPENDEN" " ATTENTION DEFICIT HYPERACTIVITY DISORDER " " ADHD " " PANIC DISORDER" " SELF HARM " " STRESS DISORDER" " SUBSTANCE USE DISORDER" " DRUG USE DISORDER" " MENTAL ILLNESS" " MENTAL DISORDER" " PSYCHOSOCIAL " " PSYCHO SOCIAL " " HEROIN " " OXYCODONE " " ATTENTION DEFICIT DISORDER " " SUICIDE PREVENTION" " HEADACHE " " ANOREXIA " " BULIMIA " " HYPERKINETIC DISORDER" " PERSONALITY DISORDER" " FETAL ALCOHOL SYNDROME" " PSYCHOSIS " " DOWN SYNDROME " " DOWN S SYNDROME " " DOWNS SYNDROME " " AUTISM " " ASPERGER " " DEVELOPMENTAL DISORDER" " CONDUCT DISORDER" " INTELLECTUAL DISABILIT" " MENTAL DISAB" " MENTAL RETARDATION " " ALZHEIMER" " DEMENTIA" " EPILEPSY " " MIGRAINE " " PARKINSON" " MENTALLY DISAB" " NERVOUS SYSTEM" " SYNAPSE"

Health focus area level	*Program area*	*Keywords*
	Tobacco	" TOBACCO" " SMOK" "CIGAR" " FCTC " " TFI "
SWAps/ Health sector support	envelope/other	" SWAP" " TRAINING " " CAPACIT" " DATA SYSTEM" " SECTOR WIDE APPROACH" " HEALTH SYSTEM" " SECTOR PROGRAM" " BUDGET SUPPORT" " SECTOR SUPPORT " " HSS " " TRACKING PROGRESS " " SKILLED WORKER" " HEALTH WORKER" " SKILLED STAFF " " HEALTH PROFESSIONAL" " FACILITIES " " ESSENTIAL MEDICINES " " POLICY DEVELOPMENT" " TRAINING FACILIT" " TRAINING EQUIPMENT" " TRAINING EQMT " " INSTITUTIONAL STRENGTHENING " " HSPSP " " M&E " " M & E " " MONITORING " " SURVEILLANCE " " GOVERNANCE " " HUMAN RESOURCE" " HUMAN CAPITAL " " SCALING UP " " REALLOCATE RESOURCES " " STRATEGIES AND PROGRAM" " HIV STRATEG" " PROGRAM IN COUNTRY ACTIVITIES " " STRATEGIC INFORMATION " " PROCUREMENT " " EVIDENCE BASED " " CASE REPORTING " " MEDICAL WORKER" " HEALTH CARE PERSONNEL " " OPERATIONAL RESEARCH " " SUPPORTIVE ENVIRONMENT " " INFORMATION SYSTEM" " WORKFORCE " " INFRASTRUCTUR" " MEDICAL EDUCATION " " CASE NOTIFICATION " " CASE FINDING " " LABORATORY STRENGTHENING " " LABORATORY QUALITY " " LABORATORY NETWORK" " CONTROL SERVICES " " INFECTION CONTROL " " CONTROL PROGRAM" " SCALE UP" " STOP TB STRATEGY " " HEALTH EDUCATION " " CONTINUING EDUCATION " " SUPPLY " " HEALTH MANAGEMENT" " HEALTH POLICY " " MANAGEMENT AND COORDINATION " " ADMINISTRATIVE MANAGEMENT " " MANAGEMENT AND ADMINISTRATION " " COLD CHAIN" " HEALTH PROMOTION " " TECHNICAL ASSISTANCE " " DSS " " DISTRIBUTION SYSTEMS " " SERVICE DELIVERY " " HEALTH FACILIT" " CONSTRUCT" " MEDICAL SCHOOL" "CENTERS OF EXCELLENCE" "NURSE" "DOCTOR" "PHYSICIAN" "MEDICAL LABORATORY SCIENTIST" "SURGEON" "SPECIALIST" "PHARMACIST" "HEALTH LABOR" "LABOR MARKET" "PERSONNEL" "MEDICAL PRACTIONER" "DENTAL PRACTIONER" "TASK SHIFTING"
	Human resources	" INFRASTRUCTUR" " MEDICAL EQUIPMENT" " SURGICAL EQUIPMENT" " HOSPITAL EQUIPMENT" " HOSPITAL EQMT " " BUILDINGS " " HEALTH FACILIT" " CONSTRUCT" " MEDICAL SCHOOL" "CENTERS OF EXCELLENCE" " TRAINING " " CAPACIT" " SKILLED WORKER" " HEALTH WORKER" " SKILLED STAFF " " HEALTH PROFESSIONAL" " HUMAN RESOURCE" " HUMAN CAPITAL " " MEDICAL WORKER" " WORKFORCE " " MEDICAL EDUCATION " " HEALTH EDUCATION " " CONTINUING EDUCATION " " HEALTH MANAGEMENT" " MANAGEMENT AND

Health focus area level	*Program area*	*Keywords*
		COORDINATION " " ADMINISTRATIVE MANAGEMENT " " MANAGEMENT AND ADMINISTRATION " "NURSE" "DOCTOR" "PHYSICIAN" "MIDWIFE" "MIDWIVES" "MEDICAL LABORATORY SCIENTIST" "SURGEON" "SPECIALIST" "PHARMACIST" "HEALTH LABOR" "LABOR MARKET" "PERSONNEL" "MEDICAL PRACTIONER" "DENTAL PRACTIONER" "TASK SHIFTING" " TASK SHARING”
	Pandemic preparedness	" PANDEMIC PREPAREDNESS " " PANDEMIC RESPONSE" " PANDEMIC ALERT" " EPIDEMIC ALERT" " EPIDEMIC RESPONSE" " EPIDEMIC PREPAREDNESS " " OUTBREAK RESPONSE" " OUTBREAK ALERT" " OUTBREAK PREPAREDNESS " " PANDEMIC INFLUENZA " " EPIDEMIOLOGICAL INVESTIGATION" " CONTACT MANAGEMENT " " PREPAREDNESS AND RESPONSE PLAN" " PREPAREDNESS & RESPONSE PLAN" " BIOSAFETY MEASURE" "EARLY WARNING " " HEALTH SECURITY PREPAREDNESS " " HEALTH SECURITY RISK ASSESSMENT " " RAPID RESPONSE STRATEG" " CONTACT TRAC" " PREPAREDNESS AND RESPONSE " " PANDEMIC PLANNING " " INTERNATIONAL HEALTH REGULATION " " IHR "
	Monitoring and evaluation	“ M E “ “ M E “ “ MONITORING “ “ EVALUATION “ “ SURVEILLANCE “
Other infectious diseases	envelope/other	" INFECTIOUS " " COMMUNICABLE " " TRICHURIASIS " " YELLOW FEVER " " WHIPWORM " " TRACHOMA " " SCHISTOSOMIASIS " " BILHARZIA " " SNAIL FEVER " " KAYAYAMA FEVER " " RABIES " " ONCHOCERCIASIS " " RIVER BLINDNESS " " ROBLES DISEASE" " LYMPHATIC FILARIASIS " " ELEPHANTIASIS " " LEISHMANIASIS " " LEISHMANIOSIS " " HOOKWORM " " FOOD BORNE " " FOODBORNE " " ECHINOCOCCOSIS " " HYDATID DISEASE" " HYDATIDOSIS " " DENGUE " " CYSTICERCOSIS " " CHAGAS " " TRYPANOSOMIASIS " " SLEEPING SICKNESS " " ASCARIASIS " " TROPICAL DISEASE" " CHOLERA " " DYSENTERY " " PARASITE DISEASE" " FAO " " NEGLECTED TROPICAL DISEASE" "TYPHOID" " LEPROSY " " BURULI ULCER " " EBOLA" " EBOV " " EVD " " ZIKA " " ZIKV " " GUINEA WORM " " DRACUNCULIASIS " " FILARIASIS " " HEPATITIS E" " ENCEPHALITIS " " VARICELLA" " INFLUENZA" " FLU " " NTD " " HEPATITIS A " " HEPATITIS D " " HEP A " " HEP D " " ROUNDWORM" " RINGWORM" " TAPEWORM" " FLATWORM" " CHIKUNGUNYA " " LASSA " " MERS " " NIPAH " " MARBURG " " SARS "
	Ebola	" EBOLA" " EBOV " " EVD "
	Zika	" ZIKA " " ZIKV "
	Antimicrobial resistance	" ANTIMICROBIAL RESISTAN" " ANTI MICROBIAL RESISTAN" " ANTIBIOTIC RESISTAN" " AMR " "DRUG RESISTAN" " MDR " " XDR " " RESISTANCE TESTING " " DRUG SUSCEPTIBILITY TESTING " " DST " " SECOND LINE "

Table S3.5. Additional health focus area categorizations

Channel	*Allocation criteria*	*Health focus area*
Bilaterals and the EC	CRS purpose code 13030, family planning	Family planning
	CRS purpose code 13020, reproductive health care	Maternal health, non-family planning
	CRS purpose code 12240, basic nutrition	Child and newborn nutrition
	CRS purpose code 12250, infectious disease control and the keywords “child” or “vaccine” present in descriptive variables	Child and newborn vaccines
	CRS purpose code 13040, STD control including HIV/AIDS	HIV/AIDS
	CRS purpose code 12262, malaria control	Malaria, unspecified
	CRS purpose code 12250, infectious disease control and no other keywords present in the descriptive variables	Other infectious diseases
	CRS purpose code 12263, tuberculosis control	Tuberculosis
	CRS purpose code 12230, basic health infrastructure	SWAPs/health system strengthening - other
	CRS purpose code 12281, health personnel development	SWAPs/health system strengthening - Human resources
	CRS purpose code 12281, medical education/training	SWAPs/health system strengthening - Human resources
World Bank IDA and IBRD	Theme code reproductive and maternal health	Reproductive and maternal health, other
	Theme code adolescent health	Reproductive and maternal health, other
	Theme code tuberculosis	Tuberculosis, other
	Theme code child health	Newborn and child health, other
	Theme code HIV/AIDS	HIV/AIDS, other
	Theme code malaria	Malaria, other
	Theme code non-communicable diseases	Non-communicable diseases, other
	Theme code nutrition	Newborn and child health, nutrition
	Theme code neglected tropical diseases	Other infectious diseases, other
	Theme code health system strengthening	SWAPs/health systems strengthening, other
	Theme code health finance	SWAPs/health systems strengthening, other
	Theme code health service delivery	SWAPs/health systems strengthening, other
	Theme code private sector delivery in health	SWAPs/health systems strengthening, other
	Theme code pandemic response	SWAPs/health systems strengthening, pandemic preparedness
UNFPA	Family planning, population and development strategies, population and development, population dynamics<br>Output 02. Integrated sexual and reproductive health services	Family planning
	Reproductive health, maternal and newborn health, young people’s SRH and sexuality education, HIV and STI prevention services, sexual and reproductive health, sexuality education	Maternal health

Channel	*Allocation criteria*	*Health focus area*
	Output 02. Integrated sexual and reproductive health services	
	Output 02. Integrated sexual and reproductive health services	Split equally between family planning and maternal health, unspecified, and HIV other
	HIV and STI prevention services, HIV and AIDS	HIV prevention
	Advocacy, multisector, gender equality and women's empowerment, reproductive rights, program coordination and assistance, adolescents and youth, civil society and rights for all, ending harmful practices, marginalized girls, protection rights.<br>Output 01. Sexual and reproductive health policies for those furthest behind<br>Output 02. Integrated sexual and reproductive health services<br>Output 06. Adolescents and youth skills and capabilities<br>Output 07. Adolescents and youth policies<br>Output 08. Youth leadership and participation<br>Output 12. Elimination of harmful practices	Reproductive and maternal health - other
	Health workforce capacity	Reproductive and maternal health HSS - human resources
	Data and policies, data availability and analysis, data production and dissemination, monitoring and evaluation, national population data system, organizational adaptability, organizational effectiveness and efficiency, population data analysis, population dynamics, program coordination and assistance, program effectiveness, resources management.<br>Output 04. Delivery of sexual and reproductive health commodities<br>Output 05. Accountability for sexual and reproductive health<br>Output 09. Gender equality laws and policies<br>Output 10. Gender and sociocultural norms<br>Output 11. Prevention and addressing of gender-based violence<br>Output 13. Population data systems<br>Output 14. Demographic intelligence<br>OEE1. Improved programming for results<br>OEE2. Optimized management of resources<br>OEE3. United Nationals coordination and coherence<br>OEE4. Communication, resources mobilization and partnerships	Reproductive and maternal health HSS - other
UNICEF	HIV/AIDS results	HIV/AIDS – prevention, treatment, care, PMTCT, OVC, and other
	Nutrition results	Newborn and child health – nutrition
	Health results	Reproductive and maternal health – other<br>Newborn and child health – HSS, vaccines, other
	Ebola (starting in 2014)	Other infectious diseases - Ebola

Channel	*Allocation criteria*	*Health focus area*
	The keyword search was run on budget information for years 2008–2019<br>Program components in budget documents from 1998 to 2007	All program areas under HIV/AIDS and TB
UNAIDS	Assigned in database received through correspondence	HIV/AIDS (care and strengthening, counseling and testing, orphans and vulnerable children, PMTCT, prevention, treatment, other), TB (diagnosis, treatment and other), HSS
GLOBAL FUND	Disease components for malaria, HIV/AIDS, TB, TB/HIV, and other (health systems strengthening)<br>Keyword search on program service delivery areas	All program areas under Malaria, TB, HIV and SWAP/HSS
GAVI	Reproductive, maternal, newborn, child, and adolescent health (divided by 2); Research in human reproduction	Maternal health, unspecified, other non-communicable diseases, maternal newborn child vaccines, health system strengthening
WHO	Nutrition	Child and newborn nutrition
	Vaccine-preventable diseases	Child and newborn vaccines
	Reproductive, maternal, newborn, child, and adolescent health (divided by 2)	Child and newborn health, unspecified
	Aging and health	Maternal, newborn, and child health, unspecified
	HIV/AIDS	HIV/AIDS
	Malaria	Malaria
	Tuberculosis	Tuberculosis
	Mental health and substance abuse	Non-communicable diseases, mental health
	Disabilities and rehabilitation; non-communicable diseases; violence and injuries	Non-communicable diseases, unspecified
	neglected tropical diseases; tropical disease research; outbreak and crisis response (50%); alert and response capacities (50%); polio eradication	Other infectious diseases
	Health system information and evidence; integrated people-centered health services; national health policies, strategies and plans; access to medicines and health technologies and strengthening regulatory capacity; health emergency information and risk assessment (50%); improved access to quality essential health services; reduced number of people suffering financial hardships; improved access to essential medicines, vaccines, diagnostics, and devices for primary health care; determinants of health addressed; risk factors reduced through multisectoral action; healthy settings and health in all policies promoted; strengthen country capacity in data and innovation; strengthen leadership, governance, and advocacy for health; financial, human, and administrative resources managed in an efficient, effective, results-oriented, and transparent manner	SWAps/health system strengthening

Channel	Allocation criteria	Health focus area
	Country health emergency preparedness and the International Health Regulations; health emergency information and risk assessment (50%); emergency operations; emergency core services; outbreak and crisis response (50%); epidemic- and pandemic-prone diseases; alert and response capacities (50%); infectious hazard management; countries prepared for health emergencies; epidemics and pandemics prevented; health emergencies rapidly detected and responded to	SWAps/health system strengthening, pandemic preparedness
	social determinants for health; health and the environment; food safety; antimicrobial resistance; gender, equity, and human rights mainstreaming	Other
	HIV/AIDS and STls; HIV/AIDS, TB, and malaria (33%)	HIV/AIDS, unspecified
PAHO	Tuberculosis; HIV/AIDS, TB, and malaria (33%)	Tuberculosis, unspecified
	HIV/AIDS, TB, and malaria (33%); malaria and other Vector-Borne Diseases (50%);	Malaria, unspecified
	Communicable diseases; Malaria and other vector-borne diseases (50%); neglected tropical and zoonotic diseases; response capacity for communicable diseases; risk factors for communicable diseases; elimination of communicable diseases	Other infectious diseases
	Nutrition; nutrition food safety and food security; malnutrition	Child and newborn nutrition
	Vaccine-preventable diseases	Child and newborn vaccines
	Women's, maternal, newborn, child, and adolescent and adult health	Maternal and child health, unspecified
	Mental health and psychoactive substance use disorders; intersectoral action on mental health; Access to services for NCDs and mental health conditions (50%)	Non-communicable diseases, mental health
	Non-communicable diseases and risk factors; chronic non-communicable diseases; access to services for NCDs and mental health conditions (50%); risk factors for NCDs	Non-communicable diseases, unspecified
	Health systems leadership and governance; human resources for health; social protection and financing; health systems information and evidence; health services; people-centered integrated health services; access to medical products and strengthening regulatory capacity; health governance and financing, national health policies, strategies, and plans; access to comprehensive and quality health services; access to health technologies; strengthened stewardship and governance; increased public financing for health; strengthened financial protection; health promotion and intersectoral action; integrated information systems for health; data, information, knowledge, and	SWAPs/health system strengthening, unspecified

Channel	Allocation criteria	Health focus area
	evidence; research, ethics, and innovation for health; leadership and governance; management and administration	
	Health emergencies preparedness and risk reduction; epidemic and pandemic prevention and control; health emergencies detection and response	SWAP/health system strengthening, pandemic preparedness
	Violence and injuries; disabilities and rehabilitation; antimicrobial resistance; aging and health; gender, equity, human rights, and ethnicity; social determinants of health; health and the environment; strategic communications; management and administration; flexible and learning organization; health throughout the life course; quality care for older people; response capacity for violence and injuries	Other

S3.1.3 Disaggregating preliminary estimates by health focus area

Estimates by health focus area for years in which descriptive data were not available (usually 2024) were obtained by modeling channel-specific DAH per health focus area as a function of time. Out-of-sample validation was used to test the predictive accuracy of a large suite of models, estimating the models using 1990–2010 data and predicting 2011 and 2012. The potential models included fractional multinomial logit regression, OLS regression, autoregressive integrated moving average (ARIMA) models, Epanechnikov kernel-weighted local polynomial smoothing, and multivariable fractional polynomial models. For each model, time was modeled linearly, with splines, and by including lag-dependent variables. Other methodologies considered included modeling health-focus-area-specific DAH as a dollar amount and as a fraction of the channel-specific total DAH. Lastly, models that involved transforming the dependent variable in natural log and logit transformed space were considered. In order to accommodate zero values in the logit transformation, the transformation described in Smithson and Verkuilen were applied.[73] Over 40 models and specifications were evaluated in total.

Each of the potential models and specifications described above was estimated using data from 1990 through 2010, and then the estimated model was used to predict DAH by health focus area for 2011 and 2012. Since we have DAH estimates for 2011 and 2012, we compared the modeled estimates and the observed estimates and calculated average percentage deviation and average total absolute deviation for each model and specification across all the channels and health focus areas. A variant of the Epanechnikov kernel-weighted local polynomial smoothing had the smallest average percentage deviations and average total absolute error. In this model and specification, health focus area-specific DAH fractions were independently estimated at the channel level after they were logit transformed. Time was the only independent variable included in the model. The health focus area-specific DAH estimates were adjusted so the sum of the channel’s health focus area disbursements totaled channel-specific DAH envelope. Our preferred model, the Epanechnikov kernel-weighted local polynomial smoothing, minimized both the average percentage deviation and the total absolute error out of sample, predicting two years ahead. See Dieleman et al. for a table that demonstrates the performance of four models, each with their optimal specification (as determined by the out-of-sample average percentage deviation and total absolute error).[1]

S3.2 Tracking development assistance for health from bilateral aid agencies and the European Commission

OECD-DAC maintains two databases on aid flows: 1) the DAC annual aggregates database, which provides summaries of the total volume of flows from different donor countries and institutions, and 2) the CRS, which contains project- or activity-level data.[3] We used the DAC databases to track health ODA from 32 OECD-DAC

members (Austria, Australia, Belgium, Canada, Czechia, Denmark, Estonia, Finland, France, Germany, Greece, Hungary, Iceland, Ireland, Italy, Japan, Luxembourg, the Netherlands, New Zealand, Norway, Poland, Portugal, Slovak Republic, Slovenia, South Korea, Spain, Sweden, Switzerland, the United Kingdom, the United States, and the EC), and one DAC Participant country, United Arab Emirates, for the years 1990 to 2024. Observed data for the DAC members were available from 1990 to 2023, and observed data for the United Arab Emirates were available from 2009 to 2023. United Arab Emirates bilateral health ODA from 1990 to 2008 was obtained through personal correspondence. We determined the observed 2019 data for the United States was incomplete after consulting several USAID data tools and other data experts in the field. Therefore, we imputed 2019 and predicted 2024 for the United States.

These two DAC databases track the following types of resource flows:

Official development assistance (ODA), defined as "flows of official financing administered with the promotion of the economic development and welfare of developing countries as the main objective"[2] is tracked from its 31 members.The CRS also now includes some private ODA, such as that funded by the Gates Foundation and the Global Fund, as well as assistance from a number of non-DAC countries such as the United Arab Emirates and Kuwait.

ODA includes:

• Bilateral ODA, which is given directly by DAC members as aid to recipient governments, core contributions to NGOs and public-private partnerships, and earmarked funding to international organizations.

• Multilateral ODA, which includes core contributions to multilateral agencies such as WHO, UNFPA, the Global Fund, Gavi, UNAIDS, UNICEF, PAHO, the World Bank, and other regional development banks. Only regular budgetary contributions to these institutions can be reported to the OECD-DAC; hence, extrabudgetary funds, including earmarked contributions that donors can report as bilateral ODA, are not included as multilateral ODA. Only 70% of core contributions to WHO can be counted as multilateral ODA.

a. Official development finance (ODF), which includes grants and loans made by multilateral agencies.

b. Other official flows (OOF), which refers to transactions that "do not meet the conditions for eligibility as Official Development Assistance or Official Aid, either because they are not primarily aimed at development, or because they have a Grant Element of less than 25 percent."

The DAC aggregate tables include all multilateral development banks, the Global Fund, operational activities of UN agencies and funds, and a few other multilateral agencies. The project-level data in the CRS cover a smaller subset of multilateral institutions, including UNAIDS, UNFPA, UNICEF, public-private partnerships including Gavi and the Global Fund, some development banks, and the Gates Foundation, but do not reflect the core-funded operational activities of WHO prior to 2009, disbursements by Gavi prior to 2007 and the Gates Foundation prior to 2009, or all loans from the World Bank.

This research utilized the CRS as the principal source for tracking bilateral DAH. This is because the DAC aggregate tables do not report detailed project-level information about the recipient country and health focus area. The OECD sector codes for general health (121), basic health (122), non-communicable disease (123), and population programs (130) were used to identify health flows in the CRS. Only ODA-related flows are used in our analysis, including OECD flow codes corresponding to ODA grants (11), ODA loans (13), and equity investment (19). In addition, we included select projects with OECD sector code for emergency aid (720) to capture additional funding for Ebola,Zika, Cholera, and COVID-19 outbreaks. Projects with purpose code emergency food aid (72040) were excluded, and the remaining project descriptive variables were parsed for the keywords "EBOLA" , "EBOV", " EBV", "ZIKA", "ZIKV", "CHOLERA", "CHOLERAE", "COLERA", "COVID", "CORONAVIRUS", "SARS COV 2", "SEVERE ACUTE RESPIRATORY SYNDROME", and "NCOV" to identify emergency aid projects targeting these diseases. Based on our definition of DAH, we also excluded any remaining projects containing any of the following keywords: " WATER " " SANITATION " " FOOD " " HUNGER " " EARTHQUAKE " "

HURRICANE " " TERREMOTO " " HURACAN " “FLOOD” " FAMILY CARE STRUCTURES " “VIOLENCE”, “SOCIO ECONOMIC”.

To avoid double-counting, all identifiable earmarked commitments and disbursements between one disbursing agency to another were tagged and removed before calculating final DAH estimates. Income statements, expenditure statements, and keyword searches in the descriptive project fields were used to identify potential sources of double-counting.

In multilateral agencies, public-private partnerships, development banks, and foundations, when expenditure by recipient agency information was available, double counting was tagged and dropped in the disbursing agency from whom the resources was transferred. In the absence of detailed expenditure data in the disbursing agency that transferred resources, the income statement of the receiving disbursing agency that reflected the disbursement was used to flag the disbursement in the initial disbursing agency. Preliminary estimates were generated based on the annual disbursed envelope exclusive of resources flagged as transfers to other agencies.

Channel codes in the CRS data were used to track DAH to international and donor-country-based non-governmental organizations. The names of NGOs that were captured in IHME’s NGO data (as detailed in section 3.7 titled “Tracking non-governmental organizations”) were searched for in the CRS descriptive variables and tagged as double-counting. Research funds for HIV/AIDS channeled by the US government through the National Institutes for Health (NIH) were also removed from the total since they do not meet the definition of DAH as contributions from institutions whose primary purpose is development assistance. Official development finance (ODF) from the CRS was not counted because these expenditures were included elsewhere, either in the analysis of multilateral institutions relevant to the study or in the assessment of health spending by the Gates Foundation, the data for which were obtained via correspondence and from their annual reports, audited financial statements, and project databases. To avoid double-counting, only health assistance flows from multilateral institutions to low- and middle-income countries were counted, and not transfers to multilateral institutions. Also, for regional projects, the disbursements are split among all countries in the specified OECD region. For example, a project allocated to recipient “North of Sahara, regional” would have its disbursements split equally between all the countries in the corresponding OECD region: Algeria, Egypt, Libya, Morocco, and Tunisia.

Allocation of funding to health focus areas was assigned as described in the section “DISAGGREGATING BY HEALTH FOCUS AREA” based on a keyword search of five descriptive variables in the CRS: project title, short description, long description, channel name, and channel reported name. Additional adjustments were made based on CRS purpose codes, as detailed in Table S3.5, to ensure that the specified purpose corresponded to the highest-weighted **bilateral** health focus area.

S3.2.1 Estimating disbursements for the 31 channels and the EC

Both the DAC tables and the CRS rely on information reported by DAC members and other institutions to the OECD-DAC. Hence, the quality of the data varies considerably over time and across donors. Three variables were used to estimate yearly donor disbursements: CRS commitments, CRS disbursements, and DAC commitments. There were two main challenges in using the data from the CRS for this research:

1. Underreporting of aid activity to the CRS compared to what is reported to the DAC, and

2. Underreporting of disbursement data to the CRS compared to commitment data reported to the CRS.

These issues are highlighted in Figure S3.1. Methods developed to account for both these challenges are discussed below. Details on how we estimated the cost of providing technical assistance and program support for these institutions are highlighted below in the section titled calculating the “Technical assistance and program support component of development assistance for health from loan- and grant-making channels of assistance.”

To address these two challenges, we determined a cutoff point for each channel. We defined this channel-specific cutoff year as when the ratio of total CRS disbursements to commitments was greater than 50% and did not drop subsequently below 30%. Figure S3.2 below shows each donor's CRS disbursement to commitment ratio in green, and the estimated cutoff year is marked with a vertical red line. For years after the cutoff year, DAH is measured using the unadjusted disbursement data. For the time prior to the cutoff year, it was determined that the disbursement data are not of high enough quality, and adjusted commitments were used instead.

Two adjustments were made to commitments to estimate disbursements before each donor-specific cutoff point:

I. The first adjustment addressed underreporting of aid activity to the CRS (relative to the DAC). To address this challenge, all CRS commitments for the health sector were adjusted upward using the DAC commitment to CRS commitment coverage ratio. The coverage ratio of the CRS was well below 10% before 1996 but has improved steadily over time.

II. The second adjustment addressed underreporting of disbursements data to the CRS (relative to commitments reported to the CRS). To address this challenge, we pooled completed projects in the CRS that have disbursement data for each channel and computed yearly project disbursement rates (the fraction of total commitments disbursed for each year of a project) and overall project disbursement rates (the fraction of total commitments disbursed over the life of each project) by project length. Yearly disbursement schedules were calculated for projects with lengths of one, two, three, four, five, and six years. When an observed project length was more than six years, all expenditure after the sixth year was aggregated and assumed to be expended in the sixth year. This does not happen often. Yearly disbursement rates were the median of these shares, averaged across projects for every donor in each project year. The sum of these averages equals one, so that all the disbursements were expended over the lifetime of a project. We also adjusted donor disbursement schedules by the ratio of disbursements to commitments in the CRS to reflect the fact that not all commitments are typically disbursed. The product of these donor-specific yearly disbursement rates, the donor-specific overall disbursement rates, and the donor-specific disbursement to commitment ratios produced the donor-specific disbursement schedules. The donor-specific disbursement schedules were applied to project-level DAC-adjusted commitments reported in the CRS. Figure S3.3 shows the yearly disbursement rates and overall disbursement rates for projects with one- to six-year lifespans for each of the 24 member countries and the EC.

Lastly, to address the challenge of underreporting of aid activity to the CRS compared to the DAC for all years, the difference between each donor's aggregate (adjusted) DAC health commitments and CRS health disbursements was added to each donor's yearly DAH. Since only aggregate commitments are reported to the DAC, several adjustments were made, based on more detailed CRS data:

I. First, each donor's yearly median project length was calculated by applying the donor-specific disbursement schedules described above to CRS projects that had disbursement in order to get adjusted DAC commitments.

II. Commitments for projects that have not opened yet were then subtracted, based on the open date reporting in the CRS. This ensured that future disbursements were not captured.

III. The donor-specific ratio of disbursements to commitments was applied to adjusted DAC commitments to account for the fact that not all commitments are usually disbursed.

IV. Lastly, these DAC-adjusted commitments were compared to CRS disbursements, inclusive of transfers that were later dropped as double-counting.

In addition to tracking disbursements from the EC, gross disbursements from the DAC were used to compile data on the sources of funding for the EC.

Figure S3.1 Comparing CRS commitments, CRS disbursements, and DAC commitments

This figure compares commitments and disbursements from the Creditor Reporting System (CRS) and Development Assistance Committee (DAC) databases of the Development Assistance Committee of the Organisation for

Economic Co-operation and Development (OECD-DAC) from 1990 to 2020. CRS disbursements are usually underreported when compared to both CRS and DAC commitments data, especially in earlier years. Because of this gap between CRS and DAC, CRS disbursements data were adjusted to fit DAC commitments data. The drop in 2019 across all commitments and disbursements is due to incomplete USA bilateral data for that year.

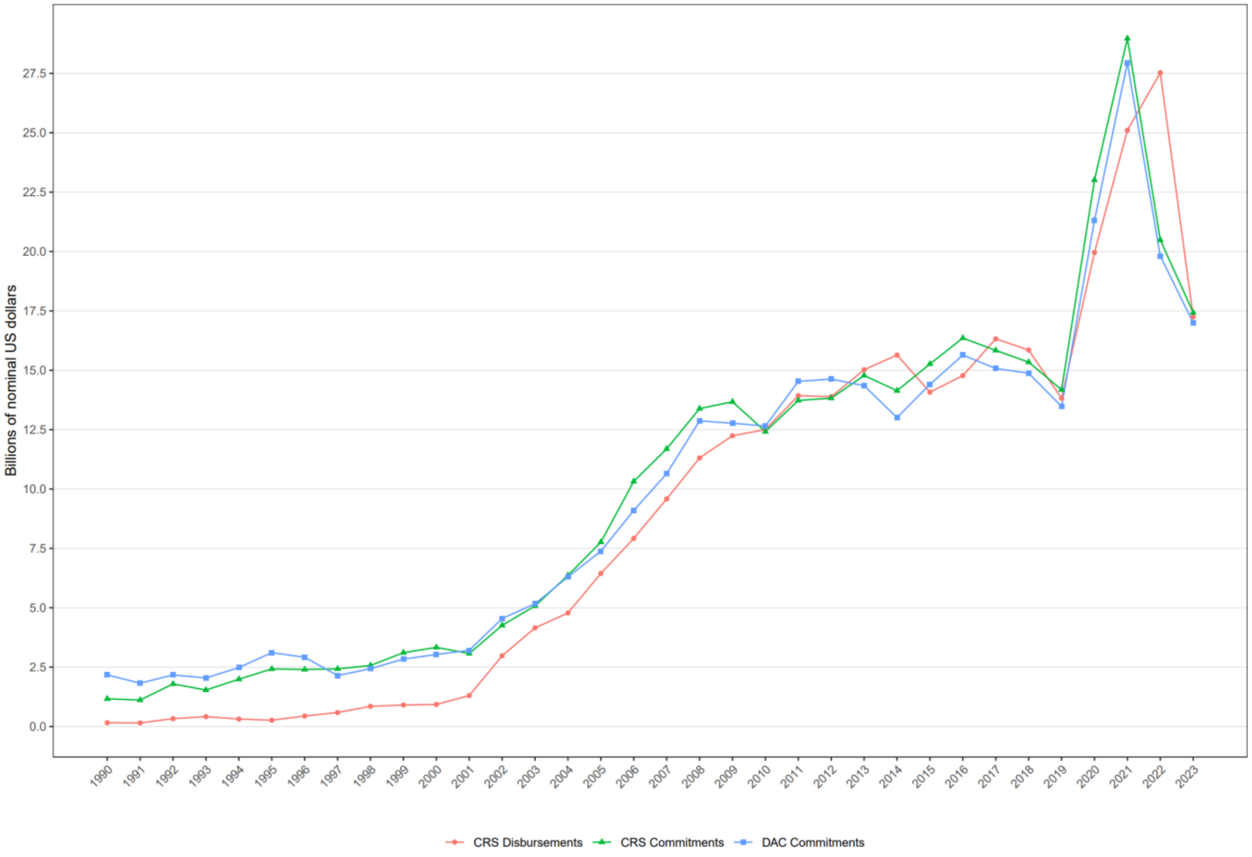

Source: OECD-DAC and OECD Creditor Reporting System

Figure S3.2 CRS disbursement to commitment ratio and cutoff points by donor agency

This figure shows the channel-specific cutoff year. Before this year, we adjust CRS commitments using disbursement schedules. After this cutoff, we rely on CRS-reported disbursements. The total CRS disbursements to commitments ratio is in black, and the cutoff year is marked with a vertical red line. The cutoff year is determined to be when the ratio goes above 50% and does not fall back below 30%. The vertical axis represents the CRS disbursement to commitment ratio as a percentage. ARE = United Arab Emirates, AUS = Australia, AUT = Austria, BEL = Belgium, CAN = Canada, CHE = Switzerland, CZE = Czechia, DEU = Germany, DNK = Denmark, EC = European Commission, ESP = Spain, EST = Estonia, FIN = Finland, FRA = France, GBR = Great Britain, GRC = Greece, HUN = Hungary, IRL = Ireland, ISL = Iceland, ITA = Italy, JPN = Japan, KOR = South Korea, LTU = Lithuania, LUX = Luxembourg, NLD = the Netherlands, NOR = Norway, NZL = New Zealand, POL = Poland, PRT = Portugal, SVK = Slovakia, SVN = Slovenia, SWE = Sweden, USA = United States of America

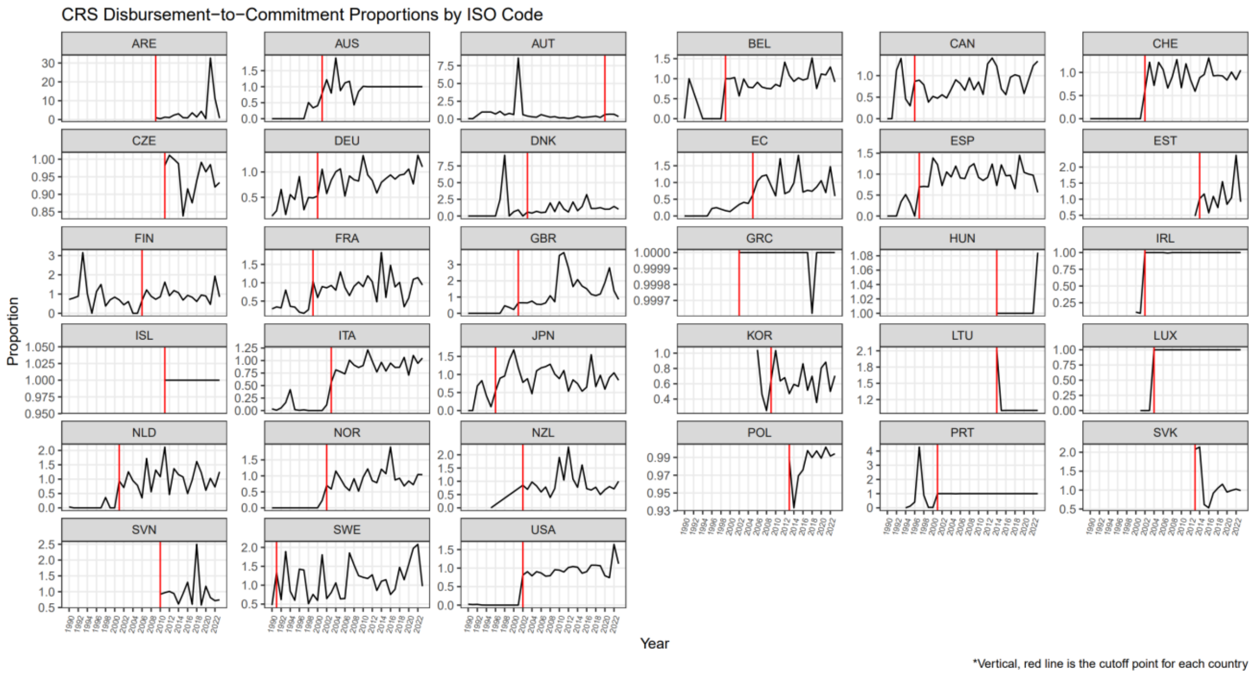

Source: OECD Creditor Reporting System

Box 3.3. EXAMPLE. Australia's CRS disbursement to commitment ratio and cutoff year

The black line shows the ratio of Australia's disbursements to commitments, as reported in the CRS. Prior to 2001, the ratio was always below 50%. In 2001, the ratio rose above 50%; it did not fall below 30% in subsequent years, thereby defining 2001 as the cutoff year. Thus, for Australia, before 2001, DAH is based on adjusted CRS commitment data. These data are adjusted using disbursements schedules (Figure S3.3) and data from the DAC. After 2001, Australia's DAH is based on the disbursements reported in the CRS.

Figure S3.3 One- to six-year disbursement schedules for bilateral channels

This figure shows the estimated disbursement schedules for bilateral channels. Before the channel-specific cutoff year, we rely on commitment data to inform our estimates of DAH. Commitment data are adjusted to reflect disbursements over time using schedules estimated from projects in the CRS that have both commitment and disbursement data. The vertical axis represents the percentage of the commitment disbursed. ARE = United Arab Emirates, AUS = Australia, AUT = Austria, BEL = Belgium, CAN = Canada, CHE = Switzerland, CZE = Czechia, DEU = Germany, DNK = Denmark, EC = European Commission, ESP = Spain, EST = Estonia, FIN = Finland, FRA = France, GBR = Great Britain, GRC = Greece, HUN = Hungary, IRL = Ireland, ISL = Iceland, ITA = Italy, JPN = Japan, KOR = South Korea, LTU = Lithuania, LUX = Luxembourg, NLD = the Netherlands, NOR = Norway, NZL = New Zealand, POL = Poland, PRT = Portugal, SVK = Slovakia, SVN = Slovenia, SWE = Sweden, USA = United States of America

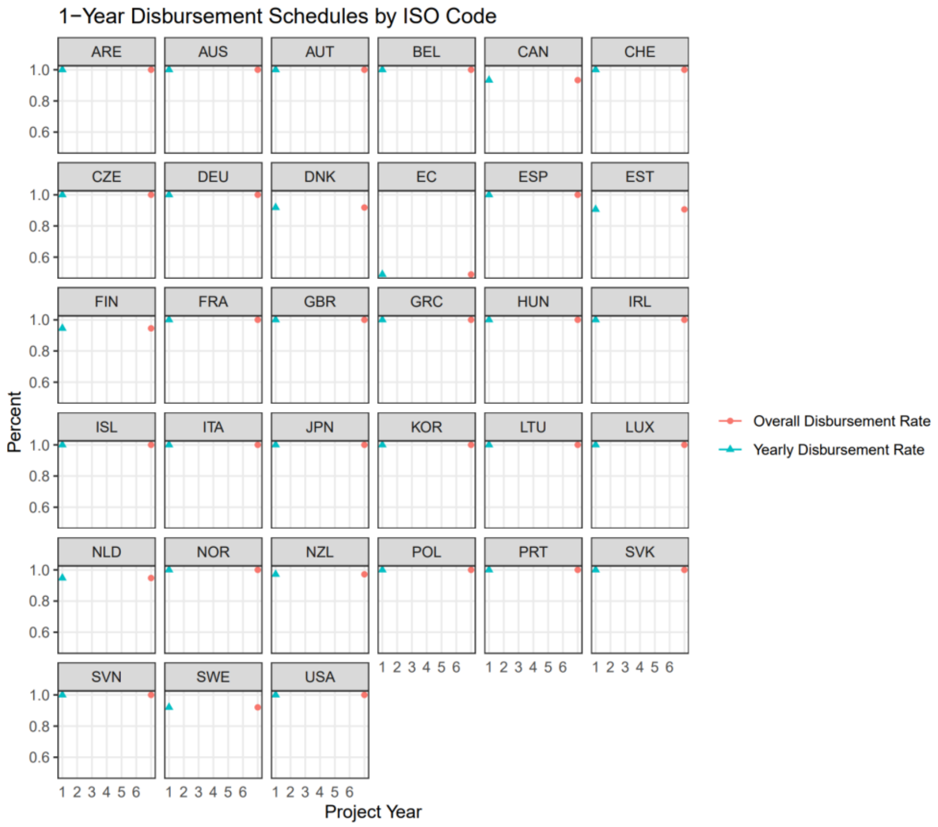
1−Year Disbursement Schedules by ISO Code
ARE
AUS
AUT
BEL
CAN
CHE
CZE
DEU
DNK
EC
ESP
EST
FIN
FRA
GBR
GRC
HUN
IRL
ISL
ITA
JPN
KOR
LTU
LUX
NLD
NOR
NZL
POL
PRT
SVK
SVN
SWE
USA
Percent
Project Year
Overall Disbursement Rate
Yearly Disbursement Rate

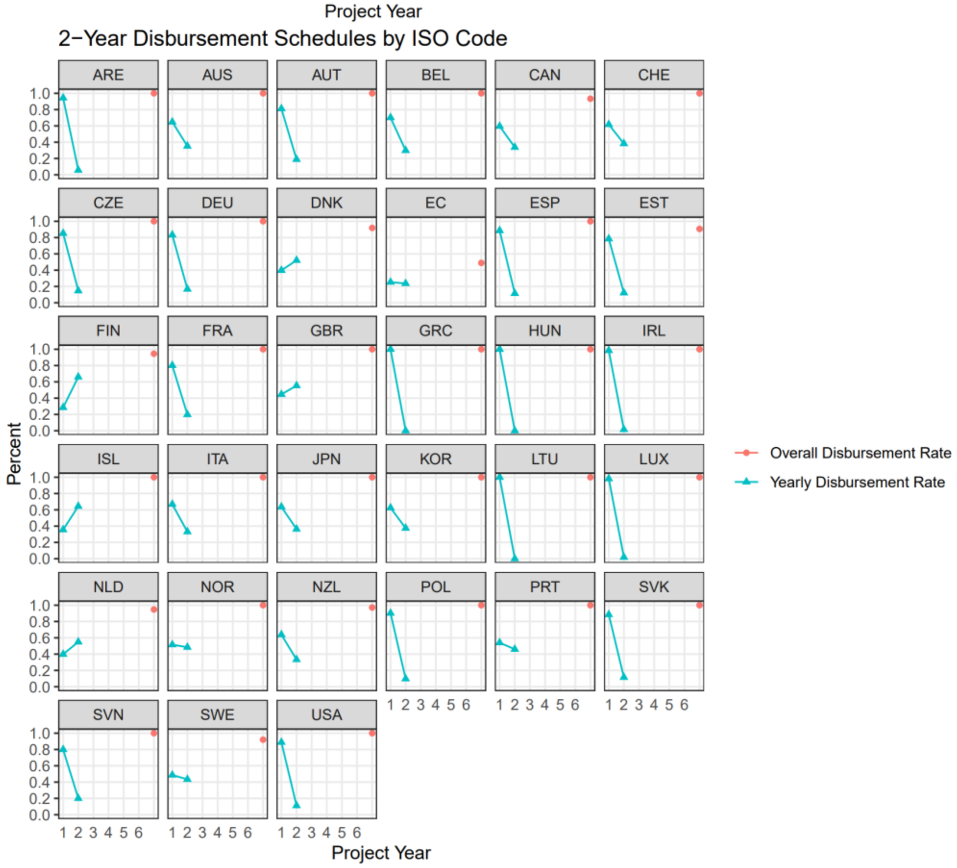
2−Year Disbursement Schedules by ISO Code
ARE
AUS
AUT
BEL
CAN
CHE
CZE
DEU
DNK
EC
ESP
EST
FIN
FRA
GBR
GRC
HUN
IRL
ISL
ITA
JPN
KOR
LTU
LUX
NLD
NOR
NZL
POL
PRT
SVK
SVN
SWE
USA
Percent
Project Year
Overall Disbursement Rate
Yearly Disbursement Rate

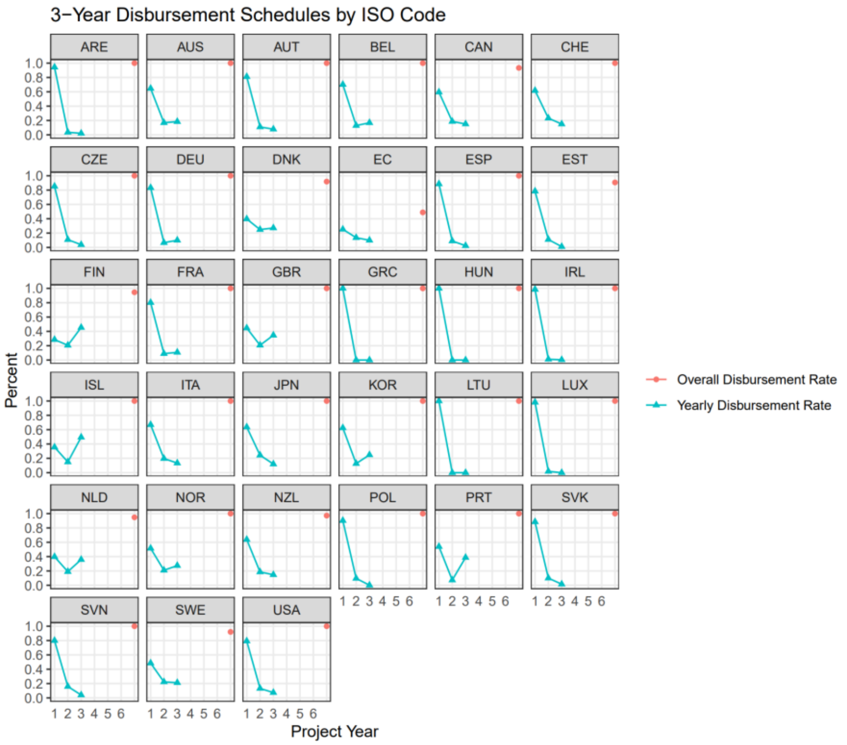
3−Year Disbursement Schedules by ISO Code
ARE
AUS
AUT
BEL
CAN
CHE
CZE
DEU
DNK
EC
ESP
EST
FIN
FRA
GBR
GRC
HUN
IRL
ISL
ITA
JPN
KOR
LTU
LUX
NLD
NOR
NZL
POL
PRT
SVK
SVN
SWE
USA
Percent
1.0
0.8
0.6
0.4
0.2
0.0
1 2 3 4 5 6
Project Year
Overall Disbursement Rate
Yearly Disbursement Rate

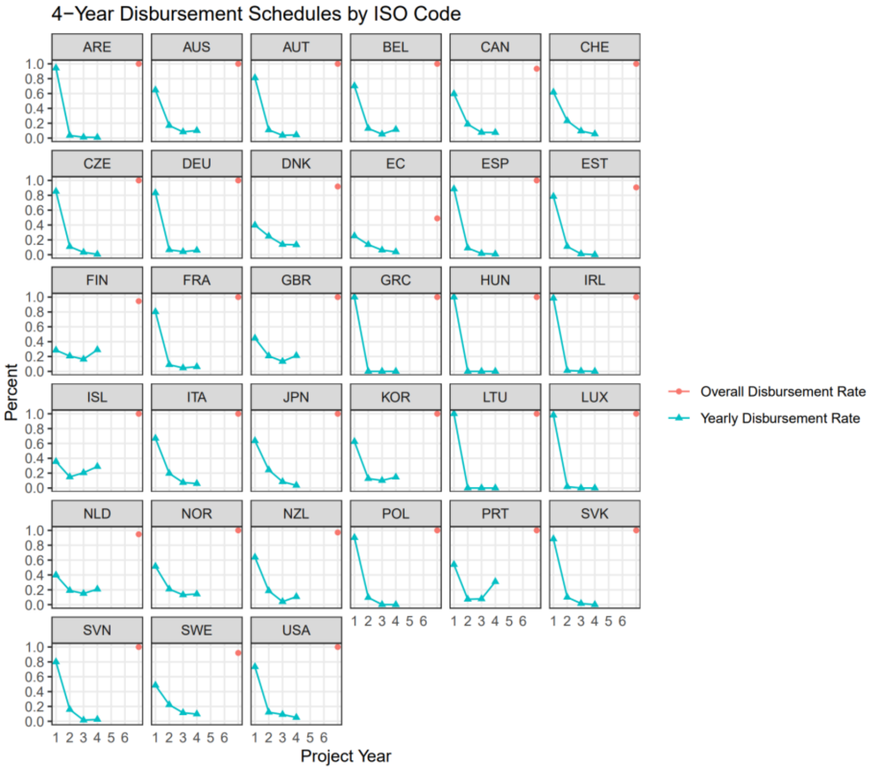
4−Year Disbursement Schedules by ISO Code
ARE
AUS
AUT
BEL
CAN
CHE
CZE
DEU
DNK
EC
ESP
EST
FIN
FRA
GBR
GRC
HUN
IRL
ISL
ITA
JPN
KOR
LTU
LUX
NLD
NOR
NZL
POL
PRT
SVK
SVN
SWE
USA
Percent
1.0
0.8
0.6
0.4
0.2
0.0
1 2 3 4 5 6
Project Year
Overall Disbursement Rate
Yearly Disbursement Rate

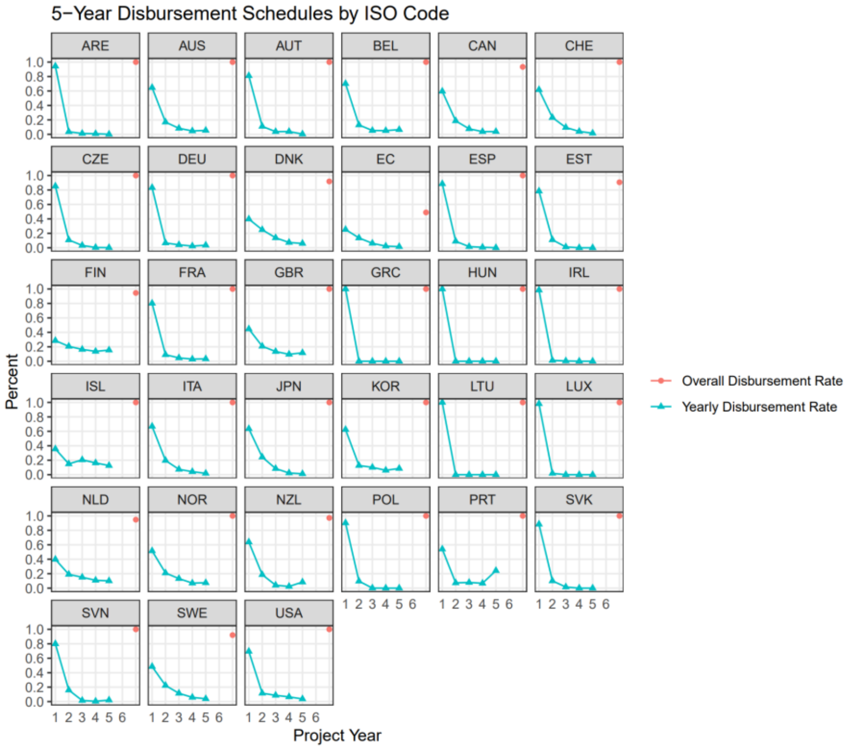
5−Year Disbursement Schedules by ISO Code
ARE
AUS
AUT
BEL
CAN
CHE
CZE
DEU
DNK
EC
ESP
EST
FIN
FRA
GBR
GRC
HUN
IRL
ISL
ITA
JPN
KOR
LTU
LUX
NLD
NOR
NZL
POL
PRT
SVK
SVN
SWE
USA
Percent
1.0
0.8
0.6
0.4
0.2
0.0
1 2 3 4 5 6
Project Year
Overall Disbursement Rate
Yearly Disbursement Rate

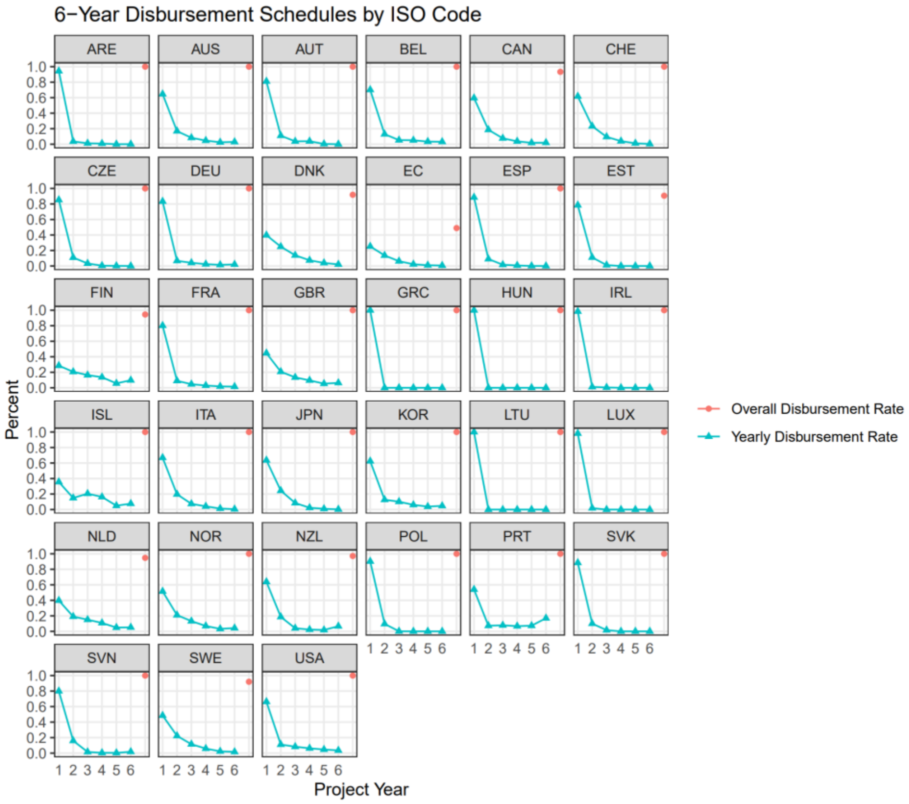
6−Year Disbursement Schedules by ISO Code
ARE
AUS
AUT
BEL
CAN
CHE
CZE
DEU
DNK
EC
ESP
EST
FIN
FRA
GBR
GRC
HUN
IRL
ISL
ITA
JPN
KOR
LTU
LUX
NLD
NOR
NZL
POL
PRT
SVK
SVN
SWE
USA
Percent
1.0
0.8
0.6
0.4
0.2
0.0
1 2 3 4 5 6
Project Year
Overall Disbursement Rate
Yearly Disbursement Rate

Box 3.4. EXAMPLE. Australia's one- to six-year disbursement schedules

To estimate disbursements using commitment data, we rely on disbursement schedules derived from CRS data that include both commitments and disbursements. Disbursement schedules are specific for each channel and the length of a project. These schedules also take into consideration the average amount of commitments for each channel that lead to disbursements. Across all Australian projects in the CRS with complete disbursements data, Australia disbursed 100% of the funds that it committed, as shown by the solid red dot on the right-hand side of the Australia panel (upper left corner of the first panel of Figure S3.3). In projects with a length of one year, Australia disbursed 100% of the funds that it committed in that year. For two-year projects, Australia disbursed 60% of total disbursements in year one and 40% of total disbursements in year two. In projects with lengths of three years, Australia disbursed about 60% of total disbursements in year one and about 20% of total disbursements in years two and three. This is estimated for projects ranging from one to six years. The disbursement schedules were applied to commitment data from the CRS to estimate disbursements for years prior to the cutoff year, which is 2001 for Australia.

To predict DAH for the recent years not reported in the CRS, budget data were extracted from a variety of sources. These data are listed in Table S3.3. Global health budgetary data were utilized whenever possible, but these detailed data were available as a complete time series only for Australia, the United States, and the United Kingdom. For all other bilateral channels, general ODA budgets were used. To predict DAH for 2024 for 32 bilateral agencies the percentage change in budgeted global health spending or budgeted ODA between 2023 and 2024 was applied to the 2023 observed DAH estimate. Figure S3.4 plots the budget ratio for each bilateral channel. Budget data for the EC were inconsistent and did not match the disbursement series. Instead, DAH for 2023 was estimated based on trends in DAH for EC member countries. A weighted average was applied to the percentage change in DAH from 2020–2023 for all EC member countries. The weighting was based on each country's total national contributions to the EC. These data were collected from the EC's financial statements.[74] The weighted average was then applied to the EC's 2023 DAH to forecast 2024.

Figure S3.4 DAH as a percentage of corresponding budget data by bilateral agency

This figure shows the trend of the ratio of DAH measured as a share of budget data. Green dots indicate that a donor provided global-health-specific budget data, so in these cases the denominator is all global-health-specific budgeted data. The numerator is estimated DAH. Red dots indicate that a donor did not have global-health-specific budget data, so overall ODA budget data were used in calculating the DAH to budget ratios. The vertical axis represents estimated DAH as a fraction of corresponding budget data. ARE = United Arab Emirates, AUS = Australia, AUT = Austria, BEL = Belgium, CAN = Canada, CHE = Switzerland, DEU = Germany, DNK = Denmark, ESP = Spain, FIN = Finland, FRA = France, GBR = Great Britain, GRC = Greece, IRL = Ireland, ITA = Italy, JPN = Japan, KOR = South Korea, LUX = Luxembourg, NLD = the Netherlands, NOR = Norway, NZL = New Zealand, PRT = Portugal, SWE = Sweden, USA = United States of America.

Source: IHME DAH Database (2023) and corresponding bilateral ODA/DAH budget documents outlined in Table S3.1 and 3.3.

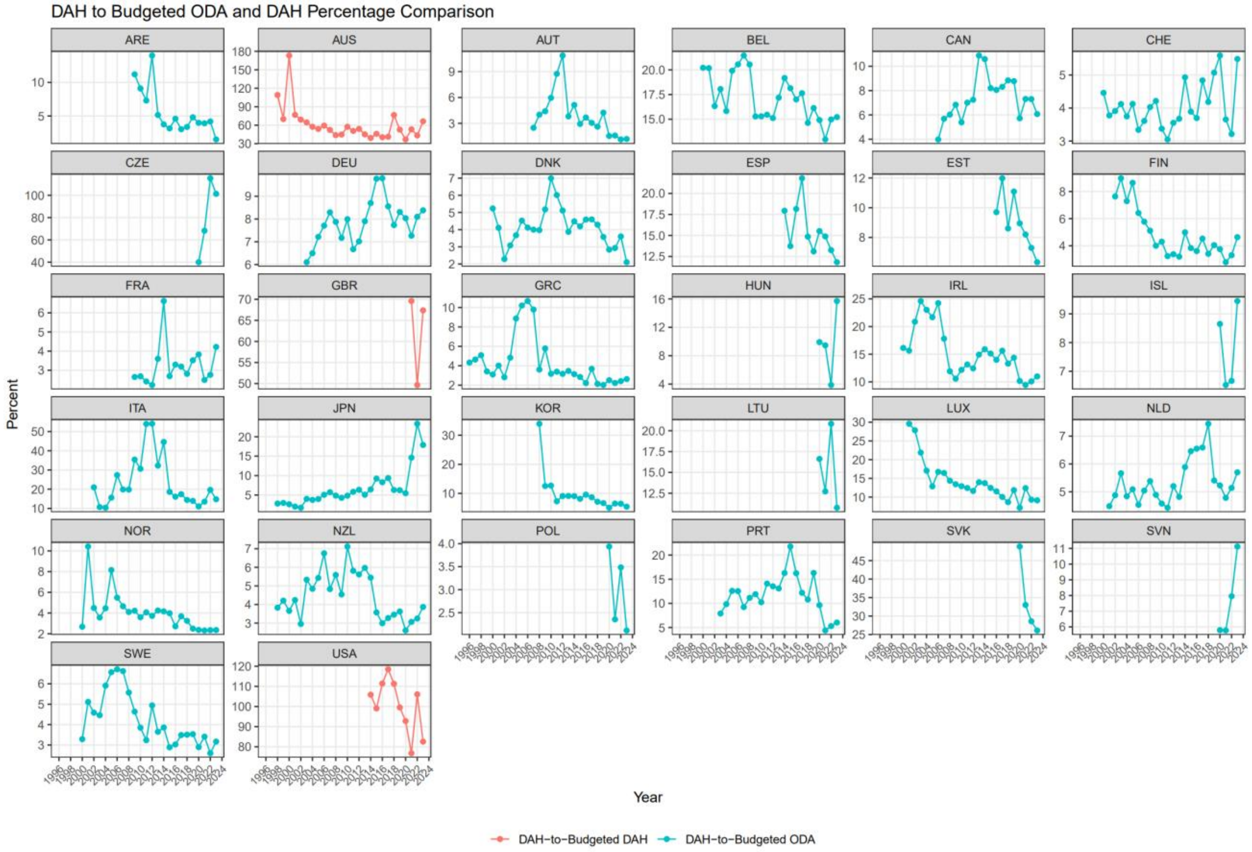

To supplement our estimates of development assistance for health to HIV/AIDS and malaria program areas for the United States, we used additional available data from the President's Emergency Plan for AIDS Relief (PEPFAR) and the President's Malaria Initiative (PMI). We downloaded data on all planned funding by PEPFAR by recipient country, year, and program area from 2004 to 2023.[75] All PEPFAR projects were assigned to our eight HIV/AIDS program areas using PEPFAR financial classification codes, splitting out overhead costs equally to all other program areas. We then created country-year-specific HIV/AIDS program area fractions out of total annual HIV/AIDS DAH, which we applied to all United States HIV/AIDS projects in the CRS from 2004 to 2023 by country-year. To inform malaria funding by program areas, we downloaded the most recently available malaria funding tables from malaria operational plans for all countries and years.[76] We used a keyword search to assign each line item in these tables to our eight malaria program areas, and then created fractions for the malaria program areas out of the total annual malaria DAH specific to each country-year. These fractions were applied to all United States malaria projects in the CRS from 2006 to 2018 by country-year.

Figure S3.5 Malaria DAH to program areas as assigned by keyword search and PMI reports

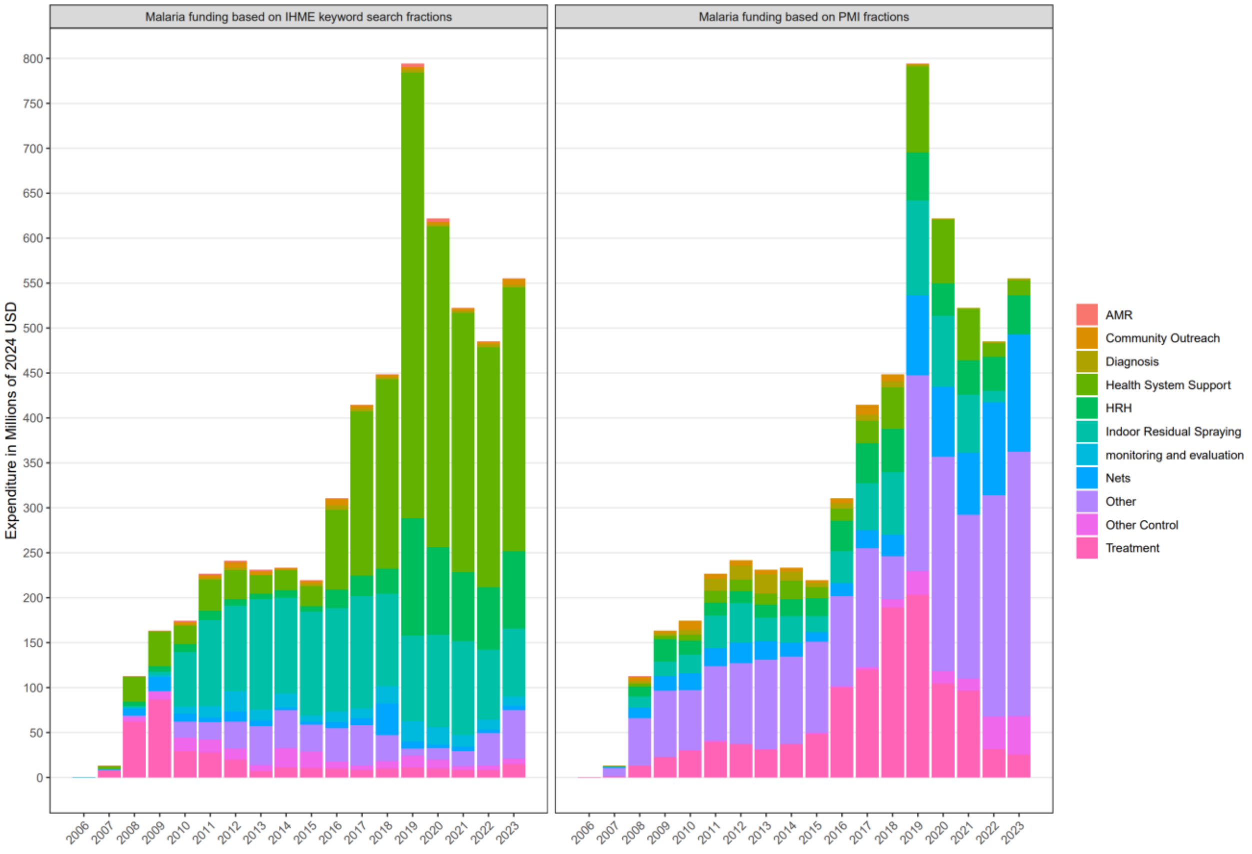

Source: IHME DAH Database (2023) and PMI malaria operational plans

This figure outlines the assignment of funding to malaria program areas for United States projects from the OECD CRS from 2006 onward. The figure on the left shows how malaria funding is broken out based on keyword search. The figure on the right shows the breakdown of funding to malaria program areas based on PMI malaria funding tables.

S3.3 Tracking development assistance for health from the development banks

The World Bank project-level health disbursement data for 1990 through December 2021 were obtained through correspondence with Miyuki T. Parris.[77] In 2016, the World Bank underwent a recoding process for its disbursements. This recoding affected health disbursements; however, the recoding was not completed for projects with disbursements prior to 2001. To create a comparable dataset, adjustments had to be made. Regression analysis to predict health disbursements was explored; however, in the end, the average percentage change between project-level health disbursements before and after recoding was used to adjust health disbursements prior to 2001. It was observed that on average, between 2001 and 2005 (inclusive) the recoding process decreased health disbursements by 0.22%. This number was used to adjust all project-level health disbursements prior to 2001.[78] Health disbursements included all health projects as well as other sector projects with a health sector code. Disbursements for years after 2021 were estimated using the data reported by the World Bank to IATI.[111] In addition, data were collected from the online database to fill in descriptive information for loans from the two arms of the World Bank: the International Development Association (IDA) and the International Bank for Reconstruction and Development (IBRD).[79] Along with keyword searches, health theme codes were used to allocate disbursements by health focus

area. The online database contains up to five sector codes and five theme codes that can be assigned to each project. Sector codes represent economic, political, and social subdivisions, while theme codes represent the goals or objectives of World Bank activities. Since theme codes are missing in later years of project data, we web-scraped them from each project's page on the World Bank website. The codes relating to health are summarized in Table S3.6. Emergency recovery loans were excluded since they do not fit the definition of DAH.

Table S3.6 World Bank's health sector and theme codes[101,102]

Health sector codes<br>*Sector codes represent economic, political, or social subdivisions within society. World Bank projects are classified by up to five sectors.*	*Health theme codes*<br>*Theme codes represent the goals or objectives of World Bank activities.*
Historic (prior to 2001):<br>***(1)** Basic health*<br>***(2)** Other population health and nutrition*<br>***(3)** Targeted health*<br>***(4)** Primary health, including reproductive health, child health, and health promotion*<br><br>*Current (as of 2001):*<br>*(1) Health*<br>*(2) Health facilities and construction*<br>*(3) Public administration – health*	Current:<br>(1) HIV/AIDS<br>(2) Malaria<br>(3) Tuberculosis<br>(4) Neglected tropical diseases<br>(5) Non-communicable diseases<br>(6) Health system strengthening<br>(7) Health service delivery<br>(8) Health finance<br>(9) Private-sector delivery in health<br>(10) Reproductive and maternal health<br>(11) Adolescent health<br>(12) Child health

Data on yearly government contributions were obtained from the DAC statistics to disaggregate IDA flows by source. Because China does not report to DAC, we generated contribution estimates using replenishment data. We split the three-year replenishment amount over the three years of the 16th-19th replenishments, to obtain China's contributions to the World Bank from 2008 to 2018. We validated our use of replenishment data by extracting China's and other countries' contributions to the World Bank International Development Association (IDA) 16th, 17th, and 18th replenishment and comparing the number with the contribution we extracted from the OECD Creditor's Reporting System (CRS).[80] The trend of contribution from major donors was similar, and we therefore used China's replenishment contribution as a proxy of the contribution to World Bank IDA. We further disaggregated the DAH contribution to World Bank IDA from China by multiplying China's contributing proportion over World Bank IDA's total health envelope.

Figure S3.6 Comparison of World Bank IDA Replenishment and DAC

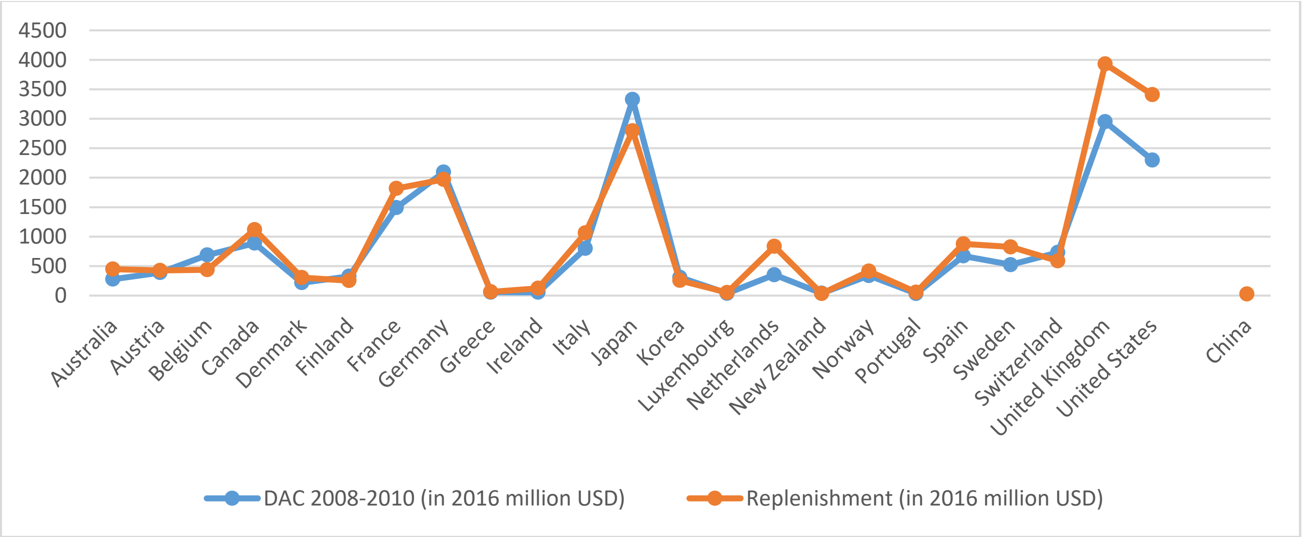

Panel a. Comparison of World Bank International Development Association 16th Replenishment

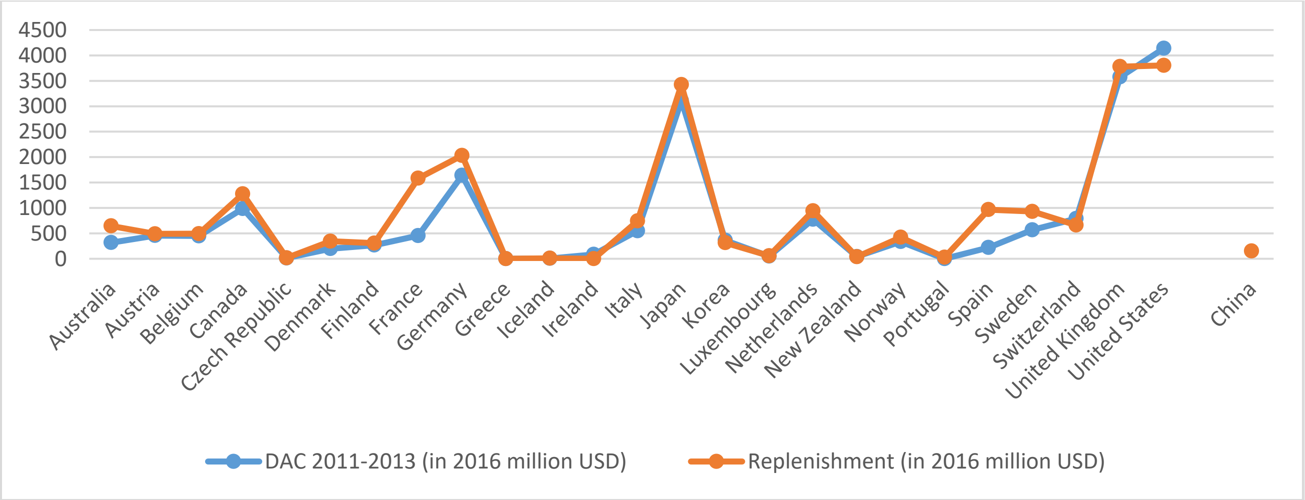

Panel b. Comparison of World Bank International Development Association 17th Replenishment

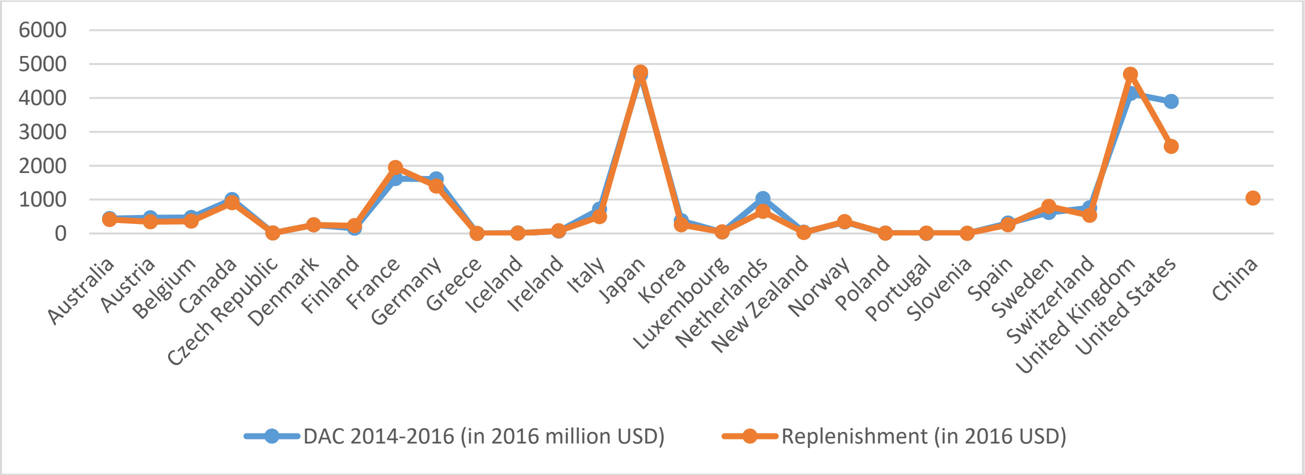

Panel c. Comparison of World Bank International Development Association 18th Replenishment

The three figures show the comparison of country commitment to World Bank IDA replenishment rounds, and yearly government contributions reported to DAC statistics. The orange line shows the country commitment to each IDA replenishment round. The blue line shows the sum of yearly reported contribution during the three-year replenishment round. Panel a is contributions and commitments from replenishment round 16; panel b is contributions and commitments from replenishment round 17; panel c is contributions and commitments from replenishment round 18.We were unable to obtain project-level data through correspondence for years after 2021. Thus, we turned to the project-level data reported by the World Bank to IATI. We collect a complete set of disbursements for all projects reported by World Bank to IATI and then filter down to disbursements that are labeled by the World Bank as belonging to the sectors “Health”, “Public Administration – Health”, or “Health Facilities and Construction”. We then apply the reported sector percentage to the disbursement values to obtain a disbursements for health estimate that could be fed into the rest of our pipeline. Details on how we estimated the cost of providing technical assistance and program support for these institutions are highlighted below in the section titled “Calculating the technical assistance and program support component of development assistance for health from loan- and grant-making channels of assistance.”

S3.3.1 Regional development banks

The Asian Development Bank (ADB) and Inter-American Development Bank (IDB) maintain their own loan databases, which we used to estimate disbursements.[15,16,81] To estimate health disbursements from the African Development Bank (AfDB), data were received via correspondence with loan accounting assistant A. Fofana[35] To estimate health disbursements from the Asian Development Bank (ADB), data were collected from IATI and through correspondence.[111] Table S3.7 provides a summary of the data sources used across the regional banks.

For AfDB, we received project-level disbursement data from 2001 through 2024. For pre-2001 estimates, data from the Compendium of Statistics were used. Donor information for projects funded by the African Development Fund was based on subscription information from the ninth to 14th replenishment reports.[82] The sole donor to projects implemented by the Nigeria Trust Fund was Nigeria, and all income for African Development Bank projects was attributable to other sources. From AfDB’s financial reports, we also extracted the proportion of the bank’s income that comes from loans and other derivatives and used that to estimate the share of DAH sourced from the bank’s own resources.[15]

For ADB, we received project-level disbursement data from 1990 through 2021, including recipient, project title and description, and annual disbursement. For years after 2021, we use IATI data, which is filtered down to health-related projects using the reported OECD sector codes.[111] A keyword search was performed to identify health focus areas of the projects. For income source of ADB, data for country donations were extracted from the replenishment reports of Asian Development Fund, and we manually divided the four or five years’ replenishment into annual contributions.[83] We extracted the total revenue of Ordinary Capital Resources and Asian Development Fund from the financial reports of ADB to generate the total envelope of income sources and subtracted the country contribution to provide the estimate for resources coming from the bank itself. Using the fraction for the Bank’s own resources and country contribution, we provided the estimate of income source for each project. We also extracted the proportion of the Asian Development Bank’s OCR revenue that comes from loans from financial reports, and used that to estimate the share of DAH sourced from the bank’s own resources.[125]

For the Inter-American Development Bank, we receive correspondent data containing health disbursements for years 2015-2024. However, we were unable to obtain correspondent data for years 2022 and 2023 specifically, and thus used IATI to impute these years. For years prior to 2015, we use the IDB’s online project database, which provided commitments on all projects, and these were used to estimate disbursements. .The contribution quotas by donor for the Fund for Special Operations from 1990–2016 were extracted from annual financial reports for the IDB. The ratio of each donor’s contribution to total contribution was applied to total disbursements to assign income sources. In 2017, the Fund for Special Operations was transferred to the ordinary capital. Donor assignments from 2017 onward are unallocable. From financial reports, we also extracted the proportion of the IDB’s OCR income that comes from interest on developmental assets, and used that to estimate the share of DAH sourced from the bank’s own resources.[16]

Table S3.7 Summary of data sources for the regional development banks

This figure indicates the data available and used to estimate DAH. (X) indicates that project-level data are present in the dataset. (-) indicates that project-level data are not present in the dataset.

Institution	*Data source*	*Commitments*	*Cumulative disbursements*	*Yearly disbursement*	*Notes*
African Development Bank (AfDB)	Compendium of Statistics	X	-	(Aggregate – not at the project level)	The Compendium of Statistics was not available for 1990–1993, 1995, 1998–1999, or after 2002; we estimated yearly disbursements using the average of neighboring disbursements
	Correspondence	-	-	X	Annual loan disbursements from 2001 through December 2024
Asian Development Bank (ADB)	IATI	X	X	X	The IATI data was used to estimate disbursements for the period after 2021, for which we could not obtain correspondent data.
	Correspondence	X	X	X	Annual grant loan disbursements from 1990 through2024.
InterAmerican Development Bank (IDB)	Online projects database	-	X	-	As yearly disbursement amounts are not provided in the online database, we estimated yearly disbursements by allocating cumulative disbursements over each year of the project. We did this to estimate disbursements for years 1990-2015.
	Correspondence	-	-	X	Loan disbursements from 2015 – 2024, but missing 2022 and 2023.
	IATI	X	X	X	We used the data reported by IDB to IATI to impute disbursements in 2022 and 2023.

Figure S3.7 Disbursements by the African Development Bank

This plot shows final estimated disbursements disaggregated by source of funding.

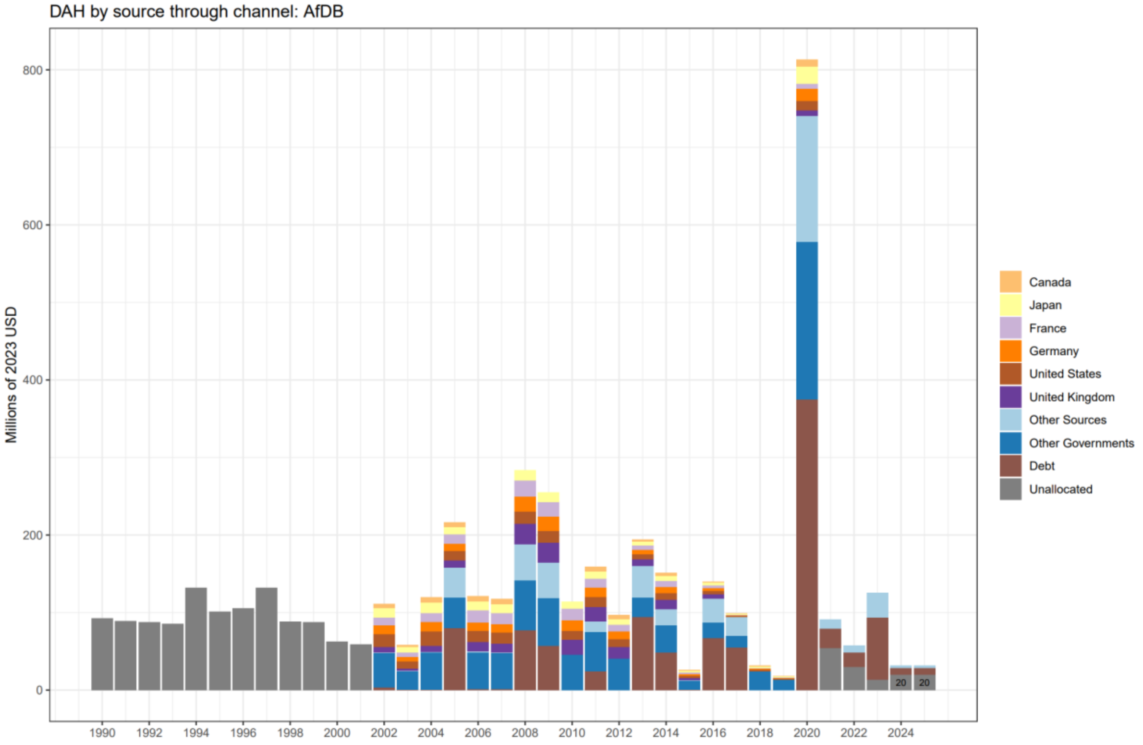

Figure S3.8 Disbursements by Asian Development Bank

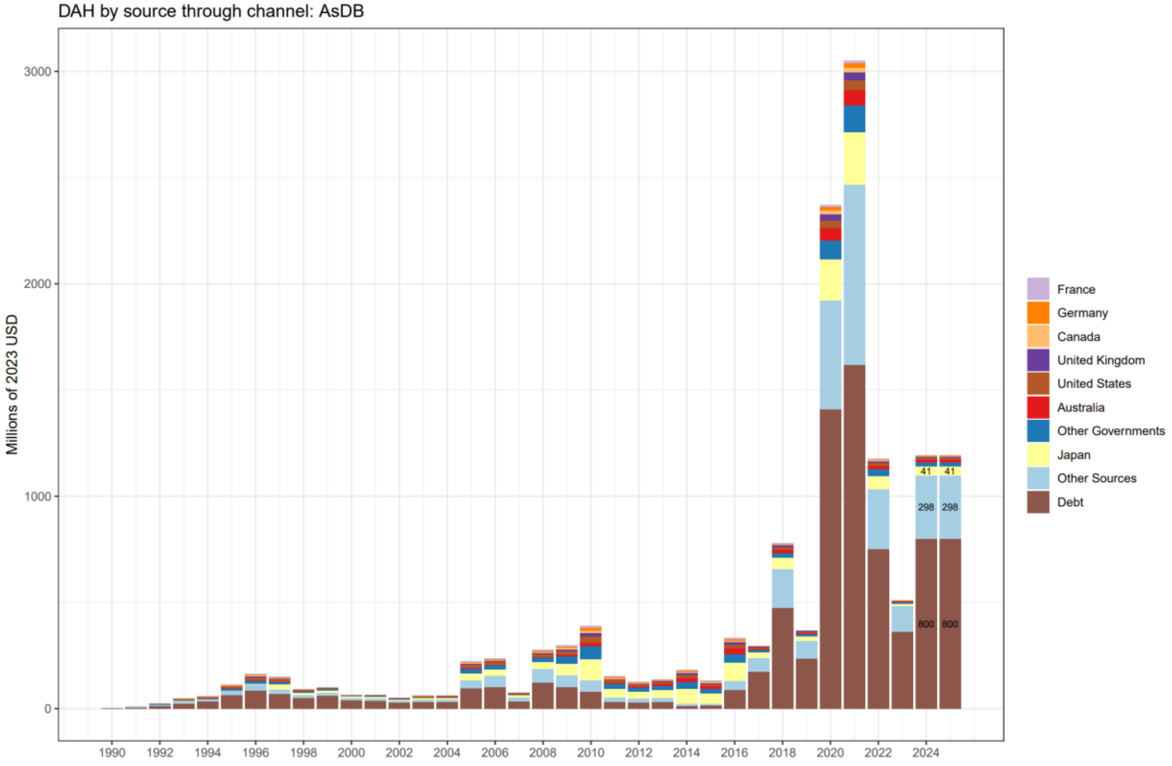

Figure S3.9 Disbursements by Inter-American Development Bank

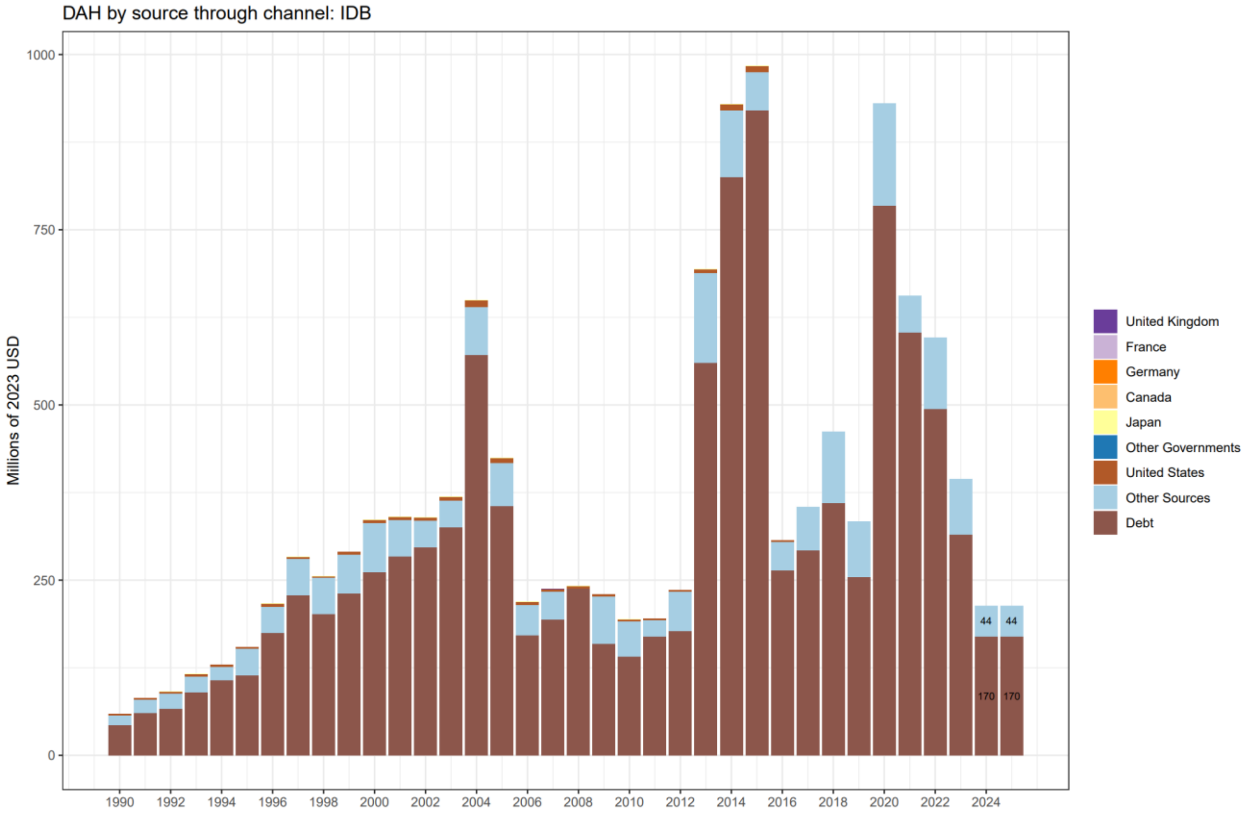

S3.4 Tracking contributions from the Global Fund and Gavi

The Global Fund

The grants database made available online by the Global Fund provides grant-level commitments and annual disbursements from its inception in 2002 to the present year.[22] In addition, sources of funding were compiled from the Global Fund contributions dataset and annual reports, all downloaded from the Global Fund website.[23,24] Regional grants were split evenly between all countries identified in the regional grant documents found on the Global Fund website. Figure S3.10 shows the Global Fund's annual disbursements envelope calculated from its project database from 2002 through 2024 as well as Covid-19 Response Mechanism data. Figure S3.11 shows the Global Fund's annual disbursements and commitments from its project database, not including the Covid-19 Response Mechanism amount.

Figure S3.10 The Global Fund's disbursements including Covid-19 Response Mechanism

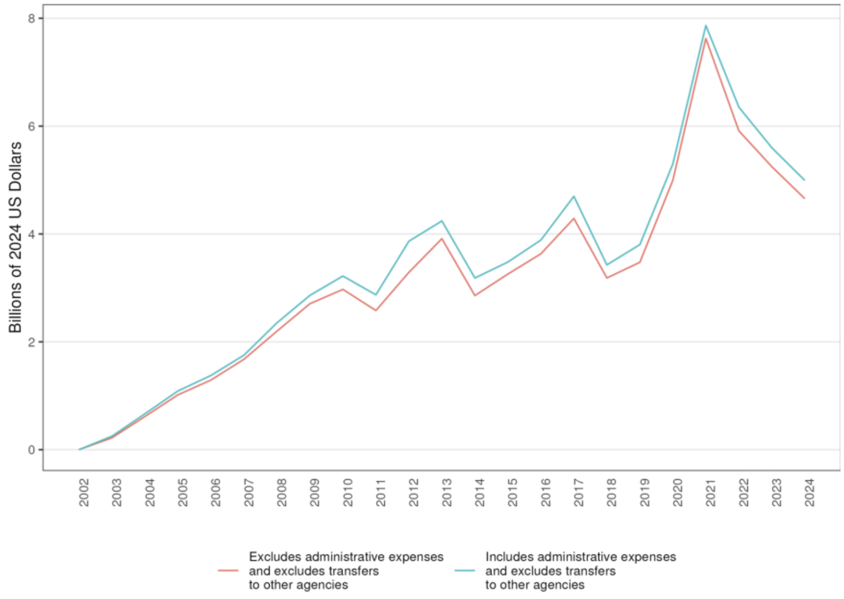

Source: The Global Fund pledges and contributions 2024

Figure S3.11 The Global Fund's commitments and disbursements
The solid line shows commitments from the Global Fund's online grants database. The dashed line shows disbursements estimated from the online grants database.

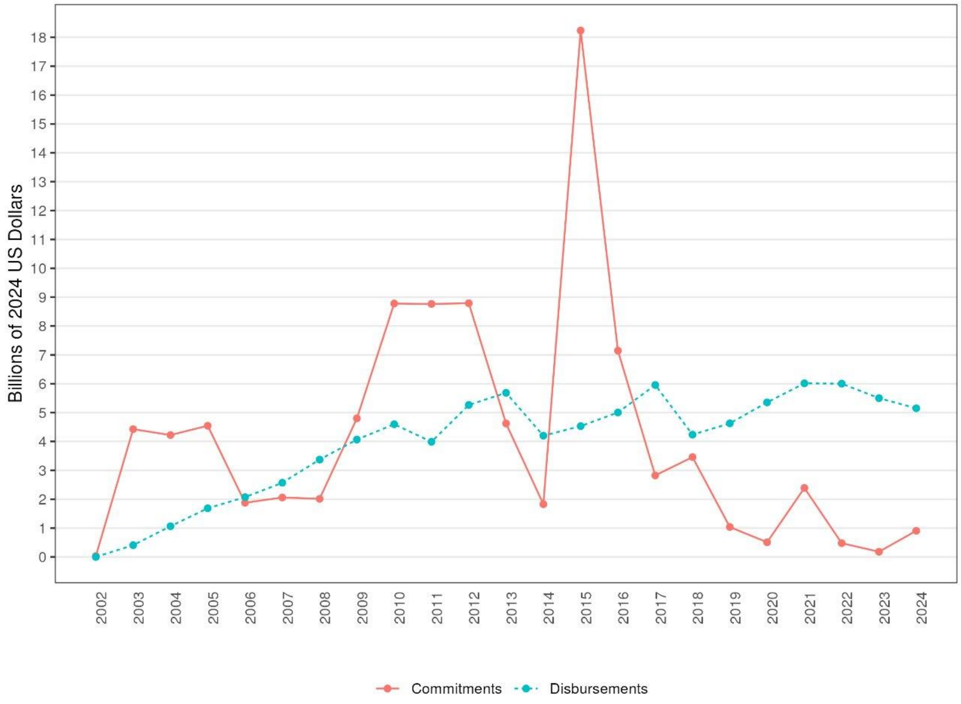

Source: IHME DAH Database (2024)

S3.4.1 Gavi, the Vaccine Alliance

Gavi provided publicly available project-level data on commitments, disbursements, and investment cases from 2000 through 2021.[18,21] Gavi's annual DAH was defined as the sum of (1) project-level disbursements by year paid; (2) investment cases (one-time investments in disease prevention and control); and (3) administrative and work plan costs. Data from Gavi's online databases include expenditure for (1) and (2), but not (3). However, project-level data from the CRS for 2007–2012 did include administrative and work plan costs, so disbursements data from the online database were adjusted to match the CRS in those years. The average fraction of administrative and work plan costs was added to total disbursements in 2000–2006 and 2013–2015, the years in which the CRS did not include these data. Contributions data from Gavi's website as well as annual reports from the International Finance Facility for Immunisation (IFFIm) and Advance Market Commitment for Pneumococcal Vaccines were used to determine Gavi's annual income.[19,20,84]

All of the data sources used for Gavi estimates were complete through 2021. For years past 2021, disbursement data is no longer available from Gavi so we have switched to using IATI data.[111] Gavi disbursements are assigned to health focus areas including child and newborn vaccines, HSS, and non-communicable diseases as documented in Table S3.7 above. Of note, we reclassified all Gavi health system strengthening projects as maternal, newborn, and child health–specific health system strengthening disbursements.

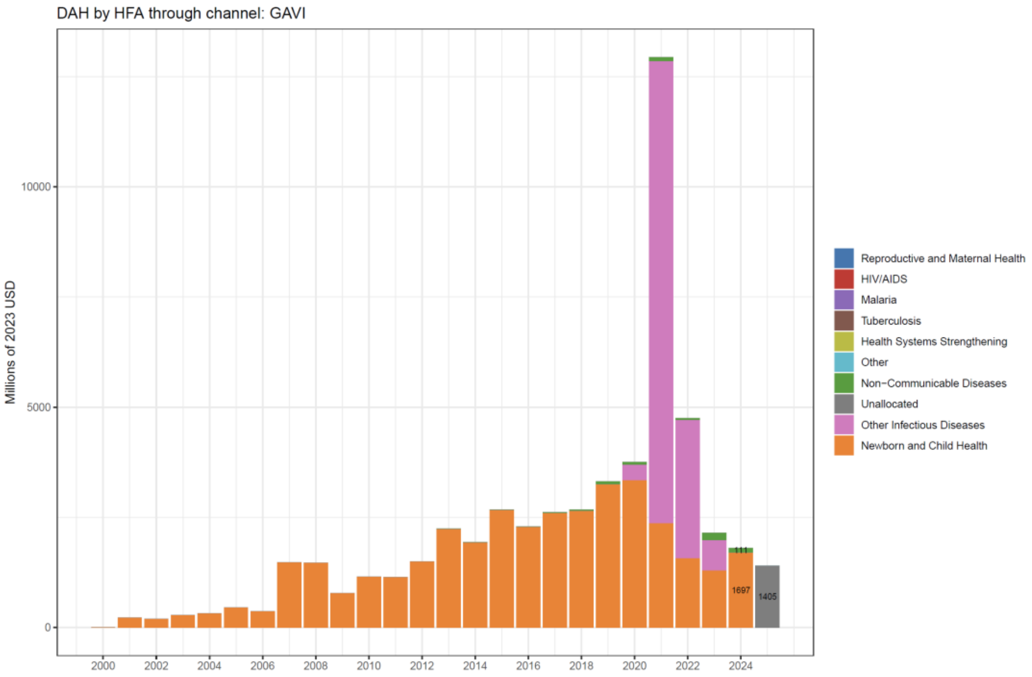

Source: IHME DAH Database (2024)

Figure S3.12 Gavi's disbursements

The graph shows the disbursements from Gavi's online database and IATI reporting, which is adjusted using Gavi expenditure data reported to the Creditor Reporting System (CRS) to add administrative and work plan costs to the total. The pink spikecomes from Gavi COVAX data, explained below in S3.11.

S3.4.2 Coalition for Epidemic Preparedness and Innovation

CEPI provided a complete dataset of all disbursed funding by disease from 2017–2024 and all income received from donors from 2018–2024. Funding data were provided per target vaccine and were classified between the other infectious diseases health focus area and the health systems strengthening other infectious diseases health focus area.

S3.5 Tracking expenditure by United Nations agencies active in the health domain

Data on income and expenditures were collected for six UN agencies: WHO, UNICEF, UNFPA, UNAIDS, Unitaid, and PAHO. The data sources and calculations for each are described in detail below. Similar to the bilateral channels, we extracted budget data for the UN agencies to predict DAH for years for which we did not have health expenditure data. Model choices and budget measures for UN agencies are presented in Table S3.3.

S3.5.1 World Health Organization

Data on WHO's budgetary and extrabudgetary income and expenditure were compiled from annual reports and audited financial statements released by WHO.[85] Income data were extracted from WHO's assessed and voluntary contributions, while expenditure data were extracted from both budgetary and extrabudgetary spending reports. For financial statements representing activities over a two-year period, both income and expenditure data were divided by two, to approximate yearly amounts, and dollars were deflated using the US GDP deflator specific to the reporting year. Expenditures from trust funds, regional offices tracked separately, and associated entities not part of WHO's program of activities, such as UNAIDS and the Global Fund trust funds were excluded. Expenditures from supply services funds were also excluded, as these expenditures pertain to services provided by WHO but paid for by recipient countries. Additionally, WHO projects tracked as paid Ebola expenditure were extracted from the UNOCHA Financial Tracking System database and included as WHO health expenditure.

Disbursement data were not available for WHO in 2024. Much like the bilateral agencies, the ratio of DAH to the total program budget was estimated for 1990–2023 and then predicted for 2024 using a three-year weighted average of 2021–2023. The predicted ratio was then multiplied by the observed program budget for 2024 to get the estimates of DAH.

Assessed contributions have been adjusted to 76% of reported values to follow the research conducted and published by OECD – Review of the ODA coefficients of select health-related organisations on Annex 2 and the Single Table of the Converged Statistical Reporting Directives for the Creditor Reporting System (CRS) and the annual DAC Questionnaire.[100] The OECD estimate is negotiated with WHO as the HQ "has a partly normative function and provides support to all member countries, some of which are not ODA-eligible."[100]

To disaggregate WHO's DAH into health focus areas and program areas, we use WHO's programme budget. Starting in 2020, the WHO no longer breaks out their budget into the specific disease areas that we leveraged for the purposes of this disaggregation. Thus, we use IATI data to estimate health focus areas and program areas for years 2020 and later.[111] We extracted the disbursements that WHO has reported to IATI and ran our keyword search over the project titles and descriptions provided in the data. We used the results of the keyword search, in combination with the OECD sector codes and percentages reported by WHO, to obtain health focus area fractions which are similar to those obtained by the programme budget in earlier years. These fractions are applied to WHO's DAH envelope.

S3.5.2 United Nations Population Fund

Data on income and expenditure were extracted for UNFPA from its audited financial statements.[72] As the 1990–2005 statements represent activities over a two-year period, income and expenditure data were divided by two in order to approximate yearly amounts.

Income and expenditures associated with procurement and cost-sharing activities were excluded from estimates of health assistance because UNFPA uses cost-sharing accounts when a donor contributes to UNFPA for a project to be conducted in the donor's own country. Since this money can be considered domestic spending that goes through UNFPA before being returned to the country in the form of a UNFPA program, it is not included in calculations of total DAH. UNFPA's additional expenditures for these projects come from trust funds, special funds, or regular resources and are therefore captured in our estimates. To estimate disbursements by health focus areas, UNFPA's total health expenditure was multiplied by the proportion of funding reported for each program area from annual reports from 1997 through 2013, from the UNFPA transparency portal for 2014 through 2018, and from the 2019–2023 Statistical and Financial Reviews for years 2019–2023. Maternal and child health spending classified as "other" was split equally between the maternal and child health program areas. Health spending in 2018 that was classified as going toward "integrated sexual and reproductive health services" was split equally between maternal health, family planning, other reproductive health, and other HIV/AIDS program areas. Additionally, UNFPA projects tracked as paid Ebola expenditure were extracted from the UNOCHA Financial Tracking System database and added to UNFPA health expenditure.

The disbursement data for UNFPA were available through 2023. For year 2024, the ratio of DAH and income was estimated for 1990–2023 and then predicted for 2024 using a three-year weighted average of 2021 – 2023. The predicted ratio was then multiplied by observed 2024 income to get the estimates of DAH.

S3.5.3 United Nations Children's Fund

Data on income and expenditure for UNICEF were extracted from its audited financial statements.[69] As these statements represent activities over a two-year period from 1990 to 2011, income and expenditure data were divided by two to approximate yearly amounts. The audited financial statements from 2012 onward are produced on an annual basis.

Since UNICEF's activities are not limited to the health sector, the fraction of UNICEF's expenditure that was for health was estimated using either financial data from correspondence (2001–2013 observed data were used to estimate 1990 through 2000 expenditure) or a combination of annual reports and annual results reports from 2014 through 2023. The annual results reports provide the proportion of funding for each program area, and the average of 2014–2016 proportions was used to estimate the spending proportion for the years 1990–2013. In the annual results report, HIV/AIDS funding and nutrition funding (as of 2014) was reported separately from health funding, so the percentages spent on each health program were proportioned based on total spending for health. Furthermore, UNICEF projects tracked as paid Ebola expenditure were extracted from the UNOCHA Financial Tracking System and added to estimates for UNICEF's health expenditure.

The product of observed program budget and the three-year weighted average of the DAH to budget was used to predict DAH in 2024.

S3.5.4 Joint United Nations Programme on HIV/AIDS

UNAIDS income and expenditure data for both its core and noncore budgets were extracted from its audited financial statements.[67] As financial data are provided on a biennial basis in all years prior to 2012, those values were divided by two to obtain yearly amounts. Dollars were deflated using the US GDP deflator specific to the reporting year.

For UNAIDS, budget measures were available only for a subset of reported total disbursements. UNAIDS reported total expenditure, which combined Unified Budget and Workplan (UBW) and non-UWB components, but only UBW budget data were available.[68] To predict DAH for UNAIDS in 2024, disbursements for that year were estimated by multiplying the observed UBW budget by the three-year weighted average of the ratio of DAH to the

UWB budget. UNAIDS disbursements were assigned to HIV/AIDS and TB program areas as documented in Table S3.7 above.

S3.5.5 Unitaid

Data on project-level disbursement were obtained through correspondence with Unitaid. Income data were extracted from the annual financial statements downloaded from Unitaid's website.[9] The project-level data provided covered project disbursements from 2007 through 2024, and project budget commitments were provided for 2024.

S3.5.6 Pan American Health Organization

The Pan American Regional Office for WHO, or PAHO, reports its income and expenditure in its biennial financial report.[10,86] The funds transferred through the "Rotating Fund" were excluded because developing countries fund this procurement of health commodities which are then used within that funding country, and it therefore does not fit the definition of DAH.

As the financial data are provided on a biennial basis (with the exception of 2010–2016, where single-year financial reports were available), the quantities were divided by two to obtain yearly amounts. Dollars were deflated using the US GDP deflator specific to the reporting year.

Correspondence with PAHO revealed that data from the financial statements include both program and non-program funds. The latter include funds that countries provide PAHO, so that PAHO can reinvest these funds into the countries' national health systems. These funds should not be included as development assistance for health, and PAHO provided corrected disbursement numbers for 2008–2013. The corresponding disbursement numbers for 2014 and 2015 were identified in the PAHO End-of-Biennium Assessment 2014–2015. These funds were provided as biennial disbursements, so they were divided by two to obtain yearly disbursements. The ratio of program disbursements numbers provided by PAHO and the sum of program and non-program funds collected from financial statements was taken for the years 2008 to 2015. The average ratio was calculated, and this ratio was multiplied through disbursement numbers collected from financial statements from earlier years. In this way, program and non-program funds collected from audited statements from earlier years were adjusted to estimate DAH.

For PAHO, disbursement data were not available for 2024. PAHO provided budget information along with disbursements for 2008 to 2023. The average ratio between spending and budget was calculated over the years 2008 to 2023, and this ratio was used to estimate 2024 disbursements.

S3.6 Tracking development assistance for health from private foundations

Previous studies on foundations outside the US have documented the severe paucity of reliable time series data and lack of comparability across countries.[87] Hence, this research focused efforts on tracking only US foundations.

S3.6.1 US foundations

Candid (previously The Foundation Center), maintains a database of all grants of $10,000 or more awarded by over 1,000 US foundations. The Foundation Center coded each grant by sector and international focus and therefore is able to identify global health grants. IHME purchased a customized dataset with cross-border health grants and health grants to US-based international programs from 1992 to 2019 from the Foundation Center.[32] Grants from the Gates Foundation, which were tracked separately, were excluded. Additionally, grants to channels that this research already tracks were excluded. Due to impracticality of acquiring the Candid dataset, as well as in order to obtain a more holistic sample of the data, we have moved to obtaining 990 data from GivingTuesday's 990 data lake. In order to filter the grants obtained from the data lake to only include DAH, we only included grants to LMIC countries as well as performing a health keyword search.[-110]

The Foundation Center adopted a new classification methodology as of FGH 2016. The Foundation Center provided historical data based on the new classification system from 2002 to 2012. To obtain the series from 1992 to 2001, we multiplied a weighted fraction calculated based on both old and new classification data values from 2002 through 2004 by the old data series (1992 to 2001) we had previously obtained.

$$(Weighted\ fraction) = \left(\frac{1}{2}\right)\left(DAH_{new\ classification}\right)/\left(DAH_{old\ classification}\right)2002 + \left(\frac{1}{3}\right)\left(DAH_{new\ classification}\right)/\left(DAH_{old\ classification}\right)2003 + \left(\frac{1}{6}\right)\left(DAH_{new\ classification}\right)/\left(DAH_{old\ classification}\right)2004$$

$$(DAH\ Estimate_t) = (Weighted\ fraction)(DAH\ Observed_t)$$

where DAH Observed is the old data values for the series 1992 through 2001

Additionally, as our new filtering method is more restrictive than the historical one using the Foundation Center data, we scaled down our historical time series using the ratio between the new data and last year's 2020-2021 data which used a scraping and estimation method that more closely resembled the Foundation Center numbers. Finally, due to missing 2022 data year 990 reporting, we also estimated missing foundation values using 2020-2021 data for each missing foundation.

To estimate total health grants in 1990–1991 and 2023–2024, natural log of US foundation DAH was regressed on the lagged natural log of US GDP per capita and year using ordinary least squares estimation. The missing years of data were predicted based on estimated regression coefficients from the equation. Exponents of the predicted values were used as final estimates

$$(ln\ Foundation_t) = \alpha + 1.\beta_1(\ln US\ GDP\ per\ capita_t) + \beta_2(year_t) + \varepsilon$$

Details on how we estimated the cost of providing technical assistance and program support for these US foundations are highlighted below in the section titled "Calculating the technical assistance and program support component of development assistance for health from loan- and grant-making channels of assistance."

S3.6.2 Gates Foundation

The Gates Foundation has been the single largest grant-making institution in the health domain since 2000; hence, additional research was undertaken to accurately capture its annual disbursements. The Gates Foundation's IRS 990PF filings for years 1999–2008, which report all global health grants disbursed per year, were downloaded from the Gates Foundation's website. Additionally, disbursement data for years 2009–2023 were collected from the Gates Foundation's online grants database, the OECD CRS, and personal correspondence. The OECD CRS data were used to identify NGOs that are double-counted from other data sources we use.

To estimate Gates Foundation DAH for 2024, we rely on the Gates Foundation's announced total budget for the year 2024, announced in their 2024 Annual Letter[113]. Using the 2023 total budget, which was announced in the 2023 Annual Letter, we calculate the fraction of the 2023 budget that went to DAH, based on our 2023 DAH estimate[114]. Then we apply this fraction to the 2024 total budget to obtain a total DAH envelope for 2024. Note that, since Gates has also announced their 2025 budget in a recent press release, we also use this method to obtain a 2025 estimate for the Gates foundation[115].

S3.6.3 European Economic Area

DAH estimates for the European Economic Area were based on two downloadable project datasets available online as well as correspondence data. IHME DAH data for 2014 to 2018 were obtained from projects in the 2009–2014 grant period dataset with program area "public health initiatives," and 2007–2012 data were obtained from projects in the 2004–2009 grant period dataset with sector "health and childcare." Childcare projects not related to health were identified and excluded based on manual screening and keyword search for the following terms: "NURSERY" "ORPHANAGE" "RECREATION" "FOSTER" " CARE HOME " " CHILDREN S HOME " " CHILD DEVELOPMENT CENTRE " " YOUTH CENTER" " YOUTH CAMPS " " RESIDENTIAL CENTRE" " RESIDENTIAL CARE FACILITIES " "CHILDCARE" " INFORMAL EDUCATION FOR CHILDREN " " PREVENT INSTITUTIONALISATION " " SPORTS FIELD" " SPORT FIELD" " SPORTS FACILIT" " SPORTS ACTIVIT" " SPORTS INFRASTRUCTURE" " REINTEGRATION OF JUVENILE OFFENDERS ".

We determined that no health projects to low- and middle-income countries were implemented by the EEA prior to the 2004–2009 grant period, based on the report on the Financial Instrument 1999–2003 and the Financial Mechanism 1994–1998.[88,89] Project summary variables were obtained through correspondence and were used together with project titles to assign DAH to health focus areas using a keyword search. To assign annual funding for projects that were implemented across multiple years, project grants were split based on the average daily spending across the duration of the project and the proportion of the year that the project was in implementation. The relative contributions of donors to the EEA grants (Norway, Iceland, and Liechtenstein) were obtained as constant ratios based on the 2016–2017 Annual Report.[90]

An open call for projects in the EEA's third grant period occurred in 2014, the first projects of which began disbursing in 2019 and are set to conclude in 2021. Data for the 2019–2023 grants came from personal correspondence.

S3.7 Tracking non-governmental organizations

Currently, there are no centralized, easily accessible databases for tracking program expenses of the thousands of NGOs based in high-income countries that are active in providing development assistance and humanitarian relief worldwide. This study relies on several data sources for NGOs. Data from 1990 to 2014 largely rely on CRS data as well the United States Agency for International Development's Report of Voluntary Agencies (USAID's VolAg report).[27] The report, which includes both US-based and international NGOs that received funding from the US government, provides data from 1990 to 2014 not only on the US government portion of their funding but also on domestic and overseas expenditures for these NGOs as well as their revenue from US and other public sources, private contributions, and in-kind. Total revenue and expenditure data obtained from the NGOs' IRS tax forms, accessed through the GuideStar online database, were also used in tracking NGOs incorporated in the US particularly from 2015-2018.[26] This included domestic, overseas, and total expenses, and revenues from all public and private sources, and this dataset is not limited to NGOs with US government funding.

First, to track disbursements from OECD donor countries to NGOs, we utilized channel codes present in the CRS database. The code 21000 identified international NGOs, and the code 22000 identified donor-country-based NGOs. To remove double-counting, we conducted a keyword search on channels where the donor country was the United States to exclude NGOs present in the USAID VolAg report and GuideStar dataset. Allocation of funding to health focus areas for NGOs tracked through the CRS was assigned as described in the section "Disaggregating by health focus area," based on a keyword search of five descriptive variables in the CRS: project title, short description, long description, channel name, and channel reported name. For NGOs tracked in the USAID VolAg report and GuideStar dataset, allocation of funding to health focus areas was assigned as described in the section "Disaggregating by health focus area," based on a keyword search of the NGO's description given in the VolAg

report. Due to CRS projects containing more complete descriptions than the VolAg report descriptions, the health focus area allocations from CRS were prioritized over those from the VolAg report for US NGO agencies that appeared in both sources.

To use the USAID VolAg data GuideStar dataset, several challenges were overcome. We outline these challenges here and describe below the methods employed to estimate a consistent series of DAH channeled through NGOs despite these challenges. First, with the exception of the Gates Foundation, it was impossible to track the amount of funding from US foundations routed through US NGOs, which may have led to double-counting in estimates of total health assistance. The second challenge relates to the incompleteness of the universe of NGOs captured through the VolAg report and GuideStar Research Fundamentals dataset. The VolAg report provides data on NGOs that received funding from the US government. While this covers many of the largest NGOs, it is not a comprehensive list. A related problem is that the VolAg report and GuideStar dataset only include NGOs that received funds in a given year. While many of the largest NGOs are consistently funded by the US government and are therefore in the dataset every year, not all NGOs are reported across all years. Third, health sector–specific expenditure is not reported in the VolAg or systematically reported in IRS tax forms. The VolAg does report overseas expenditure but does not disaggregate this expenditure by sector. Fourth, complete data are lacking in several time periods. The 2016 VolAg provided data through 2014. Furthermore, prior to 1998 the VolAg report did not include international NGOs. Attempts were made to compile other data on the health expenditures of the top international NGOs, in terms of overseas expenditure, by searching other websites for financial documents and contacting these organizations directly. Getting reliable time series data before 2000 proved to be extremely difficult for even this small sample of international NGOs. For estimates past 2018, due to difficulties in acquiring the GuideStar dataset and the complexity of acquiring new data methods, we did not acquire new data for the organizations in the GuideStar dataset and instead held that portion of our 2018 estimate stable. We still updated years from 2019 onwards using the data from the CRS. Table S3.8 summarizes the number of NGOs included each year in the USAID report, the number of NGOs in the sample by year, and the number of NGOs for which health expenditure data were successfully compiled.

Table S3.8 Summary of health expenditure sampling for US non-governmental organizations in the study

Year	*Number of US NGOs in VolAG report*	*Number of international NGOs in VolAG report*	*Number of US NGOs in IHME sample*	*Number of US NGOs from sample for which data on health expenditure were found*
1990	267	-	16	9
1991	334	-	19	14
1992	385	-	18	15
1993	411	-	17	12
1994	424	-	17	10
1995	416	-	16	12
1996	423	-	21	14
1997	425	-	23	18
1998	435	42	24	22
1999	438	-	33	28
2000	433	50	34	28
2001	442	51	33	26
2002	486	58	33	27
2003	507	54	42	32
2004	508	55	47	33
2005	494	59	45	36

2006	536	67	50	38
2007	556	68	50	40
2008	565	78	58	48
2009	580	90	57	45
2010	579	94	69	57
2011	595	112	73	63
2012	579	94	69	60
2013	519	113	69	52
2014	485	106	73	54
2015	-	-	67	53
2016	-	-	115	98
2017	-	-	81	69
2018	-	-	109	93

A random effects regression model was fit to predict health expenditure as a fraction of total expenditure using the data for the sampled NGOs. A random effects model was chosen because the sample included observations for several NGOs for multiple years. A random effects model allows for the effect of each type of NGO to be captured distinctly. This model was used to predict the fraction of expenditure spent on health for the remaining NGOs. To ensure that the predicted health fractions were bounded between zero and one, the regression utilized the logit-transformed health fraction as the dependent variable. Since several NGOs in the sample were observed for multiple years, the regression included a random effect that varied by NGO. Five of the nine variables used to predict the health fraction were drawn from the VolAg reports and the GuideStar dataset. They were (1) fraction of revenue from in-kind donations, (2) fraction of revenue from the US government, (3) fraction of revenue from private financial contributions, (4) overseas expenditure as a fraction of total expenditure, and (5) calendar year. The remaining four variables used to predict the health fraction were binary indicators that were constructed based on keyword searches on the NGO name and NGO description. For both the NGO name and description, a keyword search was conducted to indicate whether the name or description was sufficiently health-related. Another keyword search was conducted independently on the NGO names and descriptions for keywords that indicated if the NGOs might focus on something other than health. These four indicators proved excellent predictors of health fractions.

$$\begin{aligned} logit(\ NGO - specific\ DAH_{it}) &= \alpha +\ \beta_1(Inkind\ contributions\ fraction_{it}) \\ &+\ \beta_2(US\ government\ contributions\ fraction\ _{it}) \\ &+ \beta_3(Private\ financial\ contributions\ fractions_{it}) \\ &+ \beta_4(Overseas\ expenditure\ as\ a\ fraction\ of\ total\ expenditure_{it}) \\ &+ \beta_5(Health - related\ name_{it}) + \beta_6(Non - health - related\ name_{it}) \\ &+ \beta_7(Health - related\ description_{it}) + \beta_8(Non - health - related\ desciption_{it}) + U_i +\ \varepsilon \end{aligned}$$

Overseas health expenditure was calculated for individual NGOs in each year by multiplying the estimated health fraction and total overseas expenditure. For the NGOs that were sampled, the observed health fraction acquired through data collection was used. For the unsampled NGOs, the fitted fraction from the previously described random effects regression was used. Total overseas expenditure, reported in the VolAg, was not available for 2015–2018. For US-based NGOs, the 2018 NGO overseas fraction was calculated by regressing the logit transformed observed overseas fraction on a linear time trend using ordinary least squares, for each NGO independently. For these cases, the overseas health fraction was calculated as the product of estimated overseas fraction, estimated health fraction, and total expenditure found in the IRS 990 forms.

$$logit(Observed\ overseas\ health\ expenditure_i) = \alpha +\ \beta_i(year_t) + U_i +\ \varepsilon$$

At this point, two reasons remained why the overseas health expenditure for some NGOs remained unknown. First, if an observation was non-US-based, then IRS tax forms were not available and total overseas expenditure could not be calculated. Second, if an NGO was reported in the VolAg in multiple years but not for an intermittent year, no NGO-specific data were available for the gap year. This would be the case if an NGO received support from the US government one year and then again in a nonconsecutive year. For all three of these scenarios, a panel-based hierarchical linear regression model was used to fill in the overseas health expenditure gaps. Total overseas health expenditure (measured at the NGO-year level) was regressed on US GDP per capita and US bilateral DAH disbursed. Because the US government funds many of these NGOs, US bilateral DAH was an excellent predictor of NGO DAH. A flexible model was employed to allow both the GDP and US government DAH coefficients to vary randomly across NGOs, such that each NGO employed a unique (but not independent) relationship between overseas health expenditure, GDP, and US government DAH. A random intercept was also included to capture the significant unobserved heterogeneity present in our set of NGOs. Once fit, this model was used to predict overseas health expenditure for all remaining gaps.

$$(NGO\ DAH_{it}) = \alpha + \beta_{1i}(US\ GDP\ per\ capita_t) + \beta_{2i}(US\ bilateral\ DAH\ per\ capita_t) + U_i + \varepsilon$$

Expenditures financed from each revenue source were then calculated by multiplying overseas health expenditure by NGO-specific revenue fractions. Expenditures from in-kind sources were deflated by a constant fraction. This was determined by comparing the federal upper limit and average wholesale price valuations of drugs on the WHO's Model List of Essential Medicines from the RED BOOK Expanded Database.[28,29] Figure S3.13 shows the income of the NGOs in the universe of US- and non-US-based NGOs that were tracked in this study from 1990 to 2020 in constant 2023 US dollars.

Figure S3.13 Total revenue received by non-governmental organizations

This graph shows total revenue for all sources, both public and private, received by NGOs. The pink bar represents the private sources while the other government sources are also broken out.

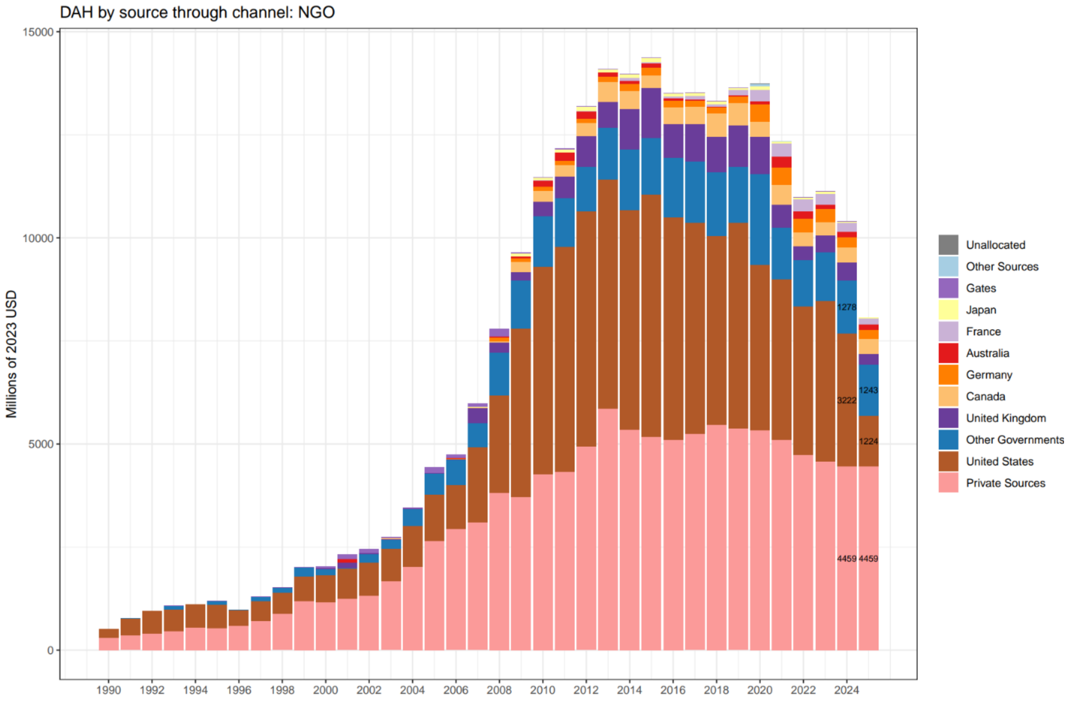

Source: IHME DAH Database (2024)

S3.8 Tracking development assistance for health from China

The estimates of DAH contributions from China are based on data from diverse sources including government reports and international development agencies databases. We generate estimates for bilateral and multilateral contributions from China separately and aggregate to obtain the total DAH contributions from China. We use project descriptions and reports where available to determine health focus areas of the contributions. For years with limited data, we use various methods inclusive of interpolation and modeling to generate estimates.

Following the addition of the China International Development Cooperation Agency (CIDCA) into our estimates last year, we now include five Chinese agencies in our bilateral DAH envelope: the National Health Commission, the Ministry of Commerce, the Ministry of Education, the Export-Import Bank of China, and CIDCA.

Detailed description of the methods for generating the estimates are reported in Micah AE, Zhao Y, Chen CS, et al.[97]

We also leverage AidData's Global Chinese Development Finance Dataset to obtain project-level information, which we use to estimate health-focus-area fractions for MOFCOM and the Export-Import Bank of China.[112] We filter the database down to health sector projects and run our keyword search on the project titles and descriptions and then aggregate the results to obtain health focus area fractions for each agency and year.

S3.9 Calculating the technical assistance and program support component of development assistance for health from loan- and grant-making channels of assistance

The following methods were used to estimate the costs incurred by loan- and grant-making institutions for administering and supporting health sector loans and grants, which includes costs related to staffing and program management.

Data on the total administrative costs were compiled for institutions in our universe for which these data were readily available: IDA, IBRD, ADB, AfDB, IDB, the Gates Foundation, US Foundations, CEPI, the Global Fund, Gavi, UNICEF, UNFPA, WHO, Unitaid, PAHO, UNAIDS, USAID, the UK Department for International Development (DFID), Japan International Cooperation Agency (JICA), Norwegian Aid Agency (NORAD), and the Swedish International Development Agency (SIDA). The sources of data for the institutions in this sample are summarized in Table S3.9. The ratio of total administrative costs to total grants and loans was calculated for each source by year. It was assumed that the percentage of operating and administrative costs devoted to health would be equal to the percentage of grants and loans that were for health. In other words, if 20% of a foundation's grants were for health, the model assumed that 20% of administrative costs of the foundation were spent on facilitating these health grants. Given this assumption, the ratios of the observed administrative costs to grants/loans were used to estimate the in-kind contribution made by each of these organizations toward maintaining their health grants and loans. For the institutions not in this sample, the ratio from the institution most similar to it was used to arrive at an estimate of in-kind contributions. For example, for US foundations we selected the top 10 and bottom 10 US foundations based on total disbursements across the entire time period. For each foundation, we calculated the ratio of the "cash basis" column of total operating and administrative expenses to grants paid. We then used the average of these top 10 and bottom 10 foundations ratio as the in-kind ratio across time. Total in-kind contributions from all grant- and loan-making global health institutions are shown in Figure S3.14.

Table S3.9 Summary of data sources for calculating in-kind contributions

Organization	*Source*	*Notes*
Gates Foundation	990 tax returns (1999–2006)<br>BMGF Trust financial statements (2007–2023)	Used "cash basis" column to calculate ratio of total operating and administrative expenses to grants paid.<br>Used "grants expenditure"

		statement to calculate ratio of administrative expenditure to grants/program expenditure.
US foundations	990 tax returns	Calculated the average across time of the ratio of the "cash basis" column of total operating and administrative expenses to grants paid.
CEPI	Board of directors report, 2016–2012	Calculated ratio of operating expenses to total expenditure.
The Global Fund	Annual report financial statements	Calculated ratio of operating expenses to grants disbursed.
Gavi	Annual report financial statements	Calculated ratio of management, general, and fundraising expenses to program expenses.
UNICEF	Annual report financial statements	Calculated ratio of program support to total expenditure (1990–2011). Calculated ratio of institutional budget to total program and emergency budgets (2012–2023).
UNFPA	Annual financial review	Calculated ratio of institutional budget to total expenses.
WHO	Financial report and annual financial statements	Calculated ratio of general operating expenses to total expenses.
Unitaid	Financial statement	Calculated ratio of operating expenses to program expenses.
USAID	US government budget database	Used outlays spreadsheet to calculate ratio of total outlays for USAID operating account to sum of outlays for bilateral accounts.
DFID	Annual report expense summary	Calculated ratio of DFID's administration expenses to DFID's bilateral program expenses from 2002 onward.
JICA	Statement of income in annual report	Calculated ratio of general administrative expenses to operating expenses less depreciation.
SIDA and other Swedish agencies disbursing aid	Swedish OpenAid website[91]	Calculated ratio of administrative costs to the sum of all program expenses.
NORAD and other Norwegian agencies disbursing aid	Norwegian Aid statistics website[92]	Calculated ratio of general administrative expenses to operating expenses less depreciation.
IDA	World Bank audited financial statements	Calculated ratio of management fee charged by IBRD to development credit disbursements.
IBRD	World Bank audited financial statements	Calculated ratio of administrative expenses to loan disbursements.
AfDB	Income statements in annual reports	Calculated ratio of administrative expenses to the sum of loan and grant disbursements.

ADB	Annual report	Calculated ratio of administrative expenses to the sum of loan and grant disbursements.
IDB	Annual report and financial statement	Calculated the ratio of administrative expenses to total loan disbursements.
EEA	Administrative budget data[93]	Calculated ratio of donor states' management budget and donor program partners' participation to net allocation to beneficiary countries.
PAHO	Audited financial statements	Calculated ratio of general operating expenses and contractual services to total expenses.
UNAIDS	Audited financial statements	Calculated ratio of general operating expenses to total expenses.

Figure S3.15 In-kind contributions by loan- and grant-making DAH channels of assistance

This figure illustrates the proportions of financial and in-kind DAH disbursed by loan- and grant-making institutions. The proportion of in-kind DAH varies, based on the channel. The overall proportion of in-kind DAH received across all channels has grown over time.

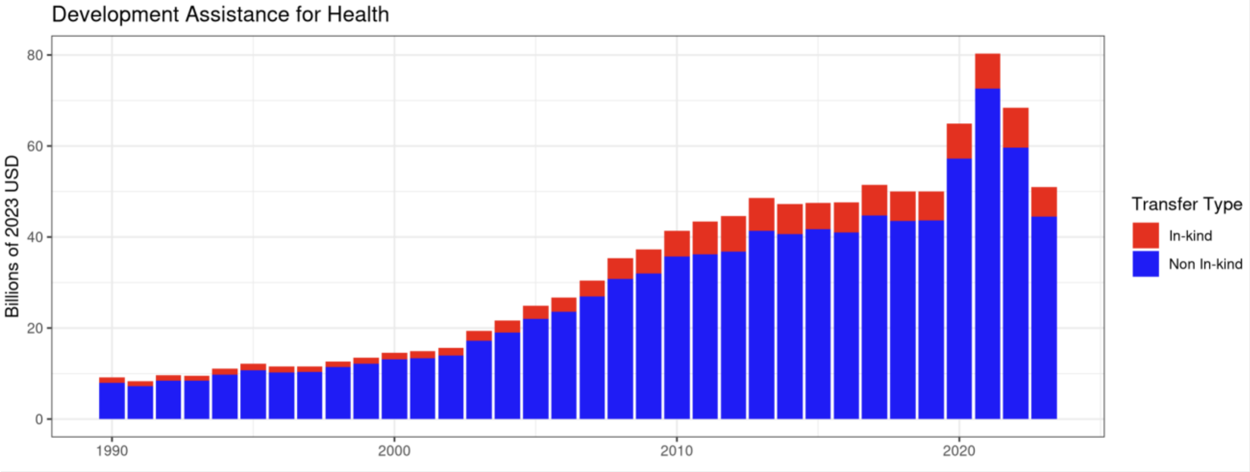

Source: IHME DAH Database (2024)

S3.10 REFERENCES

1 Dieleman JL, Schneider MT, Haakenstad A, *et al.* Development assistance for health: past trends, associations, and the future of international financial flows for health. *The Lancet* 2016; **387**: 2536–44.

2 OECD. International Development Statistics (IDS) online databases. https://www.oecd.org/en/publications/oecd-international-development-statistics_24142689.html (accessed May 9, 2025).

3 European Commission. Annual EU accounts and reports. https://commission.europa.eu/strategy-and-policy/eu-budget/how-it-works/annual-lifecycle/assessment/annual-accounts-and-reports_en (accessed May 23, 2025).

4 UNAIDS. PCB Archive. http://www.unaids.org/en/aboutunaids/unaidsprogrammecoordinatingboard/pcbmeetingarchive (accessed Jan 20, 2024).

5 UNICEF. UNICEF Strategic Plan: updated financial estimates, 2023-2026. 2023. https://www.unicef.org/executiveboard/documents/unicef-strategic-plan-updated-financial-estimates-2023-2026-srs-2023

6 UNICEF. United Nations Children's Fund Financial report and audited financial statements for the year ended 31 December 2023 and Report of the Board of Auditors. https://www.unicef.org/executiveboard/documents/unicef-financial-report-unboa-2023-frs-2025 (accessed May 23, 2025).

7 UNICEF. Annual Report 2023. https://www.unicef.org/reports/unicef-annual-report-2023 (accessed May 23, 2025). .

8 United Nations Population Fund. Financials and Annual Reports. https://www.usaforunfpa.org/financials-and-annual-reports/ (accessed May 23, 2025).

9 Unitaid. Audited Financial Statements. https://unitaid.org/publications/#en (accessed May 23, 2025).

10 Pan American Health Organization, World Health Organization. Executive Committee Session. 2023; published online Sep 29 2023. https://www.paho.org/en/documents/ce173fr-final-report (accessed Jan 20, 2024).

11 World Health Organization. Financial report and audited financial statement, 2023. https://www.who.int/publications/i/item/A77-20 (accessed May 23, 2025).

12 The World Bank. Projects & Operations. http://projects.worldbank.org/ (accessed Jan 20, 2024).

13 Paris M. Project database 1990–2022 obtained through personal correspondence. 2023.

14 African Development Bank. Compendium of statistics on bank group operations. Tunis, Tunisia: Statistics Department, African Development Bank.

15 African Development Bank. AfDB Annual Reports / Financial Reports. https://www.afdb.org/en/documents-publications/annual-report (accessed May 23, 2025).

15 African Development Bank. Online project database. https://www.afdb.org/en/projects-and-operations/project-portfolio/ (accessed Jan 20, 2024).

16 Inter-American Development Bank. Annual Reports. https://www.iadb.org/en/who-we-are/annual-reports (accessed May 23, 2025).

16 Inter-American Development Bank. Projects database. https://www.iadb.org/en/projects-search (accessed Jan 20, 2024).

17 Neret M. Health disbursement data. 2021; accessed Feb 7, 2022.

18 Gavi transparency portal and IATI. https://www.gavi.org/our-alliance/operating-model/gavi-transparency-portal-and-iati (accessed May 23, 2025).

19 Gavi, the Vaccine Alliance. Pneumococcal AMC. https://www.gavi.org/investing-gavi/innovative-financing/pneumococcal-amc (accessed Jan 20, 2024).

20 Gavi, the Vaccine Alliance. Financial reports. https://www.gavi.org/news-resources/document-library/financial-reports (accessed May 23, 2025).

21 Gavi, the Vaccine Alliance. Cash Receipts. https://www.gavi.org/investing-gavi/funding/donor-profiles/cash-receipts (accessed May 23, 2025).

22 The Global Fund. Grants in detail and Disbursements. https://data-service.theglobalfund.org/downloads (accessed Jan 20, 2024).

23 The Global Fund. The Global Fund annual reports. http://www.theglobalfund.org/en/archive/annualreports/ (accessed Jan 15, 2022).

24 The Global Fund. The Global Fund pledges & contributions report. https://data-service.theglobalfund.org/downloads (accessed Jan 15, 2022).

25 GuideStar USA. Income tax filings. http://www2.guidestar.org/Home.aspx (accessed Jan 27, 2022).

26 United States Agency for International Development. VolAg report: report of voluntary agencies engaged in overseas relief and development. https://www.usaid.gov/pvo/volag-report (accessed Jan 30, 2017).

27 Thomson Reuters. Red book expanded database. New York: Thomson Reuters, 2009.

28 World Health Organization. WHO | Essential medicines. WHO. http://www.who.int/topics/essential_medicines/en/ (accessed Feb 15, 2017).

29 GuideStar. Data Sets for Research. https://learn.guidestar.org/products/data-sets-for-research (accessed November 24, 2020).

30 Gates Foundation. Financials. https://www.gatesfoundation.org/about/financials (accessed May 23, 2025).

31 Chan S. Foundation Awards and Payments. 2016; published online Aug 16.

32 Foundation Center. Grants database. Candid. https://candid.org/ (accessed Jan 13, 2021).

33 EEA Grants-Norway Grants, Financial Mechanism Office. EEA Grants project portal. https://eeagrants.org/resources?title=&field_resource_type_target_id=109 (accessed Sept 17, 2020).

34 Popic A. EEA project data obtained through personal correspondence. Brussels, Belgium. EEA and Norway Grants. 2020.

35 Fofana A. AfDB development support for health projects enquiry. 2024; accessed Jan 1, 2025.

36 Rouzinova R. Incorporating UNITAID into Financing Global Health Landscape. 2017; published online Aug 21.

37 Al Anood Al Abdool. United Arab Emirates Ministry of Foreign Affairs and International Cooperation. UAE Foreign Assistance in Health 1990–2008 through personal correspondence. 2018; published online Jan 24.

38 Australian Government. Department of Foreign Affairs and Trade. Aid budget and statistical information. Dep. Foreign Aff. Trade. http://dfat.gov.au/aid/aid-budgets-statistics/Pages/default.aspx (accessed Dec 11, 2020).

39 Australian Government Department of Foreign Affairs and Trade. Australia's Official Development Assistance Budget Summary 2024-25. https://www.dfat.gov.au/about-us/corporate/portfolio-budget-statements/australias-official-development-assistance-budget-summary-2024-25 (accessed May 23, 2025).

40 Austria Federal Ministry of Finance. Federal budget. https://www.bmf.gv.at/themen/budget.html (accessed May 23, 2025).

41 Belgium House of Representatives. Budgets. https://www.lachambre.be/kvvcr/showpage.cfm?section=/pri/budget&language=fr&rightmenu=right_pri&story=2024-budget.xml (accessed May 23, 2025).

42 Government of Canada. Departmental plans: Global Affairs Canada. https://www.international.gc.ca/transparency-transparence/departmental-plan-ministeriel/index.aspx?lang=eng (accessed May 23, 2025).

43 Danish Ministry of Foreign Affairs. Foreign affairs budget. http://www.oes-cs.dk/bevillingslove/ (accessed May 23, 2025).

44 European Commission. EU spending and revenue 2021-2027. https://commission.europa.eu/strategy-and-policy/eu-budget/long-term-eu-budget/2021-2027/spending-and-revenue_en (accessed May 23, 2025).

45 Ministry of Finance Finland. State budget bills [in Finnish]. http://budjetti.vm.fi/indox/index.jsp (accessedMay 23, 2025).

46 Ministry of Foreign Affairs and International Development. Politique francaise en faveur du developpement. Ministry of Foreign Affairs and International Development, 2024 https://www.budget.gouv.fr/documentation/documents-budgetaires-lois/exercice-2024 (accessed May 23, 2025).

47 Legifrance. Republique Francaise. Budget and financial documents. https://www.legifrance.gouv.fr/initRechTexte.do (accessed Nov 15, 2020).

48 German Federal Ministry of Finance (Bundesministerium der Finanzen) 2024 Dashboard. https://www.bundeshaushalt.de/DE/Bundeshaushalt-digital/bundeshaushalt-digital.html (accessed May 23, 2025).

49 Greece Standing Committee on Economic Affairs. The state budget and budgets for certain special funds and services, 2013 and 2014 [in Greek]. 2014. http://www.hellenicparliament.gr/UserFiles/7b24652e-78eb-4807-9d68-e9a5d4576eff/Proyp2014-prak.pdf.

50 Hellenic Aid. Annual Reports. https://hellenicaid.mfa.gr/en/category/annual-reports/ (accessed May 23, 2025).

51 Department of Finance, Government of Ireland. Previous budgets. https://www.gov.ie/en/department-of-finance/collections/previous-budgets/ (accessed May 23, 2025).

52 Ministry of Foreign Affairs and International Cooperation. Stato di previsione del ministero degli affari esteri e della cooperazione internazionale. 2024. https://www.gazzettaufficiale.it/do/atto/serie_generale/caricaPdf?cdimg=24G00135001000101100009&dgu=2024-08-14&art.dataPubblicazioneGazzetta=2024-08-14&art.codiceRedazionale=24G00135&art.num=1&art.tiposerie=SG (accessed May 23, 2025).

53 Ministry of Finance Japan. Budget. https://www.mof.go.jp/english/policy/budget/budget/ (accessed May 23, 2025).

54 Korea Official Development Assistance. Comprehensive Implementation plan for international development cooperation by year. https://www.odakorea.go.kr/kor/bbs/YrlyIntrnDvcprPlan?bbs_id=kor_003 (accessed May 23, 2025).

55 Ministry of Finance Luxembourg. Pluriannual Budget. https://igf.gouvernement.lu/en/dossiers/budget-de-l-etat/budget-pluriannuel.html (accessed May 23, 2025).

56 New Zealand Treasury. Current and Past Budgets. https://www.treasury.govt.nz/publications/budgets/current-and-past-budgets (accessed May 23, 2025).

57 Ministry of Foreign Affairs Norway. Email correspondences. April 18, 2011, February 13, 2012, and August 14, 2013.

58 Norwegian Ministry of Finance. Statsbudsjettet 2024 (State Budget 2024). https://www.regjeringen.no/no/statsbudsjett/2024/id2994174/ (accessed May 23, 2025).

59 Ministry of Finance Portugal. State budget report. https://www.dgo.gov.pt/politicaorcamental/Paginas/OEpagina_ficheirosdeDados.aspx?Ano=2019&Tipo%20OE=OE%20Aprovado (accessed May 23, 2025).

60 Ministry of Finance and Public Function Spain. State General Budget [in Spanish] https://www.sepg.pap.hacienda.gob.es/Presup/PGE2024Prorroga/MaestroTomos/PGE-ROM/doc/L_24P_E_G2.PDF (accessed May 23, 2025).

61 Ministry of Foreign Affairs Sweden. International aid budget. https://www.regeringen.se/contentassets/e1afccd2ec7e42f6af3b651091df139c/utgiftsomrade-7-internationellt-bistand.pdf (accessed May 23, 2025).

62 UK FCDO annual report and accounts 2023 to 2024. https://www.gov.uk/government/publications/fcdo-annual-report-and-accounts-2023-to-2024 (accessed May 23, 2025).

63 Her Majesty's Treasury United Kingdom. Budget. https://www.gov.uk/government/publications/spring-budget-2018-documents (accessed Nov 15, 2020).

64 "Text - H.R.2882 - 118th Congress (2023-2024): Further Consolidated Appropriations Act, 2024." Congress.gov, Library of Congress, 23 Match 2024. https://www.congress.gov/bill/118th-congress/house-bill/2882/text (accessed May 23, 2025).

65 Centers for Disease Control and Prevention FY Congressional Justifications. https://stacks.cdc.gov/view/cdc/135997 (accessed May 23, 2025).

66 World Health Organization. Proposed programme budget 2024-2025. https://apps.who.int/gb/e/e_wha76.html (accessed May 23, 2025).

67 Joint United Nations Programme on HIV/AIDS. Financial report and audited financial statement. https://www.unaids.org/en/aboutunaids/unaidsprogrammecoordinatingboard/pcbmeetingarchive (accessed Jan 20, 2024).

68 Joint United Nations Programme on HIV/AIDS. Unified budget and workplan. https://www.unaids.org/en/aboutunaids/unaidsprogrammecoordinatingboard/pcbmeetingarchive/ (accessed Jan 20, 2024).

69 UNICEF. United Nations Children's Fund Financial report and audited financial statements for the year ended 31 December 2023 and Report of the Board of Auditors. United Nations: New York, 2024. https://www.unicef.org/executiveboard/documents/unicef-financial-report-unboa-2023-frs-2025 (accessed May 23, 2025).

70 Sabbah L. Health expenditure data - UNICEF. 2015; published online Sept 29.

71 UNFPA strategic plan, 2022-2025. https://www.unfpa.org/unfpa-strategic-plan-2022-2025-dpfpa20218 (accessed May 23, 2025).

72 UNFPA. Statistical and financial review, 2023. https://www.unfpa.org/statistical-and-financial-review-2023-dpfpa20244-part-iadd1 (accessed May 23, 2025). .

73 Smithson M, Verkuilen J. A better lemon squeezer? Maximum-likelihood regression with beta-distributed dependent variables. *Psychol Methods* 2006; **11**: 54–71.

74 Office of the European Union. EU budget 2018 Financial report. Luxembourg: Office of the European Union, 2015 https://commission.europa.eu/strategy-and-policy/eu-budget_en (accessed Jan 20, 2024).

75 President's Emergency Plan for AIDS Relief/ US Government. PEPFAR Dashboards- PEPFAR OU Budgets by Financial Classifications FY04-FY24. 2024. https://data.pepfar.gov/datasets (accessed Jan 6, 2025).

76 President's Malaria Initiative/US Government. Malaria Operational Plans (MOPs). 2024. https://www.pmi.gov/resources/malaria-operational-plans-mops/ (accessed Jan 20, 2024).

77 Miyuki Parris. IHME-WB FGH 2023 data request. 2022.

78 Projects & Operations - Sectors. https://projects.worldbank.org/en/projects-operations/project-sector (accessed May 23, 2025).).

79 Miyuki Parris. Project database 1990–2020 obtained through personal correspondence. Washington DC: The World Bank, 2021.

80 World Bank Group - International Development Association. Donor Contributions to IDA20 Replenishment. https://ida.worldbank.org/en/about/contributor-countries (accessed MAy 23, 2025).

81 Asian Development Bank. Online project database. https://www.adb.org/projects (accessed May 23, 2025).

82 African Development Bank Group. Resource Mobilization and Partnerships. https://www.afdb.org/en/topics-and-sectors/initiatives-partnerships/lusophone-development-compact-accelerating-sustainable-inclusive-private-sector-growth/partnerships (accessed Jan 20, 2024).

83 Asian Development Bank. Asian Development Fund 12 Donors' Report. https://www.adb.org/site/adf/replenishments (accessed Jan 20, 2024).

84 Gavi, the Vaccine Alliance. Annual financial reports. https://www.gavi.org/news-resources/document-library/financial-reports (accessed May 23, 2025).

85 World Health Organization. WHO Audited Financial Statements. https://www.who.int/about/accountability/financial-statements (accessed May 23, 2025).

86 Pan American Health Organization. Financial Report of the Director and Report of the External Auditor for 2023. https://www.paho.org/sites/default/files/2024-06/od370-e-financial-report-2023.pdf (accessed May 23, 2025).

87 Schlutter A, Volker T, Walkenhorst P. Foundations in Europe: International Reference Book on Society, Management and Law. Gutersloh, Germany; Washington, DC: Bertelsmann Stiftung; Brookings Institution Press, 2001.

88 European Economic Area. EEA grants report. Financial Instrument 1999-2003. Final report. http://eeagrants.org/content/download/10037/139187/version/3/file/141219+FI99-03+final+report.pdf.

89 European Economic Area. EEA report - Financial Mechanism 1994-1998. Final report. http://eeagrants.org/content/download/5915/65422/version/1/file/Financial+Mechanism+1994-1998+Final+Report.pdf.

90 European Economic Area. EEA Annual Report - 2016-2017. https://eeagrants.org/content/download/13045/175890/version/1/file/Annual+Report+2016-2017.pdf.

91 Swedish International Development Agency. Sweden's aid to the world for all sectors in. Openaid.se. https://openaid.se/en / (accessed May 23, 2025).

92 NORAD. Norwegian Aid Statistics. NoradDev. https://resultater.norad.no/en / (accessed May 23, 2025).

93 European Economic Area. Administrative budget - EEA Grants. https://eeagrants.org/Who-we-are/How-we-work/Administrative-budget (accessed Oct 27, 2020).

94 UNFPA. UNFPA Report on the Structured Funding Dialogues. https://www.unfpa.org/structured-funding-dialogues (accessed May 23, 2025).

95 World Health Organization. Status of collection of assessed contributions, including Member States in arrears in the payment of their contributions to an extent that would justify invoking Article 7 of the Constitution. https://apps.who.int/gb/e/e_wha77.html (accessed May 23, 2025).

96 World Health Organization. Voluntary contributions by fund and by contributor, 2023. https://apps.who.int/gb/e/e_wha77.html (accessed May 23, 2025).

97 Micah AE, Zhao Y, Chen CS, et al. Tracking development assistance for health from China, 2007–2017. BMJ Global Health 2019;4:e001513.

98 Lavado R.F. ADB development support for health projects enquiry. 2019; accessed Dec 19.

99 International Monetary Fund. World Economic Outlook Update, January 2025: Global GrowhtGrowth: Divergent and Uncertain. International Monetary Fund, 2025. https://www.imf.org/en/Publications/WEO/Issues/2025/01/17/world-economic-outlook-update-january-2025 (accessed Feb 7, 2025).

100 Organisation for Economic Co-operation and Development. Review of the ODA coefficients of select health-related organisations on Annex 2 and the Single Table of the Converged Statistical Reporting Directives for the Creditor Reporting System (CRS) and the annual DAC Questionnaire. chrome-extension://efaidnbmnnnibpcajpcglclefindmkaj/viewer.html?pdfurl=https%3A%2F%2Fwww.oecd.org%2Fofficialdocuments%2Fpublicdisplaydocumentpdf%2F%3Fcote%3DDCD%2FDAC%2FSTAT(2021)34%26docLanguage%3DEn&clen=596929&pdffilename=DCD-DAC-STAT(2021)34.pdf (accessed Jan 20, 2022)

101 The World Bank. Sector Taxonomy and definitions. Revised July 1, 2016. https://thedocs.worldbank.org/en/doc/538321490128452070-0290022017/original/NewSectorTaxonomyanddefinitions.pdf (accessed May 9, 2024).

102 The World Bank. Theme Taxonomy and definitions. Revised July 1, 2016. https://thedocs.worldbank.org/en/doc/275841490966525495-0290022017/original/NewThemeTaxonomyanddefinitionsrevisedJuly012016.pdf (accessed May 23, 2025).

103 Propublica Nonprofit Explorer. https://projects.propublica.org/nonprofits/ (accessed March 2024).

104 Silicon Valley Foundation. https://www.siliconvalleycf.org/ (accessed February 2024).

105 Conrad N. Hilton Foundation. https://www.hiltonfoundation.org/grants (accessed February 2024).

105 Good Ventures Foundation. https://www.goodventures.org/our-portfolio/grants-database/ (accessed February 2024).

106 The David and Lucile Packard Foundation. https://www.packard.org/grantees/search-our-grants/ (accessed February 2024).

107 The Leona M. and Harry B. Helmsley Charitable Trust. https://helmsleytrust.org/our-grants/ (accessed February 2024).

108 The Paul G. Allen Family Foundation. https://pgafamilyfoundation.org/ (accessed February 2024).

109 The Rockefeller Foundation. https://www.rockefellerfoundation.org/grants/ (accessed February 2024).

110 The William and Flora Hewlett Foundation. https://hewlett.org/grants/?sort=date (accessed February 2024).

111 International Aid Transparency Initiative. https://iatistandard.org/en/ (accessed May 9, 2025).

112 AidData. 2023. Global Chinese Development Finance Dataset, Version 3.0. Retrieved from https://www.aiddata.org/data/aiddatas-global-chinese-development-finance-dataset-version-3-0 (accessed Nov 19, 2024).

113 Gates Foundation. 2024 Gates Foundation Annual Letter. https://www.gatesfoundation.org/ideas/articles/2024-gates-foundation-annual-letter (accessed May 11, 2025).

114 Gates Foundation. 2023 Gates Foundation Annual Letter. https://www.gatesfoundation.org/ideas/articles/2023-gates-foundation-annual-letter (accessed May 11, 2025).

115 Gates Foundation. 2025, January 16. "Gates Foundation budget reaches $8.74B in 25th year, on track to meet $9B payout in 2026." https://www.gatesfoundation.org/ideas/media-center/press-releases/2025/01/annual-budget-name-change (accessed May 11, 2025).

116 Czech Aid. Plán činnosti ČRA na rok 2024. https://www.czechaid.cz/o-nas/zakladni-dokumenty (accessed May 23, 2025).

117 Estonia. 2024. aasta riigieelarve seadus (state budget act of 2024). https://www.riigiteataja.ee/akt/119122023019 (accessed May 23, 2025).

118 Hungary. Magyarország 2024. évi központi költségvetéséről szóló T/4181 számú törvényjavaslat (Bill No. T/4181 on the Central Budget of Hungery for 2024). https://www.parlament.hu/koltsegvetes-2024 (accessed May 23, 2025).

119 Iceland Financial Plan 2024-2028. https://www.stjornarradid.is/verkefni/opinber-fjarmal/fjarmalaaaetlun/fjarmalaaaetlun-2024-2028/ (accessed May 23, 2025).

120 Ministry of Finance of the Republic of Lithuania. 2024 m. patvirtintas biudžetas. https://finmin.lrv.lt/lt/veiklos-sritys/biudzetas/patvirtinti-biudzetai/2024-m-patvirtintas-biudzetas/ (accessed May 23, 2025).

121 Poland Ministry of Foreign Affairs. 2024 Development Cooperation Plan. https://www.gov.pl/web/polishaid/the-development-cooperation-plan-for-2024-approved (accessed May 23, 2025).

122 Slovakia. Zákon o štátnom rozpočte na rok 2024 (State Budget Act for 2024). https://www.slov-lex.sk/ezbierky/pravne-predpisy/SK/ZZ/2023/534/ (accessed May 23, 2025).

123 Slovenia. Sprejeti proračun Republike Slovenije za leto 2024 (Adopted Budget of the Republic of Slovenia for 2024). https://www.gov.si/teme/sprejeti-proracun/sprejeti-proracuni-republike-slovenije/sprejeti-prora/ (accessed May 23, 2025).

124 Federal Finance Administration of Switzerland. Data portal. https://www.efv.admin.ch/efv/en/home/finanzberichterstattung/daten/datencenter.html (accessed May 23, 2025).

125 Asian Development Bank. ADB Annual Reports. https://www.adb.org/documents/series/adb-annual-reports (accessed May 23, 2025).

S3.11 Tracking development assistance for health: incorporating COVID-19

During the rapidly evolving COVID-19 pandemic, we utilized sources additional to the ones mentioned above to obtain estimates of DAH for COVID-19 for each of our main channels. As time has gone on and more data becomes available, we have been able to integrate the tagging of DAH for COVID-19 into our main pipelines, and rely less on alternative data sources.

Table S3.11 Summary of COVID data sources by channel

Channel	Extracted from primary channel data source	IATI	UNOCHA	Correspondence	Channel website	Other website
AfDB	X			X		
AsDB	X			X		
Bilateral channels	X					
NGOs			X			
US foundations			X			X
UNICEF			X	X		
UNFPA			X			
UNAIDs				X		
Unitaid	X					
PAHO					X	

WHO					X	
IDB				X		
Gavi				X	X	X
The Global Fund					X	
Gates Foundation	X				X	
World Bank	X			X	X	
CEPI				X		
EC	X					

Bilateral agencies and European Commission

To estimate COVID-19 DAH for bilateral channels and EC, we tag all projects in the OECD CRS that have purpose name "COVID-19 control" as being for COVID-19. Additionally, we include projects that are in the emergency response sector but were tagged as related to COVID-19, as described further above in S3.2. Finally, we also include projects that have purpose name "Infectious disease control" and additionally are tagged with the keyword "#COVID-19" by the reporting agency.

UNAIDS

For UNAIDS, we received data from our contact Jose Antonio at the agency, on repurposed spending for COVID-19 for recipient countries for 2020. These data were used to inform COVID-19-related spending for the agency, and the repurposed money was removed from the total DAH envelope we estimated for 2020 to avoid double-counting. No keyword search was used on these data; all money was manually assigned to "Maintaining other essential health services and systems," since UNAIDS COVID spending was on maintaining existing programming in the face of the COVID-19 pandemic.

We did not have source information for this channel, and so categorized the source as "Unallocable."

We did not receive data after 2021 for UNAIDS and did not find disbursements through web scraping either. Therefore, we assumed there were no COVID-related disbursements through UNAIDS.

UNICEF

For the UNICEF channel, we identified UNOCHA as the most comprehensive location for UNICEF grant-specific data related to COVID-19.[2] We downloaded all columns and rows available in the database where UNICEF is the destination organization (Figure S3.10.1). To process these data, we subset on the Sector column to isolate "Health." From there, we checked for grants reporting multiple commitments or disbursements. If a grant had multiple rows reporting commitments and disbursements, we merged the rows and preferred disbursements over commitments.

In UNOCHA's data, there is a column reporting whether the grant is funded through new or repurposed money – repurposed money being "left-over" amounts intended for another use that has been redirected to

support the pandemic. In our existing data, we assume that we have already tracked all repurposed money, so in order to avoid double-counting, we reduced the envelope of our traditional UNICEF data by reallocating the health focus area assignments at the most granular level possible – in this case by donor country. After this adjustment, we are able to directly add the UNICEF COVID total amount onto our existing estimates where the repurposed-tagged money now exactly fills the reduction we made by donor country.

We also received correspondence data on vaccine delivery from UNICEF. These data were cleaned and incorporated into our model following the process detailed above.

Figure S3.10.1: Sample of UNOCHA's UNICEF health data[2]

3	Boundary	Source org.	Destination org.	Sector	Amount (US$)
85	Incoming	Asian Development Bank	United Nations Children's Fund	Health	$10,000,000
89	Incoming	Canada, Government of	United Nations Children's Fund	Health	$73,684
90	Incoming	Canada, Government of	United Nations Children's Fund	Health	$74,072
101	Incoming	World Bank	United Nations Children's Fund	Health	$3,869,071
102	Incoming	World Bank	United Nations Children's Fund	Health	$1,500,090
104	Incoming	World Bank	United Nations Children's Fund	Health	$195,300
105	Incoming	World Bank	United Nations Children's Fund	Health	$4,715,074
106	Incoming	World Bank	United Nations Children's Fund	Health	$884,258
109	Incoming	Ireland, Government of	United Nations Children's Fund	Health	$396,902
161	Incoming	United States of America, Government of	United Nations Children's Fund	Health	$389,194
250	Incoming	United States of America, Government of	United Nations Children's Fund	Health	$243,409
270	Incoming	Saudi Arabia (Kingdom of), Government of	United Nations Children's Fund	Health	$4,000,000
288	Incoming	World Bank	United Nations Children's Fund	Health	$384,894
315	Incoming	Yemen Humanitarian Fund	United Nations Children's Fund	Health	$15,999,547
317	Incoming	Japan, Government of	United Nations Children's Fund	Health	$1,084,166
320	Incoming	Ethiopia Humanitarian Fund	United Nations Children's Fund	Health	$25,920
329	Incoming	Canada, Government of	United Nations Children's Fund	Health	$102,799
398	Incoming	Ukraine Humanitarian Fund	United Nations Children's Fund	Health	$139,997
420	Incoming	United States of America, Government of	United Nations Children's Fund	Health	$1,000,000
542	Incoming	Japan, Government of	United Nations Children's Fund	Health	$2,712,528

UNFPA

For the channel UNFPA, we also used the UNOCHA Financial Tracking Service database to access data on the "COVID-19 Global Humanitarian Response Plan" for grant-specific data related to COVID-19.[2] We downloaded all the grants available in the database, selecting only those in the health sector where UNFPA is the destination organization. Both commitment and disbursement data were available – where there was grant overlap between the two, disbursement data only has been included, to avoid double-counting any grants. Where only commitment data were available, a 1:1 commitment to disbursement ratio was used to estimate disbursement spending. Source information was also available in the dataset.

Following the same strategy as for UNICEF, the value of the repurposed COVID-19 has been removed from our estimates for general UNFPA spending for 2020 and 2021.

Unitaid

For Unitaid, we received data from correspondence with Nargiza, Mazhidova, a Manager, Impact and Value for Money. This dataset included Unitaid grants, which had a variable tagging those that were COVID related. We followed the same data processing as our regular Unitaid channel pipeline and used the variable directly to determine what funds should count as COVID-19 DAH.

PAHO

We downloaded total contributions made to PAHO since the beginning of the COVID-19 pandemic from the PAHO COVID-19 Response Fund.[6] We extracted outbreak and crisis response expenditures from the 2022 financial report for the same year. We used this to determine the proportion of the PAHO envelope that contributed to COVID-19 DAH. .

WHO

For WHO, we downloaded the underlying data for the Contributions to WHO for COVID-19 visualizations, which report detailed information on the donors and partners of WHO's COVID-19 response (Figure S3.11.2).[8] These data contained donor information, distributed organization, pandemic flag (COVID-ACT vaccine-specific or COVID General) and assigned Strategic Preparedness and Response Plans (SPRPs) that we used for assigning program areas. We pulled recipient country information from the distributed organization variable, which combines the code for the WHO regional office as well as the ISO code of the recipient country. We dropped contributions where the source country was the same as the recipient country as we considered those domestic disbursements. We assigned funds going to headquarters and specific offices that did not indicate a recipient country as "Global" projects. To avoid double-counting WHO disbursements, we subtracted disbursements where the source was another one of the channels we track in the aggregation step of our COVID pipeline. For example, the African Development Bank provided money to WHO, which we later took out of our African development bank channel. We used the "Pandemic flag" variable to separate projects that were COVID vaccine focused with the label "COVID-ACT" from those in "COVID-General." We used the "distributed" variable as our commitment value and "award budget" as our disbursement value. We matched commitments and disbursements by donor, contributor, award ID, award type, and distributed organization since the data contained individual rows for commitments and disbursements..

S3.11.2: Sample of WHO's underlying COVID contributions data

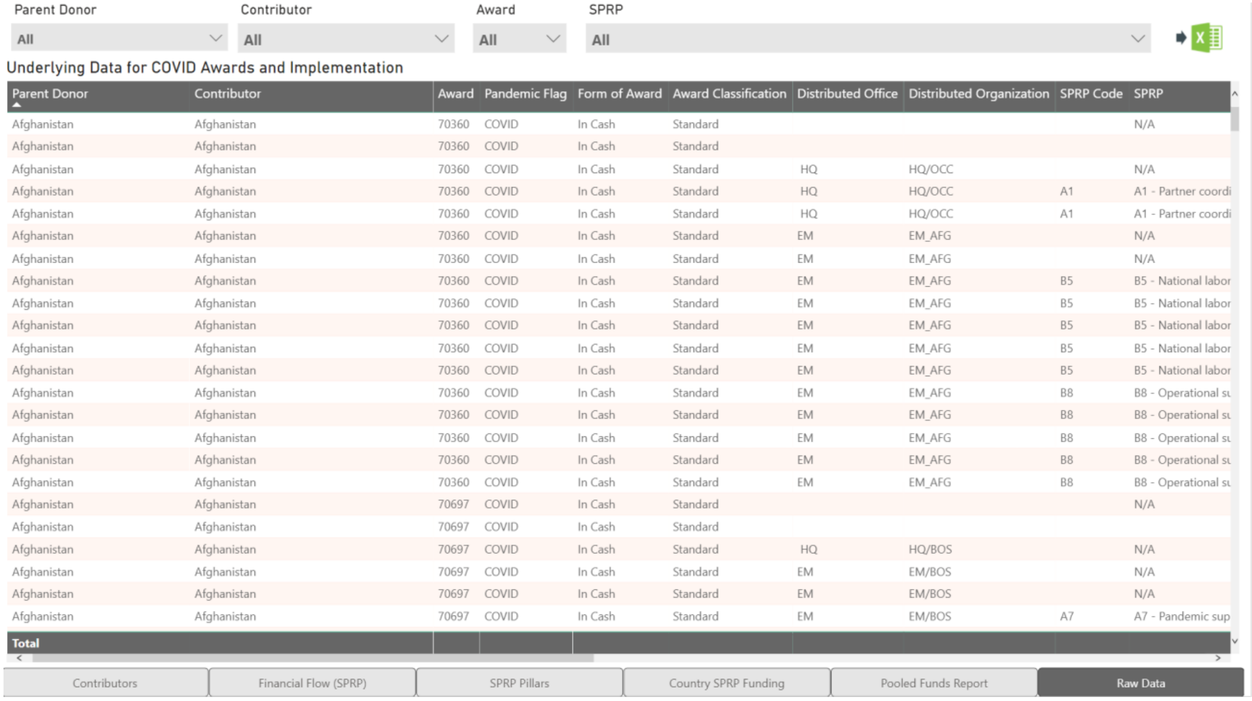

Parent Donor: All | Contributor: All | Award: All | SPRP: All

Underlying Data for COVID Awards and Implementation

Parent Donor	Contributor	Award	Pandemic Flag	Form of Award	Award Classification	Distributed Office	Distributed Organization	SPRP Code	SPRP
Afghanistan	Afghanistan	70360	COVID	In Cash	Standard				N/A
Afghanistan	Afghanistan	70360	COVID	In Cash	Standard				
Afghanistan	Afghanistan	70360	COVID	In Cash	Standard	HQ	HQ/OCC		N/A
Afghanistan	Afghanistan	70360	COVID	In Cash	Standard	HQ	HQ/OCC	A1	A1 - Partner coordi
Afghanistan	Afghanistan	70360	COVID	In Cash	Standard	HQ	HQ/OCC	A1	A1 - Partner coordi
Afghanistan	Afghanistan	70360	COVID	In Cash	Standard	EM	EM_AFG		N/A
Afghanistan	Afghanistan	70360	COVID	In Cash	Standard	EM	EM_AFG		N/A
Afghanistan	Afghanistan	70360	COVID	In Cash	Standard	EM	EM_AFG	B5	B5 - National labor
Afghanistan	Afghanistan	70360	COVID	In Cash	Standard	EM	EM_AFG	B5	B5 - National labor
Afghanistan	Afghanistan	70360	COVID	In Cash	Standard	EM	EM_AFG	B5	B5 - National labor
Afghanistan	Afghanistan	70360	COVID	In Cash	Standard	EM	EM_AFG	B5	B5 - National labor
Afghanistan	Afghanistan	70360	COVID	In Cash	Standard	EM	EM_AFG	B5	B5 - National labor
Afghanistan	Afghanistan	70360	COVID	In Cash	Standard	EM	EM_AFG	B8	B8 - Operational su
Afghanistan	Afghanistan	70360	COVID	In Cash	Standard	EM	EM_AFG	B8	B8 - Operational su
Afghanistan	Afghanistan	70360	COVID	In Cash	Standard	EM	EM_AFG	B8	B8 - Operational su
Afghanistan	Afghanistan	70360	COVID	In Cash	Standard	EM	EM_AFG	B8	B8 - Operational su
Afghanistan	Afghanistan	70360	COVID	In Cash	Standard	EM	EM_AFG	B8	B8 - Operational su
Afghanistan	Afghanistan	70697	COVID	In Cash	Standard				N/A
Afghanistan	Afghanistan	70697	COVID	In Cash	Standard				
Afghanistan	Afghanistan	70697	COVID	In Cash	Standard	HQ	HQ/BOS		N/A
Afghanistan	Afghanistan	70697	COVID	In Cash	Standard	EM	EM/BOS		N/A
Afghanistan	Afghanistan	70697	COVID	In Cash	Standard	EM	EM/BOS		N/A
Afghanistan	Afghanistan	70697	COVID	In Cash	Standard	EM	EM/BOS	A7	A7 - Pandemic sup
Total									

Contributors | Financial Flow (SPRP) | SPRP Pillars | Country SPRP Funding | Pooled Funds Report | Raw Data

World Bank

We received health-related disbursement data from correspondence with Miyuki Parris at the World Bank, and through extractions from IATI. This data included funding from both the IDA and IBRD branches for COVID-19 specific projects, which we were able to include in our standard channel pipeline for processing.

We further tracked projects from the World Bank Support for Country Access to COVID-19 Vaccines [24] to allocated appropriate updates to funding of existing projects or track projects primarily focused on COVID-19 vaccine efforts.

AfDB

For the African Development Bank (AfDB), we received data from contacts, Anliou Fofana, at AfDB, on all loans and grants distributed in 2023. Any loans or grants which reference COVID-19 in their title or description were selected. All of these loans and grants were classified by AfDb as "Multi-sector" and had both commitment and disbursement information. Reviewing each project (available on the website),[11,12] we attributed between 0% and 66% of each project's spending to health spending. Based on detailed project descriptions on their COVID-related projects website, we classified each project as a loan or grant.[11,12]

For the source information, we used replenishment data from the general DAH pipeline to inform proportions, and then applied those proportions to the reported COVID-19 health spending to estimate country-specific donations.

ADB

To estimate COVID-19 disbursements for 2020 and 2021, we utilize data received through correspondence for years 2020 and 2021. For years after 2021, we tag COVID-19 in the project-level data that is used in the channel's main pipeline.

IDB

For the Inter-American Development Bank (IDB), we received data from our contact Matilde Neret, Contract and Budget Officer at Inter-American Development Bank, on all health-related loans and grants disbursed. This data included indication of whether the project was focused on COVID-19, and what percentage of the project was focused on COVID-19.

Gavi

To estimate Gavi's DAH for COVID-19, we use a combination of public documents released by Gavi, including annual financial reports and cash receipts.[30,31] From Gavi's 2023 financial report, we obtain total cash disbursed by Gavi for COVAX AMC. Additionally, from Gavi's 2021 through 2023 financial reports, we obtain total COVID-19 vaccine doses donated to Gavi in each year. The sum of these sources in each year becomes our total COVID-19 DAH disbursed from Gavi, where we assume vaccine donations match vaccine disbursements. Also from these financial reports, we obtain cash and vaccine disbursements for COVAX by recipient country, which we use to disaggregate the total COVID-19 DAH by recipient. Additionally, from Gavi's 2024 statement of cash received, we extract cash received from

donors for COVAX AMC for years 2020-2023, which we use to disaggregate the total COVID-19 DAH by donor. We assume no COVID-19 DAH in 2024 or 2025, since COVAX closed at the end of 2023.

The Global Fund

For the Global Fund, we directly pulled country-specific disbursements and repurposed resources information from the "Funding Approved for COVID-19 Response" report published on the Global Fund website.[16] This dataset contains tables on country-specific new money disbursements awarded as well as country-specific repurposed money utilized.

Little formatting was needed for this source other than splitting regional and multi-country observations, where money was divided evenly between all countries represented in each region – country lists in each region were found using the Global Fund's data explorer.[17] For example, the Multicountry Americas (Andean) region was evenly split between Colombia, Ecuador, Peru, and Venezuela.

Like other channels, we made adjustments to our traditional Global Fund data to account for the repurposed money identified in our data source. The reallocation occurred at the recipient country level and was again filled by the tagged repurposed money after appending our Global Fund COVID dataset.

CEPI

For the CEPI channel, we received COVID-19 data through correspondence with Tuva Stolen and Mads Hogholen, the Head of Controlling and Director of Finance and Operations at CEPI, respectively. The dataset we received includes donor-specific COVID disbursement amounts which we confirmed with our contacts are all new money observations. After thoroughly reviewing CEPI's reported activities in 2020–2021 we deemed all COVID-19 spending as going to our R&D COVID program area because CEPI as an organization is focused on R&D, and lead on the COVAX facility on development of the vaccine. We then run our COVID-19 keyword search.

NGOs

For non-governmental organizations, we compiled data from UNOCHA's Financial Tracking System (FTS), which reports continuously updated data on humanitarian funding flows.[2] They receive report contributions from government donors, UN agencies, NGOs, and other humanitarian actors.[2] The data include project-level information, which included source organization, source type, recipient country, whether the money was new or repurposed, and year. We kept records where the listed recipient agency type included "NGO" and sectors that included "health," "multi-sector," "gender," "covid," "unspecified," or "child protection" in the sector title after reviewing projects from these sectors and determining they were DAH. If there were multiple sectors in a project, commitment and disbursements amounts were split equally among all sectors. The data contains separate rows for commitments, disbursements, and pledges from the same project. We match these projects with multiple rows to avoid double-counting. We used a 1:1 commitment to disbursement ratio to fill in missing disbursement information.

Gates Foundation

For the Gates Foundation, we identified COVID-related projects in the data received through correspondence.

China

For the China Bilateral channel, we used a three-tiered approach to estimating COVID contributions in 2020–2023. First, we pull data from UNOCHA where the source is the Chinese Government.[2] UNOCHA tags new money and repurposed commitment and disbursement observations, and grants with multiple observations of commitments or disbursements have been matched in an identical fashion to the UNICEF channel. Second, we identified our regular channel National Health Commission estimates as being entirely repurposed for COVID relief during 2020 and 2021; this assumption is based on the "White paper on China's action combating COVID-19, which said 56 medical teams that are abroad have assisted in COVID-19 response, including providing technical assistance and health education, and delivered over 400 training sessions,[19]this recommendation coming from an external FGH collaborator, Yingxi Zhao, who has worked with us previously on our estimation of China's bilateral contributions.[20] Finally, we calculated a back-of-the-envelope estimate of the Chinese government's global PPE, medical equipment, and additional medical team contributions using official press releases and the Duke University's Launch and Scale Speedometer, which we consider to be new money.

To format the UNOCHA data, we began by subsetting on the Sector column to isolate "Health." From there, we reformatted column data types to make manipulations simpler and checked for grants reporting multiple commitments or disbursements. If a grant had multiple rows reporting commitments and disbursements, we merged the rows and preferred disbursements over commitments as to not double-count spending. We then ran our COVID keyword search.

To process the NHC data, we extracted the value of DAH contribution from our regular channel data and appended it to our COVID data, and then passed it through our COVID keyword search. All NHC contributions were assumed to be medical workforce support, hence the reallocation as COVID-related contributions.

To calculate our back-of-the-envelope estimate of the Chinese government's global PPE, medical equipment, vaccine donation, and medical team contributions, we began by reviewing press releases from various Chinese ministries to gauge the number of batches of PPE, vaccine, and the number of medical teams sent globally. For 2020, we estimated that 34 additional medical teams were dispatched and 200 out of 283 batches of PPE were send to LMICs in 2020.[21,22] We then estimated a round unit cost per PPE/medical team batch, assuming that each batch contained roughly the same equipment, volume, and staffing. Finally, we multiplied the estimated number of batches by the estimated unit cost and report $60,000,000 as the total PPE, medical equipment, and medical team contributions. For 2021, we estimated that additional three medical teams were dispatched. As for vaccine donation, we estimated that 80 million vaccines have been delivered as of October 26, 2021.[25] This number was similar to Duke university's Launch and Scale Speedometer data, which suggested that China's vaccine donation for LMICs in 2021 was 71 million doses.[28] The vaccine's cost was based on official press release of China's donation to Laos, where 2.5 million doses of vaccines had an estimated worth of 80 million RMB – roughly 32 RMB per dose/5 USD per dose.[26] This is a lower estimate as compared with the official

market price from UNICEF COVID-19 Vaccine Market Dashboard, which reported the market price for China's vaccine around 20 USD per dose.[27] We multiplied the estimated number of doses with the unit cost estimate from Laos and report this as the cost of the Chinese government's vaccine donation contributions and labeled this as Vaccine commodity. As for recipient information of vaccine donation, we incorporated data from Duke University's Launch and Scale Speedometer data and allocated the remaining 9 million doses as "unallocable."

To account for repurposed money, we performed a similar reallocation of traditional channel money as noted in other channels – here by Chinese agency. We completely reallocated the 2020 and 2021 NHC traditional channel amount to COVID, and otherwise reallocated UNOCHA's reported repurposed money also by donor agency.

US foundations

We utilized purchased dataset from CANDID[23] and isolated USA originating grants funding for COVID-19 related purposes for 2020 and 2021. For 2022 and 2023, in lieu of purchasing the dataset form CANDID, we used the online tool from CANDID/The Foundation Center[29] to find COVID-19-related grants that went to each global region, assuming DAH based on region. We removed grants without health-related codes from the dataset. If a grant had an intended beneficiary, we labeled this as the recipient country or countries. If there was no intended beneficiary, we labeled the direct recipient country of the grant as the recipient country. If the grant identified more than one country as the recipient, we divided the funding equally between the recipients. We excluded high-income recipient countries.

We flagged recipient agencies that we track through other channels to avoid double-counting this funding. We flagged descriptions that specify any repurposing of funding as repurposed money.

References:

1. development-portal.org. http://d-portal.org/ctrack.html#view=search (accessed Jan 20, 2024).
2. Financial Tracking Service. https://fts.unocha.org/ (accessed Jan 20, 2024).
3. Austrian Development Agency. https://www.entwicklung.at/en/projects/all-projects (accessed Jan 20, 2024).
4. Version 1 Guidance Accessing and Using COVID-19 Data. Google Docs. https://docs.google.com/document/u/1/d/1hV_Bk2_VEQmrbSPbp3vE0Rho-gZtOJBSpLkOYInTnOY/edit?usp=embed_facebook (accessed Mar 18, 2022).
5. UNITAID. Unitaid and COVID-19 - Unitaid. https://unitaid.org/covid-19/#en (accessed Feb 2, 2022).
6. PAHO/WHO. COVID-19 Situation Reports. https://www.paho.org/en/covid-19-situation-reports (accessed Nov 10, 2021).
7. PAHO response to COVID-19 situation reportshttps://www.paho.org/en/topics/coronavirus-infections/coronavirus-disease-covid-19-pandemic (accessed Nov 17, 2021).
8. WHO. Contributions to WHO for COVID-19. https://app.powerbi.com/view?r=eyJrIjoiNzNmNTRkMWEtNmZjMS00NzdjLWEyMDYtYWExYzA4NzVhZGQwIiwidCI6ImY2MTBjMGI3LWJkMjQtNGIzOS04MTBiLTNkYzI4MGFmYjU5MCIsImMiOjh9 (accessed Jan 20, 2024).
9. The World Bank. Projects & Operations. World Bank. https://projects.worldbank.org/en/projects-operations/projects-home (accessed Jan 20, 2024).

10. The World Bank. World Bank Group's Operational Response to COVID-19 (coronavirus) – Projects List. World Bank. https://www.worldbank.org/en/about/what-we-do/brief/world-bank-group-operational-response-covid-19-coronavirus-projects-list (accessed Jan 20, 2024).
11. African Development Bank. AfDB Data Portal. https://projectsportal.afdb.org/dataportal/ (accessed Feb 09, 2022).
12. African Development Bank. Our COVID-19 response to date. African Development Bank - Building today, a better Africa tomorrow. 2020; published online June 24. https://www.afdb.org/en/news-events/our-covid-19-response-date (accessed Jan 20, 2024).
13. Asian Development Bank. Projects and Tenders. Asian Development Bank. https://www.adb.org/projects (accessed Jan 20, 2024).
14. Gavi, The Vaccine Alliance. COVID-19 situation reports. https://www.gavi.org/news-resources/document-library/covid-19-situation-reports (accessed Jan 20, 2024).
15. Gavi, The Vaccine Alliance. COVAX Facility. https://www.gavi.org/covax-facility (accessed Jan 20, 2024).
16. The Global Fund. COVID-19 Operational Response. https://www.theglobalfund.org/en/covid-19/ (accessed Jan 31, 2022).
17. The Global Fund. Map - The Global Fund Data Explorer. https://data.theglobalfund.org/investments/home (accessed Jan 20, 2024).
18. Bill & Melinda Gates Foundation. Grantmaking - Awarded Grants. https://www.gatesfoundation.org/about/committed-grants (accessed Jan 20, 2024).
19. The State Council, The People's Republic of China. China's action against the new coronavirus epidemic. http://www.gov.cn/zhengce/2020-06/07/content_5517737.htm (accessed Jan 20, 2024).
20. Micah AE, Zhao Y, Chen CS, et al. Tracking development assistance for health from China, 2007–2017BMJ Global Health 2019;4:e001513.
21. Xinhuanet. Xinhua International Current Review: The World Reverberation of the Great Anti-epidemic Spirit. http://www.xinhuanet.com/2020-09/09/c_1126473118.htm (accessed Jan 22, 2021).
22. People's Daily. Fully demonstrate China's conscious responsibility as a large responsible country (by everyone). http://paper.people.com.cn/rmrb/html/2020-09/24/nw.D110000renmrb_20200924_2-09.htm (accessed Jan 22, 2021).
23. Candid. Candid. https://candid.org/ (accessed Jan 10, 2022).
24. The World Bank. World Bank Support for Country Access to COVID-19 Vaccines – Projects List. World Bank. https://www.worldbank.org/en/who-we-are/news/coronavirus-covid19/world-bank-support-for-country-access-to-covid-19-vaccines (accessed Jan 20, 2024).
25. Science and technology daily. 2021. Understanding China's global response to the pandemic. http://www.stdaily.com/index/kejixinwen/2021-10/26/content_1228365.shtml (accessed Jan 20, 2024).
26. People's Government of Yunnan Province. China's donation of COVID vaccine and other materials to Laos. http://www.yn.gov.cn/ztgg/yqfk/fkyw/202111/t20211126_230887.html (accessed Jan 20, 2024).
27. UNICEF. Covid-19 vaccine market dashboard. https://www.unicef.org/supply/covid-19-vaccine-market-dashboard (accessed Jan 20, 2024).
28. Duke University. Duke Global Health Innovation Center. https://launchandscalefaster.org/covid-19/vaccinedonations (accessed Jan 20, 2024).

29. Foundation Center. https://maps.foundationcenter.org. (accessed February 2024).30. Gavi, the Vaccine Alliance. Annual Financial Reports. https://www.gavi.org/news-resources/document-library/financial-reports (accessed May 9, 2025).

31. Gavi, the Vaccine Alliance. Cash receipts. https://www.gavi.org/investing-gavi/funding/donor-profiles/cash-receipts (accessed April 25, 2025).

S3.11 Estimating Development Assistance for Health in 2025

Given the expected changes to the global health financing landscape in 2025, we set out to estimate 2025 DAH based on information gathered about cuts to global health funding and development assistance. As discussed above, many of our data sources are available through 2023 or 2024, and for those that require imputation in 2024, we are typically able to find budget documents or other forms of data to inform our estimates. However, producing 2025 estimates is a unique challenge because, with 2025 ongoing, these estimates must be predicted and should also reflect the significant changes to the funding of DAH.

To produce DAH estimates for 2025, we relied on public statements and news articles from and about the sources and channels we track. These sources provided information about the relative sizes of funding cuts to development assistance and/or global health programs, and in some cases specific funding levels. Using this data in combination with our final 2024 DAH estimates, we determined how the expected 2025 levels of DAH would compare to 2024 levels and then adjusted the 2024 estimates accordingly to produce the 2025 estimates. This results in a total DAH estimate for 2025 that is disaggregated by source and channel of funds.

In the table below, we highlight the main information and sources that informed our decision making, as well as our interpretation of the information and the final process used to arrive at the 2025 estimate. Note that, among the many assumptions made here, we often utilized cuts in total development assistance and assumed that they applied equivalently to development assistance for health. Additionally, for most other sources of DAH in our database, we hold their source-channel flows of DAH constant from 2024 to 2025. For many of these other sources, our research indicates that cuts are not expected, or at least not expected to impact 2025 DAH levels, so a last-value prediction is reasonable.

Table S3.12: Donor country cuts to development assistance for health in 2025

Donor Country	Relevant information	Source	Interpretation	Implemented Cuts for 2025 Estimate
US	Executive order pausing foreign aid; USAID foreign aid contracts cut including PEPFAR and PMI; USAID restructuring and downsizing; reduced IDA contributions; plans to cut funding to Gavi and UNAIDS, and withdraw from WHO; GFATM decrease; skinny budget includes significant cuts to foreign aid	Executive order (Jan 2025)[1]<br><br>Leaked Internal White House memo (Feb 2025)[2]<br><br>White House Discretionary Budget Request (May 2025) [3]	Many channels may see significant cuts as USAID is a key source of funds through both grants and contracts with other agencies. Explicit statements about the intention to end financial support for Gavi and UNAIDS, as well as the withdrawal from WHO, suggest cuts of 100% to these channels. To the World Bank IDA21 replenishment, the US had originally promised $4 billion under the previous administration, but the current administration plans to revise	US DAH to all channels other than the regional development banks and the below listed channels is decreased by 62% relative to 2024.<br><br>For Gavi, WHO, UNICEF, UNFPA, UNITAID, and UNAIDS, the US sourced DAH is dropped to $0.

		Budget rescission request (May 2025)[16]	this downwards by 20% to $3.2 billion. For Global Fund, the US's contributions have been on a downward trend and are expected to be impacted by the new administration's budget cuts. The latest potential cuts announced in the rescission request indicate complete cuts to UNFPA and UNICEF, among other UN agencies.	For World Bank IDA, US sourced DAH drops by 20%.<br><br>For Global Fund, we assume that US sourced DAH will be capped at $800 million.
UK	UK's aid budget to be reduced from 0.5% of gross national income to 0.3% by 2027	Statements from Sir Keir Starmer (Feb 2025)[4]<br><br>Proposed FCDO budget (Feb 2025)[5]	The UK's stated policy is to reduce its spending on foreign aid, so a decline is expected.<br><br>The FCDO spends the majority of UK's ODA. From the FCDO's statement from February 6, 2025, we have that the FCDO 2024-2025 ODA budget allocates £980,736,000 to health, which represents a notable decrease relative to the 2023-2024 budgeted level. While the FCDO's spending does not represent total UK ODA spending, it represents a notable share of estimated DAH.	UK DAH to all channels other than the development banks (including World Bank) is decreased so that total UK DAH in 2025 matches the FCDO budget. This results in a 42% decrease for all non-development bank channels.
Germany	Proposed budget included 8% reduction for the Development Ministry and 12% reduction to Federal Foreign Office, including a 53% reduction in humanitarian aid.	2025 Federal Draft Budget (Jul 2024)[6]<br><br>Government statements (Apr 2025)[7]	While still uncertain, it is expected that Germany will be reducing its ODA spending further in 2025, continuing its recent trend in tightening its aid budget.	Germany DAH to all channels other than the development banks (including World Bank) is decreased by 12% relative to 2024 levels.
France	France is planning to reduce public development aid by up to 40 percent	Proposed federal budget (Feb 2025)[8]	Based on the proposed federal budget, France's ODA is expected to drop significantly	France DAH to all channels other than the development banks (including

	as part of its €32 billion budget cuts for 2025.		in 2025, by about 37% relative to 2024.	World Bank) is decreased by 37% relative to 2024 levels.
Belgium	Belgium will cut its ODA budget by 25% over the next five years under a new coalition deal struck in January 2025.	Coalition budget (Jan 2025)[9]	Given the intention to cut its aid budget by 25% over the next five years, we assume that Belgium will cut ODA by roughly 5% each year, starting in 2025.	Belgium DAH to all channels other than the development banks (including World Bank) is decreased by 5% relative to 2024 levels.
Finland	Some 500 million euros will be cut from the budget for country and region-specific co-operation between 2024 and 2028.	Minister for Foreign Trade and Development press conference (Jan 2025)[10]	We assume that 500 million euros will be cut over the 5 years from 2024 through 2028, with roughly 100 million euros cut in 2025. Then we estimate the change in ODA relative to the initial 2024 ODA budget.	Finland DAH to all channels other than the development banks (including World Bank) is decreased by 12% relative to 2024 levels.
Netherlands	The current government plans to cut development aid by more than two thirds over the next three years, starting with a 300 million euro cut in 2025.	Government budget (Sept 2024)[11]	The expected $300 million euro cut represents about a 4.5% drop in the ODA budget, relative to the 2024 budget.	The Netherlands DAH to all channels other than the development banks (including World Bank) is decreased by 4.5% relative to 2024 levels.
Switzerland	The Swiss Agency for Development and Cooperation (SDC) has seen its budget cut from around CHF2.16 billion in 2024 to some CHF2.04 billion in 2025. Funding for all multilateral organisations, including the UN Development Programme (UNDP), the UN entity for gender equality and the empowerment of	2025 Federal budget (May 2024)[12]<br><br>UNAIDS announcement (January 2025)[13]	Based on the drop in the SDC's budget between 2024 and 2025, we expect ODA spending to decline accordingly.	Switzerland DAH to all channels other than the development banks (including World Bank) and UNAIDS is decreased by 5.56% relative to 2024 levels.<br><br>Switzerland DAH to UNAIDS is assumed to be $0.

	women (UN Women) and UNICEF has been cut by CHF30 million this year. Additionally, Switzerland has completely cut funding to UNAIDS.			

For Japan, the Ministry of Foreign Affairs has released an initial FY2025 ODA project budget which indicates a 2.2% increase in gross ODA, and thus we have applied a 2.2% increase to Japan's DAH through all channels it supports.[14] Similarly, we have found evidence of small increases for Australia (2.66%), Ireland (4.5%), and South Korea (3.8%), and have applied these increases to their bilateral channels.[17,18,19]

This process provides us with a complete set of donor-to-channel flows for 2025, which aggregate to a total 2025 DAH estimate. Figure S3.12.1 shows the percent change in DAH between 2024 and 2025 by source and channel (disbursing entity) of funds.

Figure S3.12.1

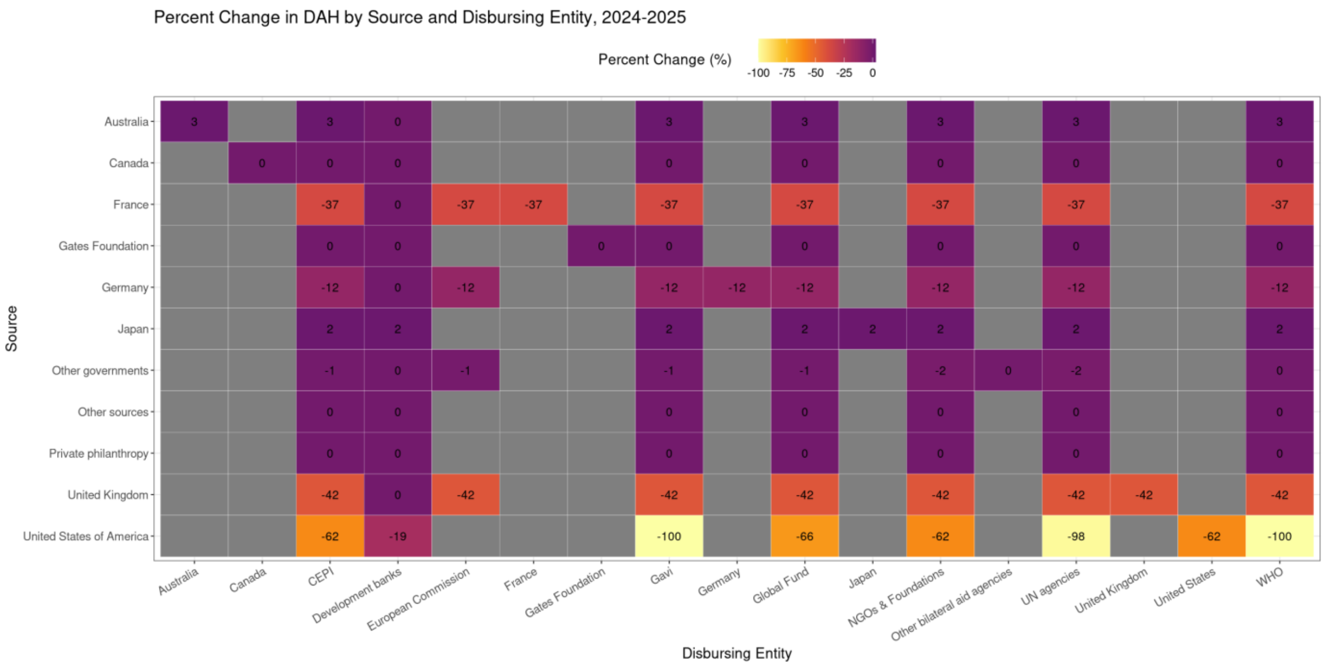

We consider the estimates described above to be a reference scenario for 2025 DAH levels. However, there is much uncertainty around the DAH funding cuts announced by certain donors. The cut to DAH sourced from the United States is expected to be significant, but the actual size of the cut will depend on complex decisions made by multiple branches of the country's government. The expected size of the US cut to its global health funding has changed significantly even over the relatively short period of time

since the relevant policies were announced. Therefore, we sought to explore alternative scenarios for 2025 DAH based on different US DAH cut levels. In the reference scenario described in Table S3.12, we cut US DAH to bilateral and some multilateral channels by 62% relative to 2024, with other channels receiving smaller or larger cuts. We also considered a worse-case scenario where the 62% cuts were replaced with 80% cuts, because initial reports indicated that over 80% of USAID programs would be cancelled.[15] Additionally, we considered a better-case scenario where the 62% cuts were replaced with 9% cuts, to reflect the rescission request proposed on May 28, 2025.[16] In this scenario, we assume that other drastic proposals made by the US government are not realized during 2025, and instead rely primarily on the cuts to global health programs proposed in the rescission request. Figure S3.12.2 shows the different 2025 DAH estimates for the United States based on each scenario.

Figure S3.12.2

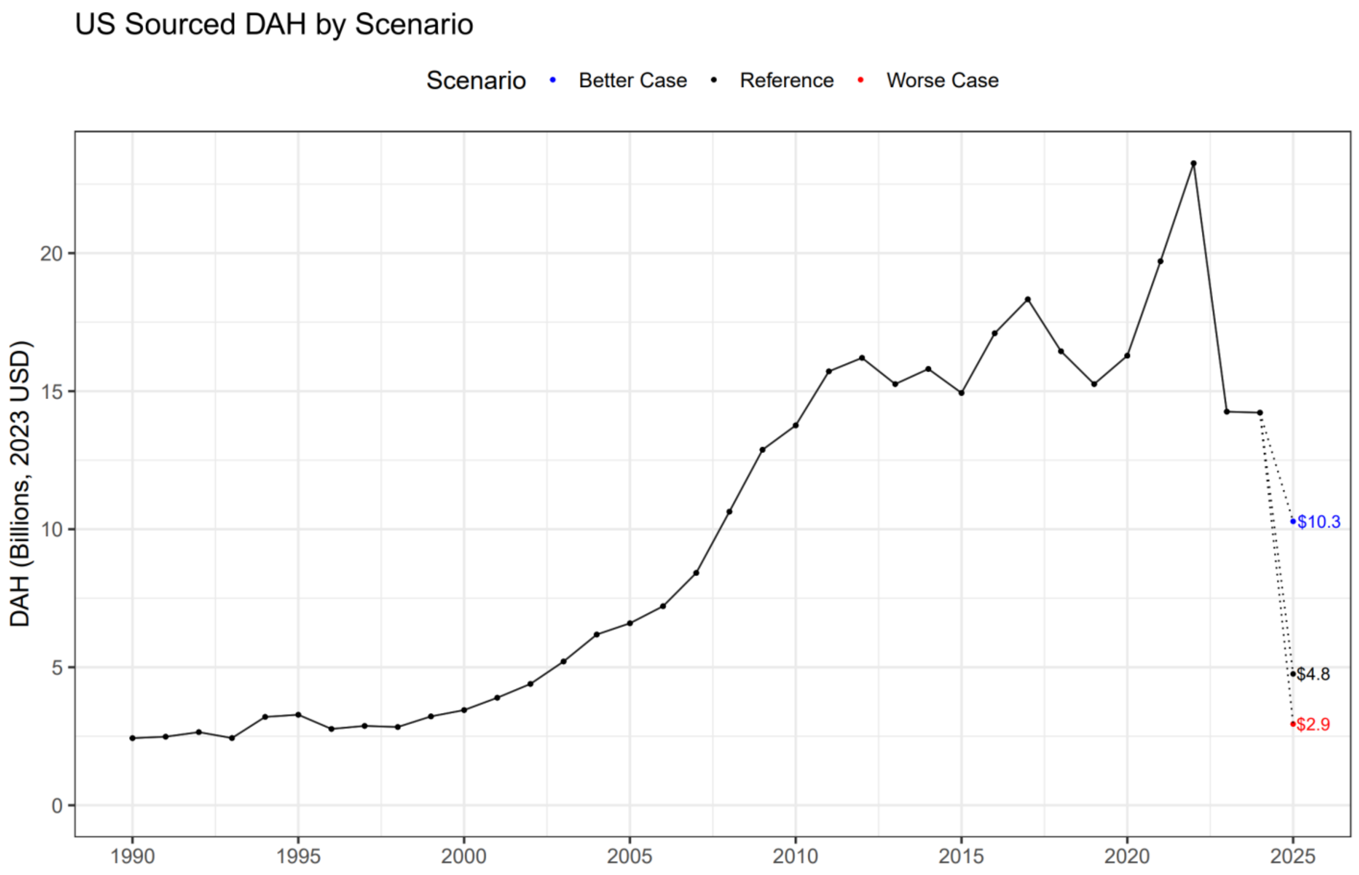

References:

1. The White House. 2025, January 20. “Reevaluating and realigning United States foreign aid.” https://www.whitehouse.gov/presidential-actions/2025/01/reevaluating-and-realigning-united-states-foreign-aid/ (accessed May 9, 2025).
2. Bateman, Tom. 2025, January 24. “US orders immediate pause to foreign aid, leaked memo says.” BBC. https://www.bbc.com/news/articles/ce9nx5k7lv0o (accessed May 9, 2025).

3. The White House. 2025, May 2. Fiscal Year 2026 Discretionary Budget Request. https://www.whitehouse.gov/wp-content/uploads/2025/05/Fiscal-Year-2026-Discretionary-Budget-Request.pdf (accessed May 9, 2025).
4. Hatch, Jonathan. 2025, February 25. "Prime Minister Keir Starmer announces cut to Official Development Assistance (ODA) to fund defence – what we know so far." https://www.bond.org.uk/news/2025/02/prime-minister-kier-starmer-announces-cut-to-official-development-assistance-oda-to-fund-defence-what-we-know-so-far/ (accessed May 9, 2025).
5. Dodds, Anneliese. 2025, February 6. "Foreign, Commonwealth and Development Office Official Development Assistance (ODA) programme allocations, 2024-25". https://questions-statements.parliament.uk/written-statements/detail/2025-02-06/hcws421 (accessed May 9, 2025).
6. Donor Tracker. 2024, July 17. "German government agrees on draft budget for 2025; ODA funding envelopes significantly cut." https://donortracker.org/policy_updates?policy=german-government-agrees-on-draft-budget-for-2025-oda-funding-envelopes-signficantly-cut-2024 (accessed May 9, 2025).
7. Chase-Lubitz, Jesse. 2025, April 10. "Germany's coalition contract includes new cuts to aid budget". Devex. https://www.devex.com/news/germany-s-coalition-contract-includes-new-cuts-to-aid-budget-109837 (accessed May 9, 2025).
8. Hird, Alison. 2025, May 2. "France's proposed budget cuts set to slash overseas development aid." RFI. https://www.rfi.fr/en/france/20250205-france-proposed-budget-cuts-slash-overseas-development-aid-coordinationo-sud (accessed May 9, 2025).
9. Chadwick, Vince. 2025, February 10. "Belgium just cut its foreign aid by 25%. Does anybody care?" Devex. https://www.devex.com/news/belgium-just-cut-its-foreign-aid-by-25-does-anybody-care-109320 (accessed May 9, 2025).
10. Kliemann, Christiane. 2024, September 17. "Finland's Development Aid Plunges amid Far-Right Take-Over of Policy Agendas." European Association of Development Research and Training Institutes. https://www.developmentresearch.eu/?p=1961(accessed May 9, 2025).
11. Meijer, Bart H. 2024, September 17. "Dutch right-wing government cuts development aid as deficit balloons." Reuters. https://www.reuters.com/world/europe/dutch-right-wing-government-cuts-development-aid-deficit-balloons-2024-09-17/#:~:text=The%20government%20said%20it%20aims,3.5%20billion%20euros%20this%20year (accessed May 9, 2025).
12. Allen, Matthew. 2025, February 7. "Foreign aid cuts: where does Switzerland stand?" swissinfo.ch. https://www.swissinfo.ch/eng/foreign-affairs/foreign-aid-cuts-where-does-switzerland-stand/88841450 (accessed May 9, 2025).
13. Langrand, Michael. 2025, January 29. "Switzerland to slash funding for UN agencies." Geneva Solutions. https://genevasolutions.news/global-news/switzerland-to-slash-funding-for-un-agencies (accessed May 9, 2025).
14. Japan Ministry of Foreign Affairs. Overview of FY2025 ODA Project Budget (Initial Budget) and Its Financial Sources (in Japanese). https://www.mofa.go.jp/mofaj/gaiko/oda/files/100781983.pdf (accessed May 20, 2025).
15. Pamuk, Humeyra. 2025, March 10. "Trump administration scraps over 80% of USAID programs, top diplomat Rubio says." Reuters. https://www.reuters.com/world/us/trump-administration-scraps-over-80-usaid-programs-top-diplomat-rubio-says-2025-03-10/ (accessed May 27, 2025).

16. The White House. 2025, May 28. Proposed rescissions of budgetary resources. https://www.whitehouse.gov/wp-content/uploads/2025/03/Proposed-Rescissions-of-Budgetary-Resources.pdf (accessed June 6, 2025).
17. Australia Department of Foreign Affairs and Trade. “Australia's Official Development Assistance Budget Summary 2025-26.” https://www.dfat.gov.au/about-us/corporate/portfolio-budget-statements/australias-official-development-assistance-budget-summary-2025-26 (accessed June 6, 2025).
18. Ireland Department of Foreign Affairs and Trade. 2024, October 1. “Tánaiste welcomes budget to strengthen Ireland’s global influence and impact.” https://www.gov.ie/en/department-of-foreign-affairs/press-releases/t%C3%A1naiste-welcomes-budget-to-strengthen-irelands-global-influence-and-impact/ (accessed June 6, 2025).
19. Gahui, Kang. 2025, February 21. “2025 official development aid raised 3.8% to KRW 6.5T.” https://www.korea.net/NewsFocus/policies/view?articleId=266927 (accessed June 6, 2025).

Forecasting Health Spending from 2023 through 2030

Supplementary Appendix

Global Burden of Disease Financing Global Health Collaborator Network

Updated: Tuesday 27th May, 2025

Contents

S4.0 Projecting development assistance from 2026 through 2030 2

S4.1 Data 3
- S4.1.1 Summary of Data Sources 3
- S4.1.2 World Health Organization's Global Health Expenditure Database 3
- S4.1.3 WB WDI, IMF WEO, PWT, and Maddison 4
- S4.1.4 Institute for Health Metrics and Evaluation's Development Assistance for Health Database 4
- S4.1.5 Institute for Health Metrics and Evaluation's Global Burden of Disease Study 2021 5

S4.2 Covariates 6
- S4.2.1 List of Forecasted Variables 6
- S4.2.2 Covariates Used for Forecasting 6

S4.3 Ensemble Modeling 8
- S4.3.1 Sub-model Setup 8
- S4.3.2 Covariates 8
- S4.3.3 Convergence to Global Growth Rates 9
- S4.3.4 Specifications 9

S4.4 Inclusion and Exclusion Criteria 11

S4.5 Creating the Forecasts 12
- S4.5.1 Ranking Sub-Models 12
- S4.5.2 Uncertainty Estimation 12

S4.6 Ad-hoc Draws Correlation 14
- S4.6.1 Motivation 14
- S4.6.2 Bivariate Correlated Distributions 14

S4.7 Future Health Scenarios 14
- S4.7.1 Long-term Growth Regressions 14
- S4.7.2 Forecasting Greater and Lesser Scenarios 15
- S4.7.3 Uncertainty Estimation 16

S4.8 Package and Architecture 17
- S4.8.1 Architecture 17
- S4.8.2 Template Model Builder 17

References 18

S4.9 Tables and Figures 19

Introduction

The objective of this study is to provide data on future health spending patterns that can guide decision-makers. These analyses produced comprehensive and comparable set of gross domestic product and all-sector government spending estimates, followed by all the components of a country's total health expenditure. Additionally, using observed past trends, we predicted the possible trajectories of the covariates of interest conditional on following an optimistic and pessimistic pattern based on global rates of change.

The purpose of this appendix is to describe in detail the methodology used in our analyses. Subsequent sections contain information on all data sources, ensemble forecasting strategies, inclusion criteria, and uncertainty estimation used to generate our estimates, as well as our guidelines on how our future health scenarios analyses were conducted.

SECTION 4. FORECASTING HEALTH SPENDING FROM 2023 through 2030

S4.0 Projecting development assistance from 2026 through 2030

To project development assistance for health donated in the long term, we used information on donors' targets for ODA funding.

For donors that use ODA per GNI targets to determine their ODA funding amounts, we collected information on their funding targets. These donors include Australia, Germany, Denmark, Spain, France, United Kingdom, Ireland, Italy, Luxembourg, Netherlands, and Norway. We first projected GNI by obtaining retrospective data from 1960 to 2023 from the World Bank World Development Indicators Database, and fit a linear model with GNI per capita as the dependent variable and GDP per capita as the independent variable, then projected GNIpc as a function of our GDPpc forecasts. We then multiplied GNIpc by IHME population estimates to obtain total GNI forecasts. For countries with targeted ratios of ODA per GNI, we applied these ratios to the forecasts of GNI to obtain ODA forecasts. We then extracted DAH per ODA data from the OECD CRS database for each donor, assuming a constant ratio from the last observed year will be used in future years. We then multiplied this ratio by the ODA forecasts to obtain total DAH forecasts for each donor. For a few donors including Australia, Germany, Denmark, Ireland, Luxembourg, and Norway, we assumed a constant ODA per GNI ratio, based on stated commitments of countries' governments to maintain their prioritization of development assistance. Many donors have announced ODA budget cuts; including the United Kingdom, which plans to maintain their ODA per GNI ratio at 0.5% in 2026 and further declining to 0.3% from 2027 onward, The Netherlands which has announced budget cuts to reduce ODA per GNI from 0.62% in 2024 to 0.44% by 2029, and France which has historically targeted the 0.7% ratio by 2030 but these targets have been pushed back and are now being maintained closer to 0.45% ratio. Some donors have countered these trends by committing to increasing their ODA per GNI target, including Spain and Italy, who have announced ambitious goals to reach the 0.7% ODA per GNI target as set forth by the UN by 2030.

For donors that do not use ODA per GNI targets, we held constant their DAH from 2026 to 2030 using the DAH levels in 2025. These include Austria, Belgium, Canada, Switzerland, China, Finland, Greece, Japan, South Korea, New Zealand, Portugal, Sweden, and the United States, and the Gates Foundation. Additional donor sources such as other OECD DAC countries, debt repayments, other public donors, private donors, and unallocable donors, we created projections using a linear model based on the historic time trend of DAH donated.

To create "better" and "worse" scenarios of forecasted health spending, we assumed a 0.7% ODA per GNI ratio beginning in 2026 in the "better" case, and a 0.1% ratio in the "worse" case.

Finally, to create forecasts of DAH by recipient, we projected the fraction of the total DAH envelope donated that was received by each recipient, net of US bilateral channel funding, and we used our ensemble forecasting methods described in section 4 to estimate this.

S4.1 Data

S4.1.1 Summary of Data Sources

We used data from seven sources for the analyses:

- World Health Organization's (WHO) Global Health Expenditure Database January 2025 database
- World Bank (WB) World Development Indicators (WDI) December 2024 database
- International Monetary Fund's (IMF) World Economic Outlook (WEO) April 2025 database
- United Nations Statistics Division National Accounts Main Aggregates Database December 2024 database (UN)
- Penn World Tables 10.01 (PWT)
- Angus Maddison Project Database 2020 (Maddison)
- Institute for Health Metrics and Evaluation's Development Assistance for Health Database 2024 (IHME)
- Institute for Health Metrics and Evaluation's Global Burden of Disease Study 2021 (IHME GBD 2021)

Specifically, we collected health expenditure information on all available sources that is comparable across countries and complete for most countries from WHO and IHME, and demographic data from the WPP, while the underlying data for producing gross domestic product (GDP) and general government expenditure (GGE) were extracted from the IMF, WB, UN, PWT, and Maddison. Debt ratio data including payments on interest, debt, and GGE are sourced from the IMF WEO database. Table S4.1 presents the definitions for the various health expenditure sources.

S4.1.2 World Health Organization's Global Health Expenditure Database

We used Global Health Expenditure Database (GHED) data from the WHO to generate our estimates, which spans 192 countries between 2000 and 2022 (in the January 2025 version). From the GHED, we pulled the following variables:

(i) Compulsory prepayment (Other, and unspecified, than FS.3)

(ii) Other revenues from NPISH n.e.c.

(iii) Other revenues from corporations n.e.c.

(iv) Other revenues from households n.e.c.

(v) Social insurance contributions

(vi) Transfers from government domestic revenue (allocated to health purposes)

(vii) Gross Domestic Product

(viii) Voluntary prepayment

To ensure we were using the best possible data, we downloaded the metadata for each data point for all of the indicators from the GHED website. We used the metadata to decide how each given data point should be weighted, from 1 to 5, being applied as inverse variance weights. We used a natural language processing model to evaluate and assign a weight to each datapoint based on the metadata describing the source and methods used to estimate those values in the GHED. Weights were based upon metadata completeness, documented source information, and documented methods for estimation. Details of the weighting guidelines and classification are explained in the supplementary appendix of our retrospective analysis paper. We adjusted these data by converting them from current local currency to 2023 US dollars.

Once we have an incomplete set of data points for the health expenditure variables we are interested in forecasting, we used Spatiotemporal Gaussian process regression (ST-GPR) to model the full time-series for each variable across a total of 204 countries. ST-GPR is a stochastic modeling technique that is designed to detect signals amidst noisy data. Unlike classical linear models that assume that the trend underlying data follows a definitive functional form, GPR assumes that the specific trend of interest follows a Gaussian Process, existing with some pointwise mean and covariance function [1]. The covariates that were used in order to determine the initial fit of our health expenditure variables are: lag-distributed income, all-sector government expenditure per capita, and proportion of total population over the age of 64; using a Matérn covariance function for the distribution of the Gaussian process. Once we determined an initial prediction of our dependent variables, using the variability of data across regions, Gaussian process regressions (GPR) were run in order to estimate 500 draws of each country-year estimate per metric.

S4.1.3 WB WDI, IMF WEO, UN, PWT, and Maddison

The WDI Database provides data on a wide range of development related variables, including data on GDP and GDP per capita. Data series in this database begin in 1960 and span through the present. The IMF's WEO Database provides data on various macroeconomic indicators. Macroeconomic series data are available from 1980 to present. The PWT is a database that provides real national accounts data for 183 countries and territories from 1950 to present. The Maddison Project database provides historical GDP, GDP per capita, and population data dating as far back as Roman times. The UN Statistics Division also provides data on GDP for 220 countries from 1970 to present. We utilized GDP per capita as a primary covariate to produce forecasts. GDP per capita from 1950 through 2024 was constructed using the method described in *James et al* [2]. The method utilized extracted data from a number of sources (IMF, WB, UN, PWT and Maddison), and used multiple random effects models to estimate a mean GDP per capita series to be used in our analysis. Similarly, we used the same methodology to produce a mean general government expenditure (GGE) per GDP series, from 1980 through 2024.

S4.1.4 Institute for Health Metrics and Evaluation's Development Assistance for Health Database

Development assistance for health estimates were obtained from the Institute for Health Metrics and Evaluation's Development Assistance for Health Database. To generate these estimates, IHME collected audited budgets, annual reports, and project records from the primary development agencies providing assistance for the health sector. These records are augmented by information acquired via correspondence, and are standardized and compiled to provide a comprehensive perspective on international financial flows for health. These estimates are tracked backward to the source of the funds and forward to the country recipient, and are available from 1990 through 2025 and 2023, respectively.

S4.1.5 Institute for Health Metrics and Evaluation's Global Burden of Disease Study 2021

Covariates used in forecasting health spending, such as populations and educational attainment per capita [3, 4], were obtained from the Institute for Health Metrics and Evaluation's Global Burden of Disease Study 2021. Population forecasts used were from IHME's Future Health Scenarios work, which uses a cohort component method of projection (CCMP [5]), and takes forecasts of age-specific fertility rates, mortality, migration, and the sex ratio at birth as inputs [6]. Age-specific fertility forecasts were projected by modeling completed cohort fertility as a function of met need for contraception and maternal education. Completed cohort fertility forecasts were then converted back to age-specific fertility rates. Net migration rates were forecasted as a function of the sociodemographic index, the crude population growth rate, and mortality due to conflict, war and natural disasters. Total (all-cause) mortality was derived from forecasting cause-specific mortality from GBD 2021, using the three-component model described in Foreman et al [4]. Cause-specific mortality was then aggregated up the GBD 2021 cause hierarchy to generate total mortality forecasts, which were utilized to compute future life tables. Using past population from GBD 2021, the CCMP model advanced populations forward and produced country-age-sex specific estimates for single future calendar years.

S4.2 Covariates

S4.2.1 List of Forecasted Variables

The following are the list of variables which are forecasted in the manuscript:

- GDP: Gross Domestic Product (national income of a country)
- GGE: General Government Expenditure (all sector government expenditure in a country)
- Debt ratio: GGE net interest repayments on debt, per GGE
- DAH_d: Development Assistance for Health donated
- DAH_r: Development Assistance for Health received

To generate estimates of future health spending, we forecasted gross domestic product, general government spending (across all sectors), debt ratio, total DAH donated and received through 2030. We used ensemble models to forecast these indicators and included multiple submodels spanning a number of covariates and model specifications. We then selected the best-performing set of submodels based on out-of-sample tests and used those submodels to create forecasts with 500 draws for each indicator.

To forecast total DAH provided, we used the ensemble models to first forecast debt as a proportion of general government spending, then we multiplied that ratio by total government spending to produce forecasts of total government spending net debt, for each country and year. Then, we used estimates from the IMF World Economic Outlook of official development assistance (ODA) as a fraction of general government spending, and official development assistance for health as a fraction of official development assistance, to create forecasts of official development assistance for health for each donor country and year. We adjusted our forecasts of ODA and DAH for 2023–2025 to reflect recent reports on donor funding where available.

S4.2.2 Covariates Used for Forecasting

The following covariates are used as predictors, or independent variables, in our models:

- Total population of a country (1950 - 2030)
- Proportion of total population below the age of 15 (1950 - 2030)
- Proportion of total population above the age of 64 (1950 - 2030)
- Educational attainment per capita above the age of 15 (1980 - 2030)
- An indicator variable used to denote the Ebola crisis in Guinea, Sierra Leone and Liberia in our DAH_r forecasts (1 for years in 2014 and 2015 for the three aforementioned countries, 0 otherwise).

Additionally, we also use the forecasted GDP and GGE per capita as covariates to predict the health expenditure variables. Figure S4.1 in the Tables and Figures section shows our full pathway for forecasting all our endogenous variables and how each of those variable fed into a succeeding model.

In order to forecast GDP per capita, we first convert GDP per capita to GDP per working population in the following manner:

$$\text{GDP}_{\text{working population}} = \text{GDP}_{\text{total population}} \times \frac{\text{Working population (20-64 years old)}}{\text{Total Population}}$$

We defined working age population as ages 20-64; this age group was chosen because it yielded the best model fit in tests, which also considered a minimum working age of 15 years and a maximum working age of 70 years. We forecast GDP per working population from 2025-2030 and convert back to GDP per capita for further analysis. The out-of-sample root-mean-squared-error (RMSE) based on using GDP per working population was smaller than forecasting with GDP per capita.

S4.3 Ensemble Modeling

The purpose of ensemble modeling is to make sure that we capture the most out of what we have in our arsenal in terms of covariates and model specifications. We are agnostic about one model being the sole predictor of the future, and allow an ensemble of beliefs about predicting off of the past trends. 'Ensembling', in simple terms, is a way of pooling a number of sub-models, where the space of sub-models span different inclusions and combinations of predictors, and/or different econometric specifications.

S4.3.1 Sub-model Setup

Our basic sub-model is a linear mixed effect model of the following form, for country i and time t:

$$\Delta_t Y_{i,t} = (\alpha + \alpha_i) + \left(\sum_{p=1}^{3} \rho_p \Delta_t Y_{i,t-p}\right) + \Delta_t X' \beta + \varepsilon_{i,t} \tag{1}$$

where

$$\left(\alpha, \vec{\beta}\right) \quad \text{(Fixed effects)} \tag{2}$$

$$\alpha_i \sim N(0, \sigma_i^2) \quad \text{(Country specific random intercept)} \tag{3}$$

$$\varepsilon_{i,t} \sim N\left(\sum_{m=1}^{3} \phi_m \varepsilon_{i,t-m}, \sigma_i^2\right) \quad \text{(Autocorrelated residuals as time random effects)} \tag{4}$$

$$\left(\sum_{p=1}^{3} \rho_p \Delta_t Y_{i,t-p}\right) \quad \text{(Autoregressive terms)} \tag{5}$$

'Fixed effect' is the equivalent of a non-random coefficient in a linear regression setting, and is estimated globally across all countries and time periods. The likelihood function for the data were all set to be Gaussian distributions for all of our metrics and models. Due to the instability of generating long-term forecasts using nonlinear models, we assumed linearity in the submodels, however, we incorporated nonlinear effects in our ensemble of submodels through features such as ARIMA terms, convergence terms, non-linear transformations, and forecasting in first-difference space. We checked for the stationarity of our retrospective GDP data to ensure that the time-series is not non-stationarity and to inform our forecast model specifications. We employed panel unit root tests sourced from a time-tested R library "plm". Two standard Unit Root tests for panel data were used: Levin-Lin-Chu and Maddala-Wu and we specified that the exogeneous variables were individual intercepts and time trends. The former yielded a p-value of 9.234e-4 and the latter yielded a p-value of less than 2.2e-16. These test results indicate that a rejection of the null hypothesis of non-stationarity.

S4.3.2 Covariates

Using our linear baseline model as defined in section S4.3.1, we created our set of ensemble sub-models by using all combinations of each of the covariates in Table S4.2. For example, if we were predicting GGE per

GDP with Educational attainment and Population as the predictors, then we would get a possible combination of four specifications to use (including one with no fixed effects). All covariates were included as fixed effects, and all specifications had the random effect on country intercepts. The proportion of submodels used that contain particular submodel specifications are described in Table S4.3.

S4.3.3 Convergence to Global Growth Rates

We believe that the very-long-term trajectory of a variable forecasted for each country will gradually taper to the global growth rates. The way that we implemented this growth rate convergence is in the following manner:

1) Suppose that we are forecasting $Y_{i,t}$, which includes an intercept at the global level and the country level. Let us also assume that there were $\widetilde{T}$ time periods of Y observed in the past for each country i.

2) We start the tapering of the growth rate to start from 10 years before $\widetilde{T}$ into the future starting from the present, converging to the global value 10 years after $\widetilde{T}$. Therefore, if the present time period is H, then our decyaing scheme will start off from the year $h + \widetilde{T} - 10$ and end at $h + \widetilde{T} + 10$.

3) Our main objective is to slowly taper all the countries' random intercepts to decay to zero, so that they are all following a global intercept. In a first-difference model, since the intercepts represent the linear time trend, this scheme will slowly converge the linear time trends of all countries to decay to the global time trend.

4) Starting from the first year of decay, we will transform the country specific intercepts as such:

$$\hat{\alpha}_i = (\hat{\alpha}_i) \times \frac{20 - t}{20}$$

   where t are all the time periods between $h + \widetilde{T} - 10$ and $h + \widetilde{T} + 10$.

5) By the time t reaches $h+\widetilde{T}+10$, the value of $\hat{\alpha}_i$ will have decayed to zero, and therefore, all the countries will have reached a global linear time trend estimate.

S4.3.4 Specifications

1) **ARIMA (Autoregressive Integrated Moving Average [7] [8]) terms**: We allowed up to three degrees of lags in the model (traditional auto-regressive terms), where each degree of AR term will include itself and all other lower degrees of lags. For example, a GDP per capita model with AR(3) specification (predicting log of GDP per capita) will include once, twice and thrice lagged log GDP per capita term. These were included as fixed effects.

   Additionally, in order to predict the best set of fixed effect coefficients, we test and include auto-correlated residuals in our models (traditional moving-average terms in an ARIMA setup). This basically means that we allow our models to estimate the residuals with an autoregressive process of their own. These were included as random effects, and we allowed this to exist at the country-year level.

All of the variables we forecasted used the first-differences transformation (across time) as the dependent variable, except DAH received which was forecasted in non-differenced space, because of the presence of very high noise, thereby making the differenced series very unstable to forecast.

2) **Recency weights**: One of the other specifications we included in our sub-models was the option of weighting the recent years higher. This is particularly helpful for countries like Ethiopia and Nigeria, where they had rapid economic growth in recent years, and we believe that is a better predictor of the GDP forecasts than the further past. The weighting function was defined as such:

$$Weight = f(\tau, t) = (T - t + 1)^{\tau} \tag{6}$$

where T is the final year of in-sample data we have, and t is the year at that data point. This is an exponential decaying weighting function, where the degree of decaying is determined by τ, and we test and include a set of values of τ ranging between 0 and 0.5, where $\tau = 0$ refers to equal weights (all time periods are weighed equally at 100). Given that $\left.\frac{df(\tau, t)}{dt}\right|_{\tau > 0} < 0$, this allows to weight our data with higher contributions to the likelihood from the recent past, and slowly decaying as we move further into the past towards the first observed time period. These recency weights were multiplied with the pointwise data variance parameter in order to weight the data points.

3) **Convergence Term**: We also allowed for the inclusion of a 'convergence term' in the list of sub-models. A convergence term is the one-year lag of the non-differenced dependent variable, and gets updated as each year is forecasted in the future. If a convergence term was considered in a sub-model, then we only included that sub-model if the coefficient on the convergence term was estimated to be negative (and statistically significant at 10% level).

S4.4 Inclusion and Exclusion Criteria

After we ran all possible combination of our sub-models and created a mean set of forecasts, we only want to keep the best possible set of sub-models. Hence, we implied the following set of inclusion and exclusion criteria in order to filter out the 'unrealistic' sub-models:

(1) All of the estimated coefficients must be **statistically significant at 10% level of significance**. For the fixed effects, we took the mean and the standard deviation of the posterior estimates, and filtered out the sub-model if the absolute z-score is below 1.645 (the absolute value of the one-sided 95th quantile of a standard normal distribution).

For the random effects, we look at the whether the measure of variance is statistically significant or not. The model outputs the mean and standard deviation of the *precision of the random effects*, and therefore allowed us to exclude the specification if the precisions are not statistically significant at 10% level.

(2) If there were any estimated coefficient that **defied a prior belief we have on the direction of the value**, then we dropped that sub-model from consideration. For example, we strongly believe that as a donor country's (high income countries) income (GDP per capita) grows, they will be able to donate more DAH to lower income countries, and so, if we ran a sub-model predicting DAH_d and get a negative coefficient on GDP per capita, we dropped that sub-model from consideration.

Our prior beliefs on the covariates for each dependent variable are listed in Table S4.2.

(3) The forecasted trajectory growth **must not exceed observed growth rates**. We believe that a country will not grow faster than how much it has grown in the past trend. In order to come up with the bounds, we run a stochastic frontier analysis (SFA) of the change in the predicted variable against the level value of the predicted variable. SFA is just like an ordinary least squares specification, except with the addition of an additional 'inefficiency' term with a half-normal distribution. This allows us to estimate (for example, for GDP per capita): conditional of a country's income, how much growth rate did we see in the country's GDP. We ran this analysis across all of the observed data points, and derived a relationship binding the growth rates of GDP against the absolute values of GDP.

S4.5 Creating the Forecasts

S4.5.1 Ranking Sub-Models

In order to find out with sub-models would be able to predict a country's future the best, we ran out-of-sample predictive validity (OOS-PV) tests [9]. Simply put: we took each sub-model that passed the criteria in section S4.4, and instead of running it on all of the past data, we left out some number of recent most years. Following that, we ran the sub-model on the truncated past, and forecasted those years left out. For example, our GDP data extends from 1970 through 2024; we left out 15 years of data, and reran a sub-model from 1970 - 2007, and use the results of that sub-model to forecast GDP for the out-of-sample years (2008 - 2024).

This gave us essentially two trajectories between 2008 through 2024: the truth and the out-of-sample predictions. For each year, we computed the squared error (the difference) between these two lines, and averaged these errors for each of the neighboring years. So for example, we had squared errors for 15 data points between 2007 and 2024, and so the first mean squared error sum was just the squared error at 2008 and 2009, the second was the mean of the sum of squared errors for 2008, 2009, and 2010, and so on. We then took the square root of this new series to get the running root mean squared errors (RMSE) for a given country.

We looked at a country's 2008 RMSE values for each sub-models ran, and listed out the best 10% of the sub-models (that is, the lowest 10% RMSE values), and we did so for every year out-of-sample. For a single country, we may potentially have completely different set of sub-models for each of the OOS years. Then, for the 10% of the sub-models selected in the *first* year OOS, we only used those models to predict the *first* year of forecast for each country; the set of 10% of the sub-models selected in the *second* year OOS were used to predict the *second* year of forecast for each country, and so on, until the last year OOS model selections are used to compute the forecast the remaining years. This allowed us to narrow down every country's trajectory with the best performing OOS-PV sub-models for each year.

S4.5.2 Uncertainty Estimation

To estimate the uncertainty intervals (UI), we reran the selected, ranked sub-models from section S4.5.1 and simulated draws instead of just getting a mean estimate of the future. There are four types of uncertainty we implemented in forecasting:

(i) **Model Uncertainty** : This type of uncertainty comes from having more than one type of specification to create forecasts, and therefore we included a set of sub-models in the ensemble to incorporate for this uncertainty (which are ranked within each country-year).

(ii) **Data Uncertainty** : If our covariates themselves had draws of the future data (for example, when we forecasted GDP as a covariate to forecast PPP), then we picked randomly from the draws of the independent variable when predicting a sub-model's trajectory, if that covariate was included.

(iii) **Parameter Uncertainty** : This type of uncertainty is due to the variance for the posterior distributions.

Once we have run a sub-model, we simulated from each of the estimated posterior distribution to create a set of simulated coefficients. This is done by simulating from the joint precision matrix of all the parameters estimated, and therefore produces a correlated set of coefficients.

(iv) **Fundamental Uncertainty** : The in-sample data and the fitted line in the past will never line up perfectly: there will always be errors from the model fit. We needed to reflect this level of uncertainty in our forecasts as well. By extracting these empirical residuals produced by a sub-model, we forecasted future country-specific residuals by using a random walk process, where the variance of the process is the variance of the residuals from the model fit σ_{ε}^2:

$$\hat{\varepsilon}_{i,t} \sim N(\varepsilon_{i,\hat{t}-1}, \sigma_{\varepsilon,t}^2) \tag{7}$$

A random walk is an AR(1) process with the coefficient equal to 1: in other words, the current value is independent of last year's value (except for the starting position), and will propagate forward with a random Gaussian noise of $\sigma_{\varepsilon,t}^2$ variance.

All of the above were used to simulate 500 forecasts (or draws), and so in order to construct our UIs, we took the mean and the values of the 2.5th and 97.5th quantiles to estimate 95% UIs.

Table S4.4 shows all of the retrospective and prospective estimates of our health spending outcomes in 2023 US dollars and 2023 purchasing-power parity-adjusted dollars in per capita space.

S4.6 Ad-hoc Draws Correlation

S4.6.1 Motivation

Given our current setup of compiling draws for each single year, there is no way of enforcing a temporal correlation across the draws right from the ensemble architecture. For example, the sub-models used in the first 15 years of GDP per capita forecasts were independently constructed and only depended on OOS-PV fits, while the 16th year onwards all draw from the same set of sub-models. This section details on the method used to generate the same correlation in the first 15 years of that example, drawing from the existing correlation from the 16th year onwards.

S4.6.2 Bivariate Correlated Distributions

Using the GDP per capita example: following from uncertainty estimation, once we generated approximately 500 forecast draws for a country and year for any of the covariates, we used the following strategy to achieve consistent temporal correlation across all time periods in the future:

(a) For each country, we recorded country-specific Spearman's correlation coefficient across all draws between 2038 and 2030, which gave us a country-specific correlation vector.

(b) For each value of correlation in step (a), we simulated a bivariate uniform distribution for each country and year (2025 through 2038). This simulated distribution was ranked in such a way that the marginal distributions in the joint distribution were correlated with that value of correlation coefficient we supplied (this joint distribution is known as a *copula*).

(c) We recorded the ranks of the copula, and sorted our draws (within each country) using those temporal ranks, and therefore we ended up with a complete time-series data for all draws, such that each country and year will follow the same rank correlation structure that exists between 2038 and 2030.

(d) Finally, we calculated our final set of uncertainty intervals by taking the 2.5th and 97.5th percentiles of these correlated draws.

We used this method at the end of forecasting every metric, since one metric fed into the other sequentially.

S4.7 Future Health Scenarios

We established the trajectories that our health expenditures are expected to take through 2030 using our ensemble models (from hereby referred to as the 'reference' case). The reference forecasts were built upon the basis of each country's past trends and expected future trends from covariates. We additionally also predicted what the possible trajectories for each country would look like if they were to follow the possible optimistic and pessimistic growth rates observed globally (referred to as 'greater' and 'lesser' cases, respectively).

S4.7.1 Long-term Growth Regressions

In order to determine what the possible greater and lesser growth rates for each country would be, we ran long-term growth regressions with the following specification:

$$Y_{i,T} - Y_{i,t} = \alpha + \beta Y_{i.t} + \epsilon_{i,t} \tag{8}$$

where the dependent variable represented the long term growth rate of Y for country i, which was computed either as logarithmic or logistic growth rates (for fractions).

The only independent variable we used ($Y_{i,t}$) was the value of Y_i at time t, and it served as a convergence term in this regression. This allowed us to predict the long-term growth rates of Y, conditional on a country's level of Y at time t.

S4.7.2 Forecasting Greater and Lesser Scenarios

In order to estimate what the future greater and lesser trajectories would be for each country, we followed these steps (assuming that we are forecasting from 2023 through 2030, with observed data between 1995 and 2022):

(i) We computed the 85th and 15th percentiles of the empirical residuals ϵ, as $Q_{0.85}(\hat{\epsilon_{i,t}})$ and $Q_{0.15}(\hat{\epsilon_{i,t}})$ respectively, where $Q_p(\cdot)$ is a quantile function for a percentile p.

(ii) We computed the starting annualized growth rate from the fitted scenario regression, such that, for country i:

$$\text{Greater growth rate} = \exp(\hat{\alpha}) \times \exp(Q_{0.85}(\hat{\epsilon_{i,t}})) \times (Y_{i,2022}^{\hat{\beta}})^{(1/(2022-1995))} \tag{9}$$

$$\text{Lesser growth rate} = \exp(\hat{\alpha}) \times \exp(Q_{0.15}(\hat{\epsilon_{i,t}})) \times (Y_{i,2022}^{\hat{\beta}})^{(1/(2022-1995))} \tag{10}$$

(iii) Finally, once we have established the growth rates as a function of the convergence term, we recursively created greater and lesser trajectories, conditional on the updated growth rates every year, such that:

$$Y_{i,t+1} = Y_{i,t} \times \exp(\hat{\alpha}) \times \exp(Q_{0.85}(\hat{\epsilon_{i,t}})) \times (Y_{i,t}^{\hat{\beta}}) \tag{11}$$

where $\exp(\hat{\alpha}) \times \exp(Q_{0.85}(\hat{\epsilon_{i,t}})) \times (Y_{i,t}^{\hat{\beta}})$ was the conditional growth rate for a single year.

One condition that we imposed for the computed scenarios is: the greater projection cannot be lower than the reference projection, and the lesser projection cannot be higher than the reference projection. For countries with wide forecasts where this case did happen, we moved the greater and lesser forecasts down and up to overlap on top of the reference line respectively.

A custom lesser scenario was also created based on projected DAH donated, under different assumptions about the future of health financing. Our baseline scenario of DAH forecasts, in which donors continue to prioritize health, was generated by assuming that donors will continue to allocate the same average portion of

ODA to health sector activities. Alternatively, our worse-case scenario of DAH forecasts, in which donors reallocate development assistance away from health, was generated by assuming that donors will reduce their portion of ODA to health sector activities by half.

S4.7.3 Uncertainty Estimation

The uncertainty intervals around a scenario were expected to take the same shape as the uncertainty around our reference forecasts. Therefore, once we have propagated a mean set of greater and lesser forecasts in section S4.7.2, we created the draws around our scenarios in the following way:

(i) We took our reference forecast's mean line and the 500 draws around that line.

(ii) We computed the deviation of the mean from each of the draws (in logarithmic or logistic transformation, depending on the space of the covariate).

(iii) We took each of the scenario mean lines and added the deviations from the previous step to the mean lines, giving us 500 draws of the scenario projections.

Figure set S4.3 visualizes the future health spending scenarios in per capita space (in 2023 US dollars).

S4.8 Package and Architecture

S4.8.1 Architecture

All analysis and forecasting were done on a parallel computing cluster with a CentOS interface. We compiled R [10] version 4.2.2 from source code on a Docker based on Debian OS, which was deployed as a Singularity container with all the necessary compilers and binaries (GCC, G++ and Fortran 8.2.0).

S4.8.2 Template Model Builder

We used the R library **TMB** [11] to run our baseline mixed effects models. TMB stands for Template Model Builder, and it's a powerful method of approximating the integral of the Gaussian probability distribution function (which doesn't exist in closed form) by using a Laplace Approximation, which gives very precise results, and is relatively faster than other approximation packages which exist like Stan or Bugs. TMB uses automatic differentiation on the negative log-likelihood function in order to optimize the model parameters, and also returns a sparse joint precision matrix (inverse of the variance-covariance matrix), from which we can simulate posterior parameter draws.

References

[1] GBD 2021 Risk Factors Collaborators. Global burden and strength of evidence for 88 risk factors in 204 countries and 811 subnational locations, 1990–2021: a systematic analysis for the Global Burden of Disease Study 2021. *The Lancet*, 403(10440):2162–2203, May 2024. doi: 10.1016/S0140-6736(24)00933-4. URL https://doi.org/10.1016/S0140-6736(24)00933-4.

[2] Spencer L James, Paul Gubbins, Christopher JL Murray, and Emmanuela Gakidou. Developing a comprehensive time series of gdp per capita for 210 countries from 1950 to 2015. *Population health metrics*, 10(1):12, 2012. URL https://pophealthmetrics.biomedcentral.com/articles/10.1186/1478-7954-10-12.

[3] Emmanuela Gakidou, Krycia Cowling, Rafael Lozano, and Christopher JL Murray. Increased educational attainment and its effect on child mortality in 175 countries between 1970 and 2009: a systematic analysis. *The Lancet*, 376(9745):959–974, 2010. URL https://doi.org/10.1016/S0140-6736(10)61257-3.

[4] GBD 2021 Demographics Collaborators. Global age-sex-specific mortality, life expectancy, and population estimates in 204 countries and territories and 811 subnational locations, 1950–2021, and the impact of the covid-19 pandemic: a comprehensive demographic analysis for the global burden of disease study 2021. *The Lancet*, 2024. URL https://doi.org/10.1016/S0140-6736(24)00476-8.

[5] Spencer Preston, Patrick Heuveline, and Michel Guillot. *Demography: measuring and modeling population processes*. Wiley-Blackwell, 1 edition. Malden, MA, USA, 2000.

[6] GBD 2021 Fertility and Forecasting Collaborators. Global fertility in 204 countries and territories, 1950–2021, with forecasts to 2100: a comprehensive demographic analysis for the global burden of disease study 2021. *The Lancet*, 2024. URL https://doi.org/10.1016/S0140-6736(24)00550-6.

[7] Vijay Kotu and Bala Deshpande. *Data Science: Concepts and Practice*. Morgan Kaufmann Publishers, 2nd edition. Cambridge, MA, USA, 2019.

[8] G. Box and G Jenkins. Time series analysis: Forecasting and control. 1970.

[9] Kyle J Foreman, Rafael Lozano, Alan D Lopez, and Christopher JL Murray. Modeling causes of death: an integrated approach using codem. *Population health metrics*, 10(1):1, 2012.

[10] R Core Team. *R: A Language and Environment for Statistical Computing*. R Foundation for Statistical Computing, Vienna, Austria, 2017. URL https://www.R-project.org/. Accessed: 2017-12-20.

[11] Kasper Kristensen, Anders Nielsen, Casper W Berg, Hans Skaug, and Brad Bell. Tmb: automatic differentiation and laplace approximation. *arXiv preprint arXiv:1509.00660*, 2015.

S4.9 Tables and Figures

Table S4.1: Definitions of health expenditure sources

Health Expenditure Type	Definition
Development assistance for health	Financial and in-kind contributions from global health channels that aim to improve or maintain health in low- or middle-income countries.
Government health expenditure as source	Comprises domestically financed government expenditure on health, including public health spending.
Out-of-pocket expenditure	Amount paid by individuals for health services.
Prepaid private health expenditure	Includes voluntary and compulsory prepayment, private insurance, and funding from non-governmental organizations.

Figure S4.1: State Space Diagram of Forecasting Components

This diagram details the order in which forecasting components are estimated, and which forecasting components are used as inputs in others.

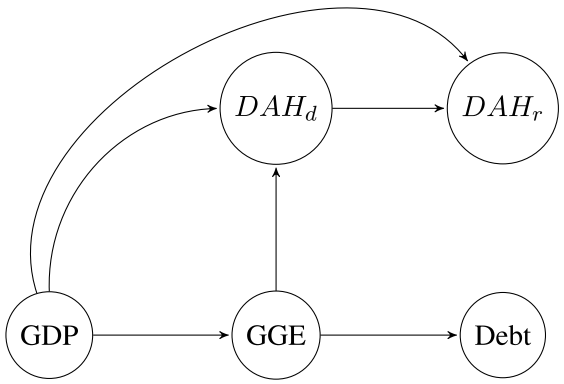

Table S4.2: Prediction and Covariates Map with Transformations

	**Predicted Variables**	**Covariates**	**Extra Specifications**
1	GDP per labour (log)	Education^{+}, Temperature, Temperature squared, Precipitation, Precipitation squared	
2	GGE per GDP (logit)	GDPpc^{+}, Pop >64^{+}, Education^{+}	ARIMA(0-3, 1, 0-3)
2	Debt ratio (logit)	GDPpc^{+}, Pop >64^{+}, Education^{+}	ARIMA(0-3, 1, 0-3)
3	DAH donated	-	
4	DAH received per total DAH donated (logit)	GDPpc^{-}, Pop <15^{+}, Ebola dummy^{+}, total DAH envelope	ARIMA(0, 0, 0)

Health Spending estimates from 1995-2030

All estimates produced for this publication can be found online in the Global Health Data Exchange (GHDx) at `https://ghdx.healthdata.org/series/financing-global-health-fgh`. The data included shows all of our health spending outcomes for every country and year from 1995 through 2030, and include 95% uncertainty intervals. The variables are in per capita measures, and for currencies 2023 US dollars and 2023 purchasing-power parity-adjusted dollars,.

Figure S4.2: Process diagram for estimating future GDP, all-sector government spending, and health spending by source

This diagram details the inputs and outputs of the ensemble modeling process.

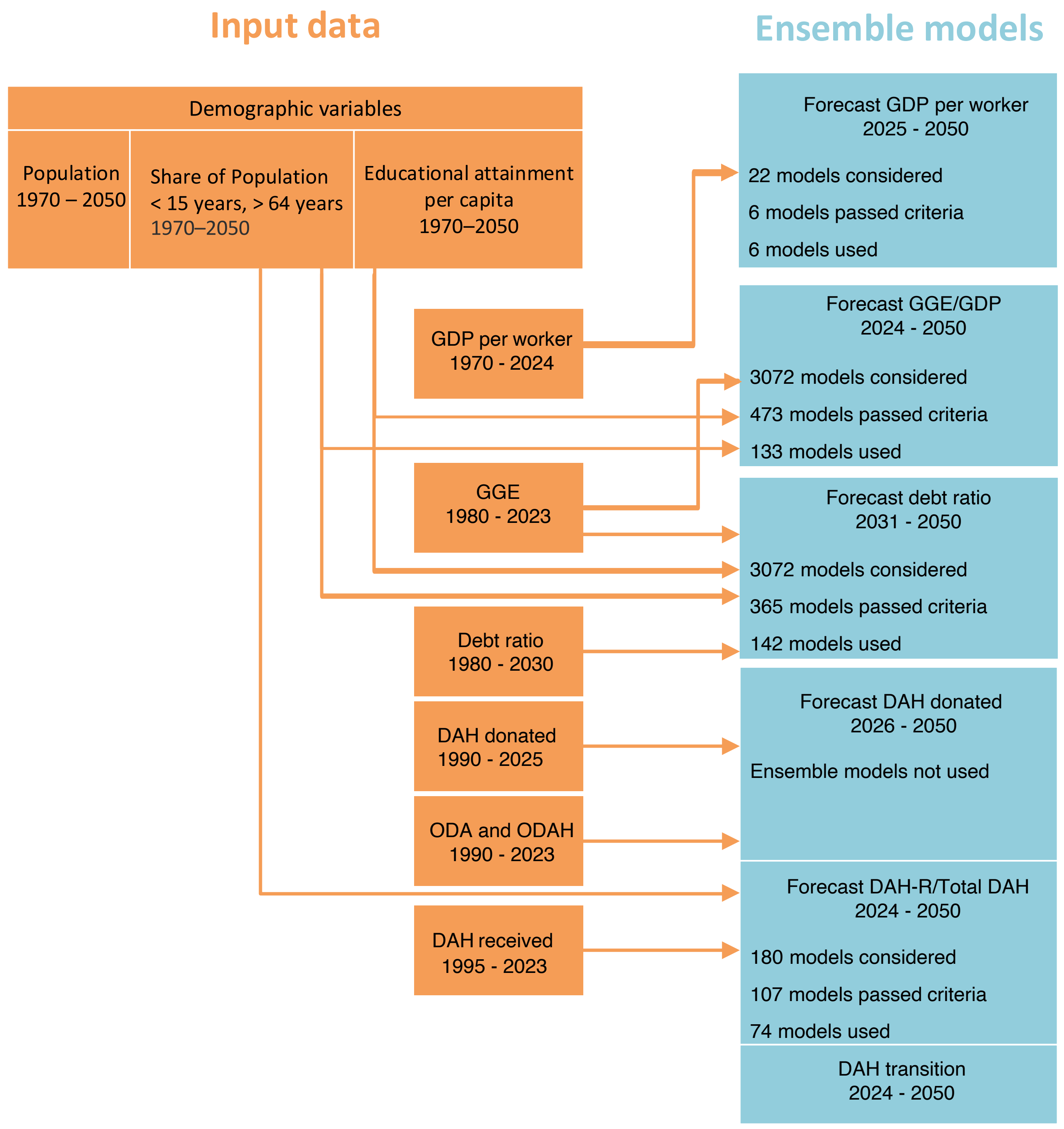

Additional covariates:

- All models are estimated using first-order differences and country-specific random intercepts
- All ensemble models considered up to three degrees of autoregressive terms
- GDP per capita and GGE ensemble models considered a convergence term, up to three degrees of auto-correlation, and twelve potential weighting schemes across recent years and observations

Table S4.3: Ensemble submodel specifications used in forecasts

This table shows all submodel specifications considered in the ensemble, and the percentage of models with given specifications used in the forecasts.

	GDP		GGE		Debt ratio		DAH Received	
Number of out-of-sample years included	15		15		15		10	
Number of countries included	204		204		204		131	
Number of top models used per country-year	6		17		22		15	
Total number of country-year-models used	18360		52020		67320		19650	
Retrospective data years used for model fitting	1950-2025 (for forecasted years >2026)		1980-2019 (for forecasted years >2023)		1980-2030		1990-2023 (for forecasted years >2023)	
Covariate(s)	Value	Percentage of models	Value	Percentage of models	Value	Percentage of models	Value	Percentage of models
Autoregressive terms	0	100.0%	3	17.5%	3	2.8%	0	100.0%
			2	7.7%	2	8.2%		
			1	25.7%	1	15.6%		
			0	49.0%	0	73.4%		
Moving average terms	0	100.0%	3	20.7%	3	19.7%	0	100.0%
			2	26.3%	2	28.6%		
			1	25.9%	1	37.5%		
			0	27.0%	0	14.2%		
Scaled level convergence term	Included	16.7%	Included	32.3%	Included	61.3%	Not included	100.0%
	Not included	83.3%	Not included	67.7%	Not included	38.7%		
Recency weights (Values represent the tau value used in our recency weighting function described on page 10 of this appendix. Higher values indicate a stronger weight placed on recent years' data.)	0	100.0%	0.7	13.9%	0.7	24.5%	0.8	3.6%
			0.636363636	12.9%	0.63636364	14.9%	0.77241	3.6%
			0.57273	9.8%	0.57273	9.7%	0.74483	3.4%
			0.50909	9.9%	0.50909	6.0%	0.71724	3.5%
			0.44545	12.5%	0.44545	4.8%	0.68966	3.5%
			0.38182	9.6%	0.38182	6.0%	0.66207	3.9%
			0.31818	7.5%	0.31818	6.2%	0.63448	3.9%
			0.25455	4.5%	0.25455	5.3%	0.60690	3.8%
			0.19091	4.9%	0.19091	5.6%	0.57931	3.8%
			0.12727	5.3%	0.12727	5.9%	0.55172	3.7%
			0.06364	4.5%	0.06364	5.7%	0.52414	3.8%
			0.00000	4.8%	0.00000	5.5%	0.49655	3.8%
							0.46897	3.8%
							0.44138	3.6%
							0.41379	3.5%
							0.38621	3.3%
							0.35862	3.8%
							0.33103	3.9%
							0.30345	3.0%
							0.27586	2.9%
							0.24828	2.8%
							0.22069	2.8%
							0.1931	2.8%
							0.16552	2.8%
							0.13793	2.7%
							0.11034	2.6%
							0.08276	2.5%
							0.05517	2.6%
							0.02759	2.6%
							0	3.6%
Proportion of population under age 15							Included	1.0%
							Not included	99.0%
Proportion of population over age 65			Included	0.0%				
			Not included	100.0%				
GDP per capita			Included	0.0%	Included	62.1%	Included	100.0%
			Not included	100.0%	Not included	37.9%	Not included	0.0%
Educational attainment per capita	Included	50.0%	Included	0.8%	Included	0.0%		
	Not included	50.0%	Not included	99.2%	Not included	100.0%		
